# Efficacy of supplementation with nutrients and dietary supplements on recovery and inflammatory response among patients with COVID-19: a network meta-analysis

**DOI:** 10.3389/fnut.2026.1825539

**Published:** 2026-07-28

**Authors:** Jin Zhao, Jiayi Chang, Jianghao Qu, Fengyuan Li, Shiyang Zhang, Yuying Yang, Tianjiao Zhao

**Affiliations:** Clinical School, North Henan Medical University, Xinxiang, Henan, China

**Keywords:** COVID-19, dietary supplements, network meta-analysis, nutritional supplement, randomized controlled trial

## Abstract

**Background:**

At present, the clinical course of COVID-19 is synergistically influenced by the systemic inflammatory storm and nutritional deterioration triggered by COVID-19. Nutrition interventions have demonstrated potential clinical value.

**Objective:**

This study aims to systematically compare the relative efficacy of different dietary patterns and nutritional supplements on key outcomes among adult patients with COVID-19, and to identify the optimal regimen of nutrition intervention.

**Methods:**

PubMed, Embase, Cochrane Library, and Web of Science, were systematically searched. Randomized controlled trials (RCTs) were independently screened. The risk of bias was assessed using the Cochrane Risk of Bias 2.0 (RoB 2.0) tool. The inflammation status of patients was measured by alterations in indicators such as interleukin-6 (IL-6) and neutrophil-to-lymphocyte ratio (NLR), while recovery was evaluated based on changes in the number of patients with adverse complications, including cough and death. Statistical analysis was performed in R (version 4.5.1). A random-effects model under the Bayesian framework was adopted to pool mean differences (MDs) or risk ratios (RRs) alongside their corresponding 95% credible intervals (CrIs). Additionally, regression analyses were performed for sample size, treatment duration, and disease severity of patients. The confidence in the evidence was assessed using the Confidence in Network Meta-Analysis (CINeMA).

**Results:**

The efficacy of interventions varied for different immune and clinical outcomes among patients with COVID-19. Compared with standard of care (SoC) alone, vitamin C + SoC significantly decreased IL-6(vitamin C + SoC vs. SoC alone: MD = −88.77, 95% CrI: −139.02, −38.66, CINeMA: low certainty). Compared with SoC, COVID-19 patients supplemented with nanocurcumin+SoC showed a significant increase in lymphocyte count (nanocurcumin + SoC vs. SoC: MD = 4.76, 95% CrI: 2.91, 6.62, CINeMA: NA). In comparison with SoC, COVID-19 patients supplemented with pomegranate juice exhibited a marked increase in NLR (pomegranate juice + SoC vs. Placebo: MD = 1.04, 95% CrI: 0.21, 1.88, CINeMA: very low certainty).

**Conclusion:**

Compared with SoC alone, significant changes were observed in IL-6, NLR, and lymphocyte count among patients suffering from COVID-19 following supplementation with nutrients and dietary supplements.

**Systematic review registration:**

PROSPERO (CRD420251110943).

## 1 Introduction

COVID-19 is an acute respiratory infectious disease caused by SARS-CoV-2, which has evolved into a global public health crisis since its outbreak in late 2019. As of May 2023, over 765 million confirmed cases and over 6.9 million deaths have been reported worldwide (1). Critically ill patients with COVID-19 tend to develop acute respiratory distress syndrome (ARDS) and multiple organ failure due to excessive inflammatory responses. The demand for resources such as ventilators and intensive care beds from such patients has soared, directly resulting in shortages of critical medical resources in many regions globally. This not only significantly aggravates the operational and economic burden on healthcare systems but may also compromise the quality of care due to constraints in resource allocation (2). The health damage caused by the virus persists into the recovery phase. A follow-up study on patients recovering after hospitalization indicates that 49% of affected patients experience multi-system long-term discomforts such as fatigue, sleep disturbances, and abnormal inflammatory markers within one year after discharge (3). Large longitudinal cohorts have confirmed the presence of persistent somatic symptoms in COVID-19 survivors, and the World Health Organization has issued unified clinical diagnostic criteria to classify these persistent symptoms as an independent clinical condition, namely post-COVID-19 condition (long COVID) (3, 4). Such long-term symptoms continuously lower patients’ quality of life and substantially increase long-term medical expenditures. Therefore, exploring interventions that improve recovery and modulate inflammatory responses is essential to alleviate both short-term treatment pressures and long-term societal burdens of COVID-19.

Dietary components and nutritional supplements, leveraging their anti-inflammatory and immunomodulatory effects, have become a research hotspot for adjunctive interventions in COVID-19, and multiple supplements have been proven to be of clinical value. Among them, n-3 polyunsaturated fatty acids (EPA, DHA) reduce systemic inflammation levels by regulating inflammatory signaling pathways and inhibiting the release of pro-inflammatory mediators (5). Although vitamin C has not been shown to significantly lower mortality rates, high-dosage supplementation shortens the duration of fever and respiratory symptoms in mild cases by improving oxidative stress (6). Vitamin D is crucial for maintaining normal immune responses, and patients with baseline vitamin D deficiency face a significantly increased risk of severe illness and death (7). Melatonin inhibits airway and central nervous system inflammation, effectively relieving cough and reducing cough hypersensitivity (8). Pomegranate juice, rich in polyphenols, reduces inflammation-immunity imbalance indicators such as NLR, alleviates systemic chronic low-grade inflammation, and thereby mitigates inflammation-related immune dysregulation (9). The above evidence suggests that these nutritional interventions can be potentially applied in the recovery of patients suffering from COVID-19.

At present, shortcomings are still observed in systematic reviews and meta-analyses on nutritional interventions for patients suffering from COVID-19, limiting their utility in clinical practice. First, most studies focus on a single nutrient, lacking a horizontal comparison of multiple interventions, which prevents clarifying the relative advantages of different supplements (10). Second, existing meta-analyses inconsistently define outcome indicators. Some concentrate on hard endpoints such as mortality rate and hospitalization rate, while others only evaluate subjective indicators such as symptom relief. Moreover, integrated analysis of core inflammatory markers such as C-reactive protein (CRP) and interleukin-6 (IL-6) is insufficient, leading to variability in evidence interpretation (11). Third, significant controversies and evidence gaps exist in current research. For instance, several meta-analyses have reported no statistically significant increase in mortality risk among critically ill COVID-19 patients receiving vitamin C supplementation (6, 12, 13). However, studies on other interventions, such as N-acetylcysteine, are limited by a small sample size, and their reliability remains to be validated (14). Fourth, traditional meta-analyses predominantly rely on direct comparison data and insufficiently integrate indirect evidence systematically, resulting in an incomplete assessment of intervention effects.

This study, through a network meta-analysis (NMA), endeavors to precisely reduce the limitations of existing research. Its core advantage lies in overcoming the constraints of traditional meta-analysis. By simultaneously integrating evidence from both direct and indirect comparisons, this study ranks the quantitative efficacy of multiple nutrition interventions, thereby providing more comprehensive evidence to support clinical decision-making. This study focuses on a set of promising interventions, including fatty acid, vitamin C, vitamin D, melatonin, and pomegranate juice, and systematically assesses their effects on both ‘recovery outcomes’ (e.g., symptom relief time and functional recovery rate) and ‘inflammatory markers’. The aim is to address the issues of inconsistent indicators and inadequate comparisons in current research. Compared with published studies, the innovation of our study lies in a broader coverage of intervention types. This study endeavors to clarify the practical interventional performance of different diets and nutritional supplements for individuals suffering from COVID-19, thereby providing high-quality evidence-based support for selecting an optimal adjuvant treatment regimen in clinical practice and further refining the comprehensive treatment system for COVID-19.

## 2 Methods

This study was performed adhering to the Preferred Reporting Items for Systematic Reviews and Meta-Analyses (PRISMA) guidelines and their extensions for an NMA.^1^ This study protocol was registered on the International Prospective Register of Systematic Reviews (PROSPERO) (Registration number: CRD420251110943).

### 2.1 Literature search

Four databases, comprising PubMed, Embase, Cochrane Library, and Web of Science, were systematically searched from database inception up to July 22, 2025. The search strategy employed a combination of Medical Subject Headings (MeSH) terms and free-text terms, including ‘COVID-19’, ‘Dietary Supplements’, ‘Randomized Controlled Trial’, ‘Nutritional Supplement’, and ‘Coronavirus Disease 2019’. Furthermore, the reference lists of relevant literature and grey literature were manually screened to identify additional eligible studies. The search strategy is detailed in Supplementary material 1.

### 2.2 Inclusion and exclusion criteria

Studies meeting the following criteria were included. (i) Population: Participants met the diagnostic criteria for COVID-19, and the diagnostic basis must clearly refer to authoritative guidelines (e.g., the diagnostic criteria for COVID-19 issued by the World Health Organization, the National Health Commission of the People’s Republic of China, and the U. S. Centers for Disease Control and Prevention). (ii) Intervention & Comparison: The intervention consisted of nutritional or dietary supplementation added to standard of care (SoC), compared against SoC alone. (iii) Study Design: Studies were randomized controlled trials (RCTs). (iv) Outcome Indicators (inflammation): Studies reported IL-6, lymphocyte count, neutrophil-to-lymphocyte ratio (NLR), CRP, white blood cell (WBC) count, or neutrophil count. (v) Outcome Indicators (adverse complication): Studies reported the number of patients with cough, the number of deaths, the number of patients with headache, the number of patients with fever, and the number of patients with dyspnea.

Studies were excluded if they were: (i) animal or cell studies, case reports, study protocols, commentaries, letters, editorials, or conference proceedings, (ii) publications with missing data or significant errors, (iii) duplicate publications, (iv) studies from which a full text was not available, or (v) studies involving overlapping participant cohorts.

### 2.3 Data extraction

Retrieved records were imported into EndNote. Two investigators (Shiyang Zhang and Jianghao Qu) independently screened the titles and abstracts against the inclusion and exclusion criteria. Subsequently, the full texts of the remaining literature were reviewed for a second round of screening. Any discrepancy was reconciled through discussion or consultation with a third investigator (Fengyuan Li). The two researchers independently extracted data using a pre-designed spreadsheet, including first author, publication year, country, randomization and blinding design, intervention and control measures, treatment duration, baseline characteristics of participants, and outcome indicators.

### 2.4 Quality assessment

Two investigators (Jin Zhao and Yuying Yang) independently assessed the quality of the eligible studies using the Cochrane Risk of Bias 2.0 (RoB 2.0) tool. The RoB 2.0 criteria encompass five domains: bias arising from the randomization process, bias due to deviations from the intended interventions, bias due to missing outcome data, bias in measurement of the outcomes, and bias in selection of the reported results. Each domain is rated as having a ‘high risk’, ‘some concerns’, or a ‘low risk’ of bias. For the overall study assessment, a study is rated to have a low risk of bias if all domains are low-risk, or if only one domain raises some concerns with the rest being low-risk. A study is judged as having a high risk of bias if at least one domain is high-risk or if multiple domains (four or more) raise some concerns. All other cases are assessed as having some concerns. The quality of all the eligible studies was independently assessed by the two investigators, and any discrepancy was reviewed and reconciled by a third author (Jiayi Chang).

### 2.5 Statistical analysis

The inflammation status of patients was measured by alterations in inflammation-related markers such as CRP, IL-6, and NLR. The recovery of patients was evaluated by changes in the number of adverse complications, including cough and death. The outcome indicators from the included studies consisted of continuous variables and dichotomous variables. Mean difference (MD) or risk ratio (RR) alongside 95% credible interval (CrI) was used as a measure for the effect size. An NMA model based on a Bayesian framework was constructed using the Markov Chain Monte Carlo method, and the model was iterated to estimate the relative efficacy of different treatment regimens. During the process of model inspection, the chain number was set to 4, the annealing value to 10,000, and the iteration count to 50,000, with a step size of each inspection of 1. This process was implemented to obtain the posterior distribution. NMA is performed relying on three fundamental assumptions, comprising transitivity, homogeneity, and consistency. Overall, I^2^ < 50% indicated that heterogeneity among included studies for the same comparison was considered acceptable, thus satisfying the homogeneity assumption. Inconsistency between the direct and indirect comparisons was examined using the node-splitting method, and *p* > 0.05 indicated no significant inconsistency, fulfilling the consistency assumption. Convergence of the results was assessed by operating potential scale reduction factors (PSRFs), with 1 ≤ PSRF<1.05 representing successful convergence. A network structure was constructed where the relationships of the standardized mean differences (SMDs) among all interventions were treated as lines and the interventions themselves as nodes. In this network structure, nodes were utilized to denote different interventions and edges to represent head-to-head comparisons between interventions. The cumulative ranking probability plots were analyzed to estimate all cumulative ranking probabilities, subsequently summarized and reported as surface under the cumulative ranking curve (SUCRA). Potential publication bias was evaluated using funnel plots. All the aforementioned statistical analyses were performed using R (version 4.5.1) and Stata (version 15.0).

### 2.6 Certainty assessment

The Confidence in Network Meta-Analysis (CINeMA) (available at http://cinema.ispm.ch) was employed to assess the quality of evidence for all pairwise comparisons. This tool is a methodological framework for assessing the reliability of NMA results (15). The CINeMA covers six key domains: within-study bias (risk of bias), publication or reporting bias, indirectness, imprecision, heterogeneity, and incoherence. Depending on the extent of bias, each domain is classified into three categories: no concerns (no downgrading), some concerns (downgraded by one level), and major concerns (downgraded by two levels). Finally, the certainty for each pairwise comparison was rated as ‘high’, ‘moderate’, ‘low’, or ‘very low’.

## 3 Results

### 3.1 Literature search and screening

Totally 6,342 records were identified. After removing 1,923 duplicates, the titles and abstracts of the remaining literature were assessed, and 1,743 were excluded. The full texts of the remaining studies were reviewed against the pre-defined inclusion and exclusion criteria. Ultimately, 45 studies were included. The screening process is detailed in Supplementary Figure S1.

The 45 included studies were performed in 18 countries (Iran, Pakistan, Russia, Italy, Thailand, Croatia, America, Switzerland, India, Iraq, China, Tunisia, Philippines, Brazil, Mexico, Argentina, the Netherlands, and Greece), involving 3,793 patients, of whom 55.38% were male and 44.62% female. The age range of participants was from 34.53 to 69 years. The interventions investigated included various dietary and nutritional supplements, such as fatty acids, vitamin C, vitamin D, nanocurcumin, melatonin, pomegranate juice, as well as a compound preparation of L-arginine, nucleotides, fatty acids, and cysteine.

All interventions were administered based on SoC, with treatment durations varying among the included RCTs. The reported outcome indicators included inflammation-related markers (CRP, IL-6, lymphocyte count, NLR, neutrophil count, and WBC count) and indicators for adverse complications (number of patients with cough, fever, headache, and dyspnea, and number of deaths). The basic characteristics of the included RCTs are summarized in Supplementary Table S1.

The risk of bias assessment for the included studies indicated that eight studies were judged as having some concerns, primarily due to the following reasons: (i) Care providers or trial personnel were aware of group allocation during the trial; (ii) The analysis methods for assessing intervention effects were inappropriate; (iii) There was no difference in measuring or ascertaining the outcome between groups. Thirteen studies were rated as having a high risk of bias, mainly for two reasons: (i) Participants were aware of their own group allocation during the trial; (ii) The analysis methods for evaluating intervention effects were inappropriate. The quality assessment is illustrated in Supplementary Figure S2.

### 3.2 Results of NMA

#### 3.2.1 Network diagram

In the network diagram, each node represents an intervention. The area of a node is positively associated with the number of studies involving that intervention, with a larger node representing more studies. The line between two nodes indicates the presence of studies directly comparing the two interventions. The thickness of the line corresponds to the number of studies for that direct comparison, with a thicker line standing for more relevant studies. The deviance information criterion (DIC) differences for all outcome models were less than 5, indicating overall model consistency (Supplementary Table S2). The PSRF values for all outcome models were equal to 1, confirming that all models successfully converged (Supplementary material 3).

#### 3.2.2 Summary of outcome indicators

##### 3.2.2.1 IL-6

**Figure 1:**
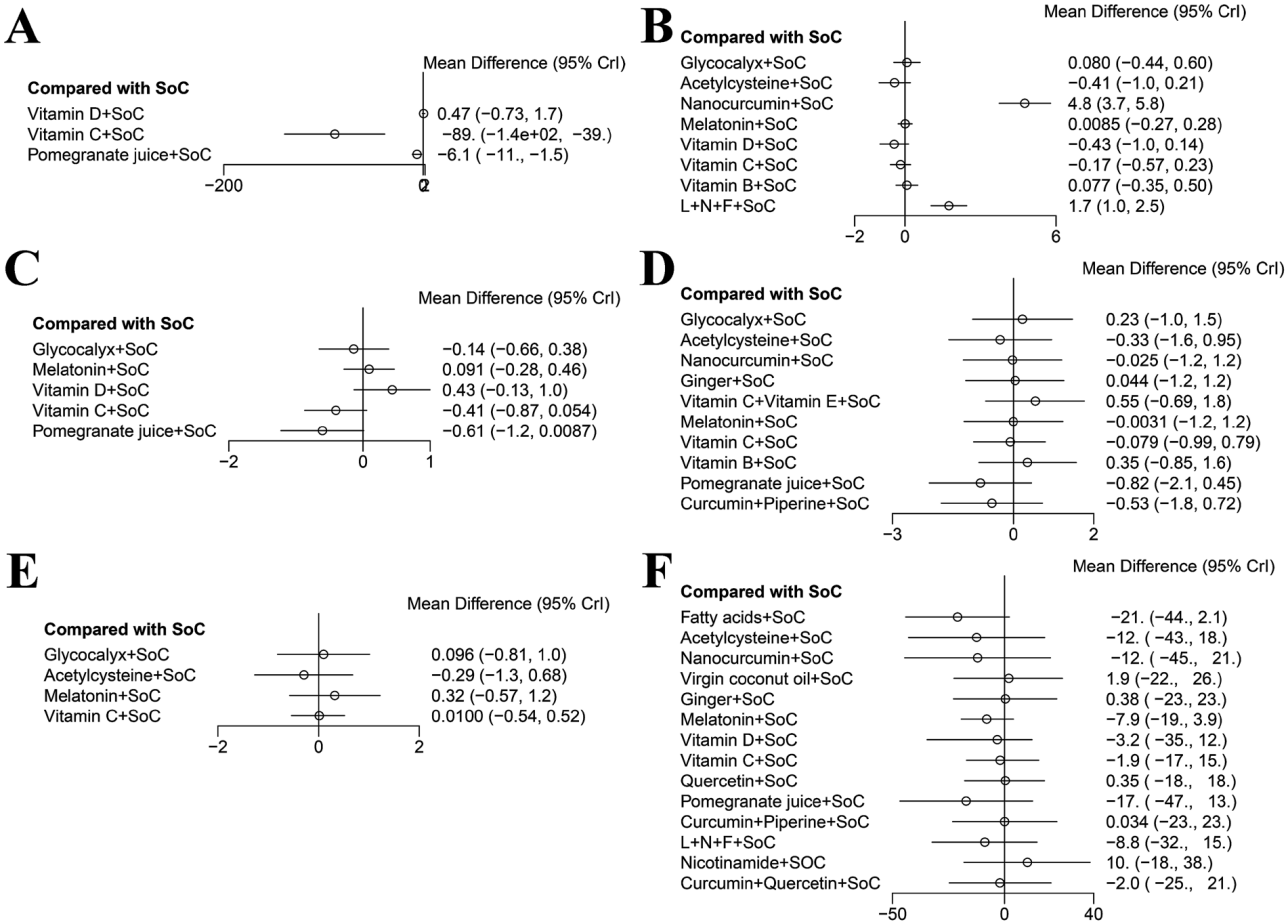
Forest plots (the diamond symbol represents the summary effect size with 95% CI, and squares denote individual study effects. Heterogeneity was assessed using the *I*^2^ statistic and chi-square test, with a reference line indicating no effect) for **(A)** IL-6, **(B)** lymphocyte count, **(C)** NLR, **(D)** WBC count, **(E)** neutrophil count, and **(F)** CRP.

**Figure 2:**
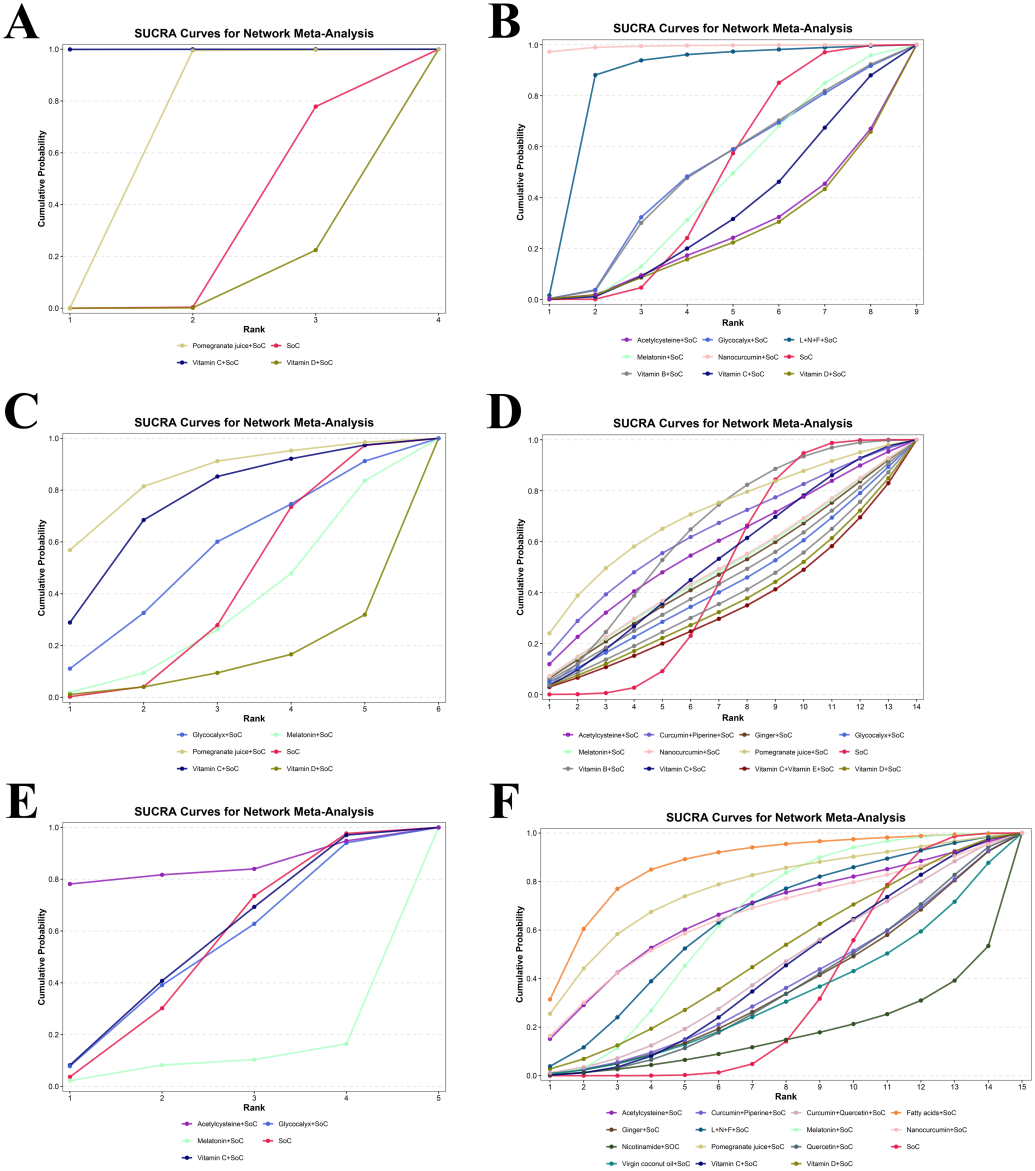
SUCRA ranking plots (bar height denotes the SUCRA value, where higher values signify superior treatment ranking. This is estimated based on a specified model, with cumulative probability curves superimposed to visualize ranking distributions) for **(A)** IL-6, **(B)** lymphocyte count, **(C)** NLR, **(D)** WBC count, **(E)** neutrophil count, and **(F)** CRP.

Six RCTs provided data on IL-6, covering 268 participants (ΔDIC = 0.09, I^2^ = 11%). The NMA revealed that in comparison with SoC alone, supplementation with vitamin C combined with SoC resulted in a pronounced decrease in the inflammatory factor IL-6 among patients suffering from COVID-19 (vitamin C + SoC vs. SoC alone: MD = −88.77, 95% CrI: −139.02, −38.66, CINeMA: low certainty). The details are shown in Supplementary Table S3 and Supplementary material 4, and the forest plot is provided in Figure 1A. The SUCRA probability ranking was as follows: vitamin C + SoC (99.96%) > pomegranate juice + SoC (66.44%) > vitamin D + SoC (7.53%). Vitamin C + SoC was the optimal regimen in reducing IL-6 levels (Figure 2A).

##### 3.2.2.2 Lymphocyte count

In total, 11 RCTs reported lymphocyte count, covering 650 patients (ΔDIC = 0.02, I^2^ = 34%). The NMA results indicated that, compared with SoC alone, COVID-19 patients supplemented with nanocurcumin+SoC showed a significant increase in lymphocyte count (nanocurcumin+SoC vs. SoC: MD = 4.76, 95% CrI: 2.91, 6.62, CINeMA: moderate certainty). The details are shown in Supplementary Table S4 and Supplementary material 4. The forest plot is presented in Figure 1B. The SUCRA probability ranking was: Nanocurcumin+SoC (99.43%) > L + N + F + SoC (84.42%) > Vitamin B + SoC(48.46%). Nanocurcumin+SoC was the most effective regimen for increasing lymphocyte count (Figure 2B).

##### 3.2.2.3 NLR

Six RCTs provided data on NLR, with 341 patients involved (ΔDIC = 0.01, I^2^ = 8%). The NMA results showed that, in comparison with SoC alone, COVID-19 patients supplemented with pomegranate juice + SoC experienced a significant increase in NLR (pomegranate juice + SoC vs. SoC: MD = 1.04, 95% CrI: 0.21, 1.88, CINeMA: very low certainty). The details are displayed in Supplementary Table S5 and Supplementary material 4. The forest plot is provided in Figure 1C. The SUCRA probability ranking results were as follows: pomegranate juice + SoC (90.16%) > vitamin C + SoC (79.46%) > glycocalyx+SoC (54.70%). Pomegranate juice + SoC was the optimal regimen for elevating NLR (Figure 2C).

##### 3.2.2.4 WBC count

Fourteen RCTs reported WBC count, covering 656 patients (ΔDIC = 0.14, I^2^ = 14%). The overall heterogeneity was low, and a random-effects model was applied. The NMA results demonstrated that, compared with SoC alone, dietary supplementation exerted no significant effect on WBC count among patients with COVID-19. The results are detailed in Supplementary Table S6. The forest plot is presented in Figure 1D. The SUCRA probability ranking results showed: pomegranate juice + SoC (84.13%) > curcumin+piperine+SoC (73.91%) > acetylcysteine+SoC (64.63%). Pomegranate juice + SoC was the most effective in reducing WBC count (Figure 2D).

##### 3.2.2.5 Neutrophil count

In total, 6 RCTs provided data on neutrophil count, with 349 patients covered (ΔDIC = 0.04, I^2^ = 27%). The overall heterogeneity was low, and a fixed-effects model was adopted. The NMA results showed that, in comparison with SoC alone, dietary supplementation exerted no significant effect on neutrophil count among patients with COVID-19. The details are shown in Supplementary Table S7, and the forest plot is provided in Figure 1E. The SUCRA probability ranking results were as follows: acetylcysteine+SoC (75.97%) > SoC (53.71%) > vitamin C + SoC (52.23%). Acetylcysteine + SoC was superior in reducing neutrophil count (Figure 2E).

##### 3.2.2.6 CRP

Twelve RCTs reported CRP, covering 1,354 patients (ΔDIC = 0.09, I^2^ = 10%). The overall heterogeneity was low, and a fixed-effects model was applied. The NMA results showed that, compared with SoC alone, dietary supplementation exerted no significant effect on CRP among COVID-19 patients. The results are detailed in Supplementary Table S8, and the forest plot is presented in Figure 1F. The SUCRA probability ranking results showed: fatty acids + SoC (86.67%) > pomegranate juice + SoC (76.62%) > acetylcysteine+SoC (67.31%). Fatty acids + SoC was the most effective regimen for reducing CRP (Figure 2F).

##### 3.2.2.7 Number of patients with headache

**Figure 3:**
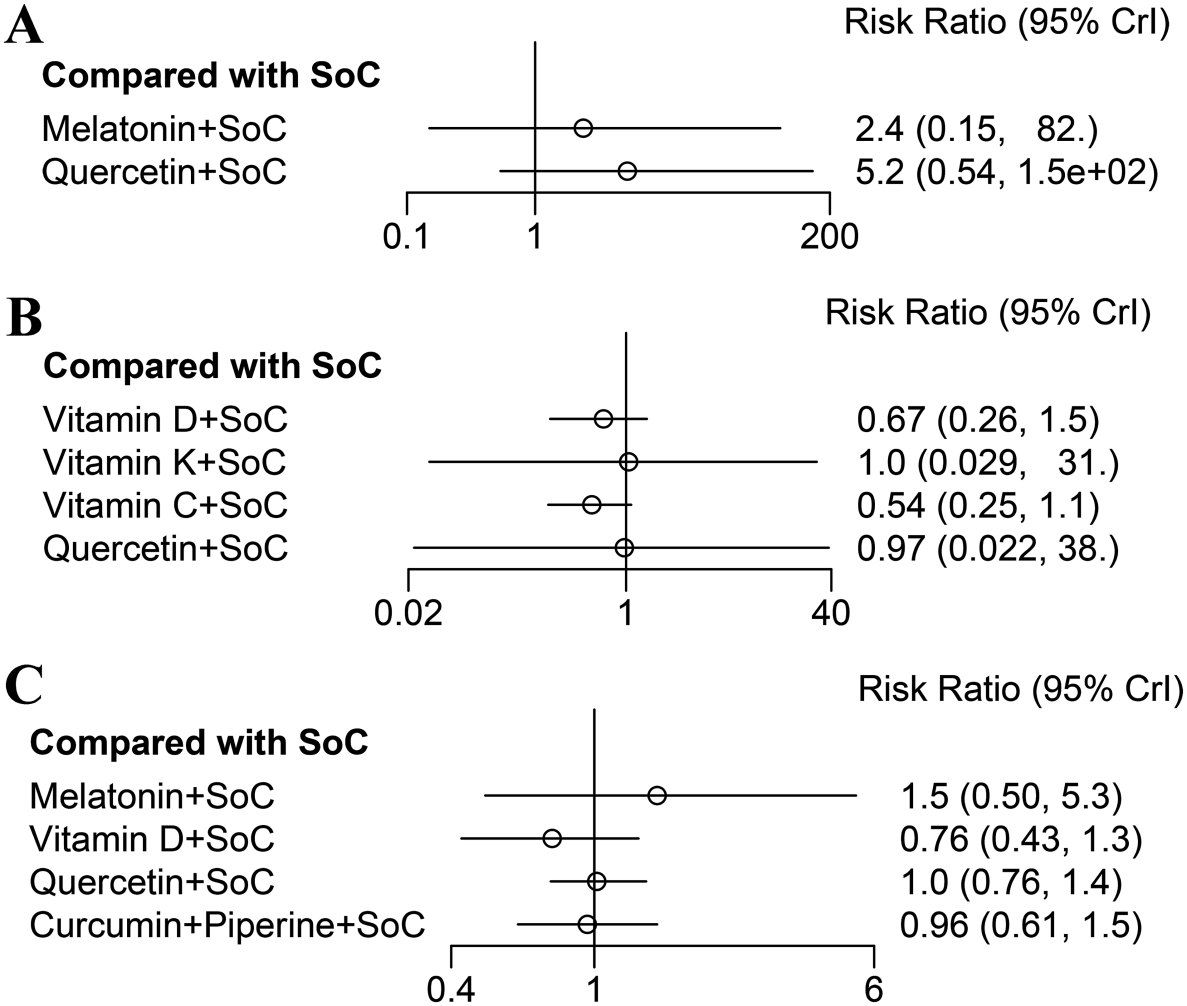
Forest plots (the diamond symbol represents the summary effect size with 95% CI, and squares denote individual study effects. Heterogeneity was assessed using the I^2^ statistic and chi-square test, with a reference line indicating no effect) for **(A)** Number of patients with headache, **(B)** Number of deaths, and **(C)** Number of patients with fever.

**Figure 4:**
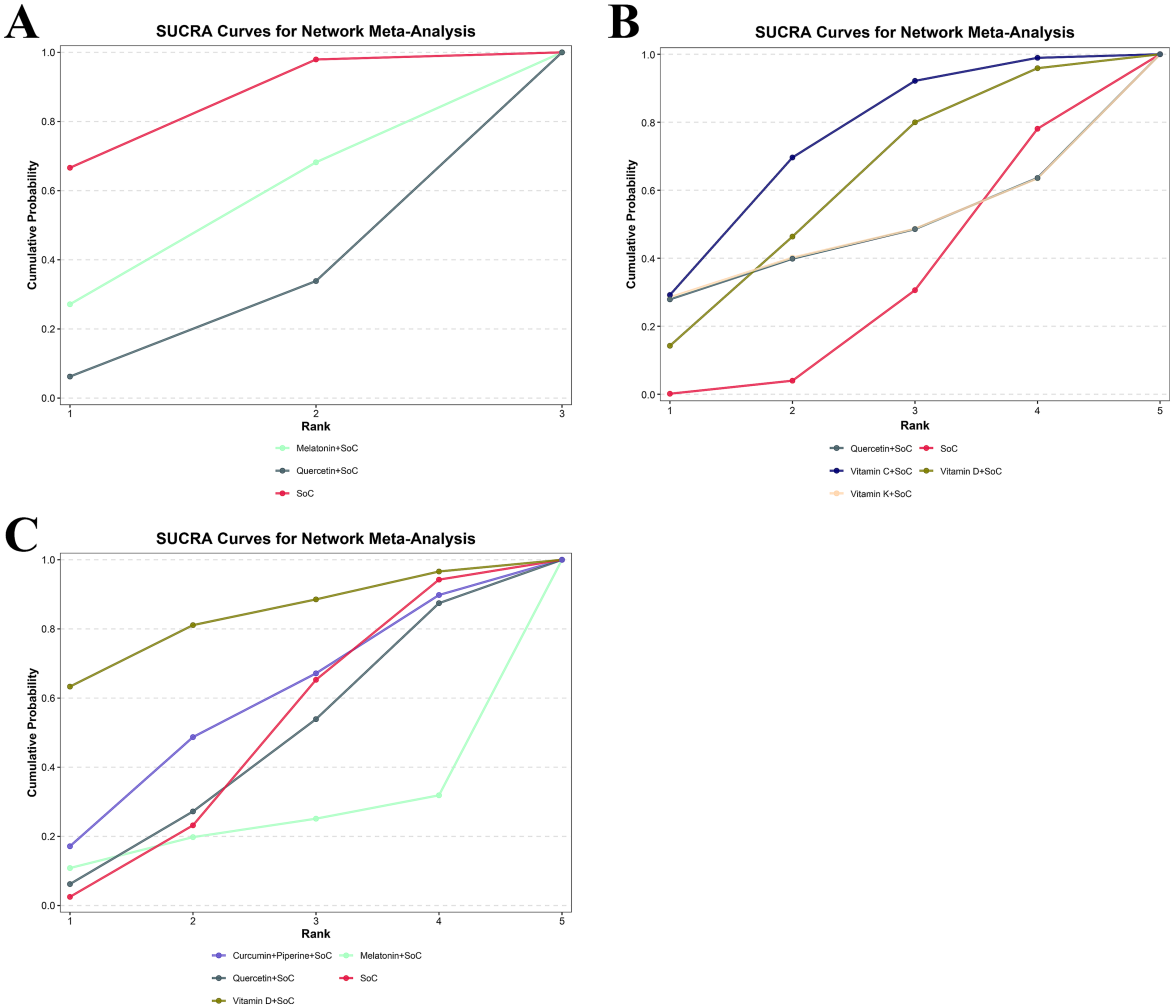
SUCRA ranking plots (bar height denotes the SUCRA value, where higher values signify superior treatment ranking. This is estimated based on a specified model, with cumulative probability curves superimposed to visualize ranking distributions) for **(A)** Number of patients with headache, **(B)** Number of deaths, and **(C)** Number of patients with fever.

Two RCTs reported the number of patients with headache, involving 188 participants (ΔDIC = 0.03, I^2^ = 17%). The overall heterogeneity was low, and a random-effects model was adopted. The NMA results indicated that, in comparison with SoC alone, dietary supplementation exerted no significant effect on the number of patients with headache. The details are shown in Supplementary Table S9. The forest plot is provided in Figure 3A. The SUCRA probability ranking results were as follows: SoC (82.52%) > melatonin+SoC (47.28%) > quercetin+SoC (20.19%). SoC alone was superior in lowering the number of patients with headache (Figure 4A).

##### 3.2.2.8 Number of deaths

Seven RCTs reported the number of deaths, with 458 patients covered (ΔDIC = 0.08, I^2^ = 0%). The overall heterogeneity was low, and a random-effects model was applied. The NMA results showed that, compared with SoC alone, dietary supplementation exerted no significant effect on the number of deaths. The results are detailed in Supplementary Table S10. The forest plot is presented in Figure 3B. The SUCRA probability ranking results showed: vitamin C + SoC (72.99%) > vitamin D + SoC (60.36%) > quercetin+SoC (44.86%). Vitamin C + SoC was the most effective in reducing the number of deaths (Figure 4B).

##### 3.2.2.9 Number of patients with fever

In total, 4 RCTs reported the number of patients with fever, involving 280 patients (ΔDIC = 0.02, I^2^ = 11%). The overall heterogeneity was low, and a random-effects model was adopted. The NMA results indicated that, in comparison with SoC alone, dietary supplementation exerted no significant effect on the number of patients with fever. The details are shown in Supplementary Table S11, and the forest plot is provided in Figure 3C. The SUCRA probability ranking results were as follows: vitamin D + SoC (82.14%) > curcumin+piperine+SoC (55.87%) > SoC (46.38%). Vitamin D + SoC was the optimal regimen for lowering the number of patients with fever (Figure 4C).

##### 3.2.2.10 Meta-regression

Given the heterogeneity among the included studies in terms of sample size, treatment duration, publication year, and disease severity, we conducted meta-regression analyses on the outcomes that reached statistical significance. For IL-6, lymphocyte count, and NLR, no significant associations were observed between these outcomes and any of the covariates (sample size, treatment duration, or disease severity).

### 3.3 Publication bias

Publication bias was assessed by generating correction-comparison funnel plots. The results indicated that all funnel plots were symmetrical, suggesting an absence of significant publication bias (Supplementary Figures S3, S4).

For the other secondary outcomes (cough and dyspnea), the results are detailed in Supplementary material 5.

## 4 Discussion

Compared with SoC alone, significant changes were observed in IL-6, NLR, and lymphocyte count among patients with COVID-19 following supplementation with nutritional or dietary supplements. However, no significant alterations were noted in WBC, CRP, neutrophil count, number of deaths, and number of patients with headache, cough, dyspnea, or fever.

### 4.1 IL-6

IL-6, a core pro-inflammatory cytokine in COVID-19, is closely associated with the abnormal activation of immune cells and dysregulated inflammatory pathways when overproduced. Vitamin C is known to function by specifically modulating key inflammatory mechanisms (16). The results of this study demonstrated that among patients with COVID-19 receiving dietary supplementation based on SoC, only vitamin C significantly reduced IL-6 levels. Xia et al. (16) summarizing data involving 222 severe COVID-19 patients, have confirmed that high-dosage intravenous vitamin C is significantly more effective than SoC alone in suppressing IL-6.

In terms of molecular pathway, vitamin C suppresses the activation of the NF-κB signaling pathway to block the transcription and secretion of IL-6 genes, which is an inhibitory effect that is dosage-dependent (17). The study by Fink et al. (18) notes that vitamin C inhibits the phosphorylation of inhibitory κB kinase induced by TNF-α, thereby blocking the activation of NF-κB from the upstream. This mechanism has been corroborated in inflammatory conditions such as sepsis and is also applicable to the inflammatory regulation in COVID-19.

Furthermore, the multi-dimensional mechanisms by which vitamin C modulates the pathophysiological process of COVID-19 are reasonably explained. Vitamin C significantly inhibits the excessive release of pro-inflammatory cytokines such as TNF-α, IL-1, IL-4, and IL-6 by downregulating the activity of the NF-κB pathway, thereby blocking the typical cytokine storm of severe COVID-19. Vitamin C reshapes the landscape of immune response through the JAK1/STAT1 pathway, promoting the activation and proliferation of CD4 + T cells, CD8 + T cells, B cells, and NK cells, and enhancing the migration ability of phagocytes and the secretion of IFN-α/*β*/*γ*. This process facilitates a shift from a Th2 to a Th1 response and the polarization of Th17 cells, thereby strengthening the antiviral immunity of the body. Particularly crucial is that vitamin C exerts an antiviral effect that directly targets SARS-CoV-2. Specifically, vitamin C reduces the expression of host cell ACE2, furin, and cathepsin L, blocking the cell adhesion and entry of viruses at the source. Vitamin C directly inactivates the key enzymes for virus replication (3CLPro and RdRp), inhibiting the transcription and translation of the virus genome, thereby halting the amplification of viruses in the body (19).

### 4.2 Lymphocyte count

Lymphocyte count is a core biomarker for assessing the immune function of cells among patients with COVID-19. A progressive decline in lymphocyte count is an independent risk factor for virus-induced immune exhaustion, which predicts disease progression to severe illness and death (20). SARS-CoV-2 directly infects CD4^+^ T cells and CD8^+^ T lymphocytes, thereby inducing lymphocyte damage and a reduction in number (21). The systemic inflammatory response secondary to viral infection further exacerbates lymphocyte apoptosis and immune function suppression, ultimately leading to a significant decrease in lymphocyte count in peripheral blood. Therefore, restoring adaptive immunity through exogenous intervention is considered a potential strategy to ameliorate patient prognosis. The results of this NMA showed that nanocurcumin combined with standard care significantly increased lymphocyte count in peripheral blood among COVID-19 patients (MD = 4.76, 95% CrI: 2.91–6.62), which is consistent with the findings of several published RCTs (22, 23).

Nevertheless, nanocurcumin intervention is not suitable for all COVID-19 patients, and inappropriate use may lead to adverse prognosis. From the perspective of immunopathological mechanisms, the disease course of COVID-19 exhibits dynamic changes characterized by immunosuppression - immune activation - cytokine storm (24). In the early stage of COVID-19 disease, patients predominantly present with lymphopenia and immunosuppression, and timely administration of nanocurcumin may help promote immune reconstitution. In the middle and late stages of COVID-19 disease, some patients are already in a state of immune hyperactivation, and blindly enhancing the immune response may disrupt the immune balance and aggravate inflammatory damage in the lungs and multiple organs throughout the body (24).

Based on the above immunopathological mechanisms and clinical research evidence, the application of nanocurcumin in COVID-19 patients needs to be assessed with caution, and appropriate populations should be strictly selected.

### 4.3 NLR

NLR is a comprehensive indicator reflecting the balance between innate immunity and adaptive immunity. An elevated NLR typically suggests excessive activation of inflammatory responses and immune dysregulation. Multiple meta-analyses have confirmed that an increased NLR at admission is a robust predictor for progression to severe illness and death among COVID-19 patients, with reported cutoff values ranging from 3.2 to 12.0 across studies. Moreover, dynamic changes in NLR more accurately reflect disease progression than a single time-point measurement (25).

The results from this study showed that pomegranate juice + SoC significantly increased NLR among COVID-19 patients (MD = 1.04, 95% CrI: 0.21–1.88), with a SUCRA probability as high as 90.16%. This finding differs from the conventional understanding that anti-inflammatory treatment should lower NLR. The mechanism can be reasonably explained by the temporal differences between innate and adaptive immune responses: pomegranate juice is rich in natural polyphenolic active components, which regulate systemic inflammation homeostasis and sequentially activate the immune response process in the body (26). However, other studies have shown that pomegranate juice + SoC reduces NLR among COVID-19 patients, which is consistent with the RCT by Yousefi et al. (27). That study has confirmed that after daily consumption of 500 mL of pomegranate juice for 14 days, NLR within the patient group was significantly lower than baseline (*p* < 0.001), suggesting that the natural polyphenolic components in pomegranate juice regulate systemic inflammation homeostasis and promote lymphocyte proliferation, thereby mitigating inflammation-related immune dysregulation. In summary, nutritional supplements are potentially effective as adjunctive treatment for COVID-19. Nevertheless, additional evidence of high quality is needed to support the clinical application of nutritional supplements. The above suggests the potential pitfalls and clinical limitations of pomegranate juice as an adjunctive treatment for COVID-19.

In conclusion, NLR among COVID-19 patients after pomegranate juice intervention may exhibit significant temporal fluctuations, and the specific pattern of its immunomodulatory effects remains to be fully elucidated. Even when pomegranate juice intervention is applied cautiously in a defined population, it is recommended to dynamically monitor immunity- and inflammation-related indicators throughout the whole treatment course. If abnormal fluctuations in these indicators occur, timely discontinuation or adjustment of the treatment regimen should be considered to reduce potential clinical risks.

### 4.4 Clinical significance and practical guidance

In clinical practice, for individuals suffering from COVID-19 with a tendency toward severe disease, indicated by elevated IL-6, supplementation with high-dosage intravenous vitamin C (100–200 mg·kg^−1^·d^−1^) added to SoC can serve as a targeted anti-inflammatory adjuvant regimen. This dosage can inhibit the activation of the NF-κB pathway in a dosage-dependent manner, blocking the cascading release of pro-inflammatory factors such as IL-6, thereby effectively preventing cytokine storms and significantly reducing the risk of severe disease progression and the incidence of multi-organ dysfunction (18).

For patients characterized predominantly by lymphopenia and immunosuppression, timely administration of nanocurcumin may help promote immune reconstitution. However, in the middle and late stages of the disease, some affected patients are already in a state of immune hyperactivation, and blindly enhancing the immune response may disrupt the immune balance and aggravate inflammatory damage in the lungs and multiple organs throughout the body (24).

NLR is a comprehensive indicator reflecting the balance between innate and adaptive immunity. An elevated NLR typically suggests excessive activation of inflammatory responses and immune dysregulation. The results from this study indicated that pomegranate juice + SoC significantly increased NLR among COVID-19 patients. Therefore, the application of pomegranate juice + SoC needs to be carefully assessed, and appropriate populations should be strictly selected along with dynamic monitoring of immunity- and inflammation-related indicators throughout an entire treatment course.

This study, through an extensive literature search, synthesized and analyzed the most comprehensive evidence to date regarding the impact of nutritional and dietary supplements on recovery and inflammatory responses among patients with COVID-19. The included studies covered multiple countries, enhancing the generalizability of the findings. However, certain limitations should be acknowledged. First, the number of included studies for some outcome indicators was relatively insufficient. Second, evidence loops were not formed for specific indicators within the NMA, increasing uncertainty in the comparison between direct and indirect evidence. These factors collectively moderated the credibility of the related findings.

In addition, network sparsity is a fundamental constraint. The evidence networks for most outcomes exhibited a star-shaped structure, without direct comparisons between interventions (nutritional supplements). Consequently, the ranking in this study relied on indirect comparisons, and it was difficult to distinguish true therapeutic advantages from biases within the included trials. A ranking based on SUCRA curves should be considered a hypothesis-generating reference rather than definitive evidence for selecting one supplement over another. Finally, the effect size of vitamin C on IL-6 (−88.77 pg./mL) exceeded a clinically reasonable range (28) (difference between severe and non-severe cases is approximately 20 pg./mL), and the estimate, based on indirect comparisons of only 268 patients, was extremely unstable. It should be regarded as an extreme point estimate under statistical fluctuation rather than interpreted as definitive efficacy.

Given these limitations, in the future, additional RCTs based on multicenter data and with a large sample size are warranted to target specific indicators such as IL-6, NLR, fever, and dyspnea to obtain more robust data. Moreover, additional RCTs are required for outcome indicators lacking evidence loops in the NMA to bridge evidence gaps and enhance the credibility of the analysis. It is suggested that other research directions be explored in the future. Nutritional supplements may exert limited effects on the recovery and inflammatory response of patients affected by COVID-19.

## 5 Conclusion

In conclusion, this study, through an NMA, systematically compares the relative efficacy of different dietary patterns and nutritional supplements on key outcomes among adult patients with COVID-19, aiming to identify the optimal regimen of nutrition intervention. Specifically, compared with SoC alone, vitamin C + SoC significantly lowers IL-6 levels; nanocurcumin+SoC significantly increases lymphocyte count; and pomegranate juice + SoC significantly elevates NLR levels. Although this study is affected by certain limitations, including an insufficient sample size for some outcomes, predominantly indirect evidence, and low confidence in some results, it is the first to systematically and horizontally quantify the immunomodulatory differences across an assortment of nutrients. This study distinguishes supplement regimens that confer biomarker improvements from those without clinical benefit, clarifies current contradictory evidence, and provides directions for precise stratified clinical trials in the future. Nevertheless, the findings in this study should serve only as preliminary references for adjunctive clinical nutrition support. It is recommended that in the future, RCTs based on multicenter data and with a large sample size should be warranted to strengthen the reliability of the relevant data.

## Data Availability

The original contributions presented in the study are included in the article/Supplementary material, further inquiries can be directed to the corresponding author.

## Footnotes

1
https://pubmed.ncbi.nlm.nih.gov/33782057/
