# Supplementary material for: Efficacy of supplementation with nutrients and dietary supplements on recovery and inflammatory response among patients with COVID-19: a network meta-analysis
Source: Front Nutr. 2026 Jul 28;13:1825539. doi: 10.3389/fnut.2026.1825539 (PMC13457122; doi:10.3389/fnut.2026.1825539)
Supplement: Supplementary file 1 [file Supplementary_file_1.PDF]

**Table S1. Search strategy (PubMed)**

| Search number | Query | Results |
|---|---|---|
| 1 | (COVID-19[MeSH Terms]) OR (SARS-CoV-2[MeSH Terms]) | 303640 |
| 2 | "2019 nCoV "[Title/Abstract] OR "2019 nCoV disease "[Title/Abstract] OR "2019 nCoV infection "[Title/Abstract] OR "2019 nCoV Disease "[Title/Abstract] OR "2019 nCoV Diseases "[Title/Abstract] OR "2019 nCoV Infection "[Title/Abstract] OR "2019 nCoV Infections "[Title/Abstract] OR "2019 new coronavirus "[Title/Abstract] OR "2019 novel coronavirus "[Title/Abstract] OR "2019 Novel Coronavirus Disease "[Title/Abstract] OR "2019 novel coronavirus epidemic "[Title/Abstract] OR "2019 Novel Coronavirus Infection "[Title/Abstract] OR "2019 Novel Coronaviruses "[Title/Abstract] OR "2019 severe acute respiratory syndrome coronavirus 2 "[Title/Abstract] OR "Coronavirus Disease 19 "[Title/Abstract] OR "coronavirus disease 2 "[Title/Abstract] OR "coronavirus disease 2010 "[Title/Abstract] OR "Coronavirus Disease 2019 "[Title/Abstract] OR "Coronavirus Disease 2019 Virus "[Title/Abstract] OR "coronavirus infection 2019 "[Title/Abstract] OR "coronavirus SARS 2 "[Title/Abstract] OR "COVID "[Title/Abstract] OR "COVID 10 "[Title/Abstract] OR "COVID 19 "[Title/Abstract] OR "COVID 19 virus "[Title/Abstract] OR "COVID 19 Pandemic "[Title/Abstract] OR "COVID 19 Pandemics "[Title/Abstract] OR "COVID 19 Virus "[Title/Abstract] OR "COVID 19 Virus Disease "[Title/Abstract] OR "COVID 19 Virus Diseases "[Title/Abstract] OR "COVID 19 Virus Infection "[Title/Abstract] OR "COVID 19 Virus Infections "[Title/Abstract] OR "COVID 2019 "[Title/Abstract] OR "COVID19 "[Title/Abstract] OR "COVID19 Virus "[Title/Abstract] OR "COVID19 Viruses "[Title/Abstract] OR "HCoV 19 "[Title/Abstract] OR "Human coronavirus 2019 "[Title/Abstract] OR "nCoV 2019 "[Title/Abstract] OR "nCoV 2019 disease "[Title/Abstract] OR "nCoV 2019 infection "[Title/Abstract] OR "novel 2019 coronavirus "[Title/Abstract] OR "novel coronavirus 19 "[Title/Abstract] OR "novel coronavirus 2019 "[Title/Abstract] OR "novel coronavirus 2019 disease "[Title/Abstract] OR "novel coronavirus 2019 infection "[Title/Abstract] OR "novel coronavirus disease 2019 "[Title/Abstract] OR "novel coronavirus infection 2019 "[Title/Abstract] OR "paucisymptomatic coronavirus disease 2019 "[Title/Abstract] OR "SARS 2 CoV "[Title/Abstract] OR "SARS 2 virus "[Title/Abstract] OR "SARS Coronavirus 2 "[Title/Abstract] OR "SARS Coronavirus 2 Infection "[Title/Abstract] OR "SARS coronavirus 2 nCoV SH01 "[Title/Abstract] OR "SARS CoV 2 "[Title/Abstract] OR "SARS CoV 2 disease "[Title/Abstract] OR "SARS CoV 2 nCoV SH01 "[Title/Abstract] OR "SARS CoV 2 Infection "[Title/Abstract] OR "SARS CoV 2 Virus "[Title/Abstract] OR "SARS CoV2 disease "[Title/Abstract] OR "SARS2 virus "[Title/Abstract] OR "SARSCoV2 disease "[Title/Abstract] OR "SARSCoV2 infection "[Title/Abstract] OR "SARS CoV2 infection | 465806 |

| | | |
|---|---|---|
| | "[Title/Abstract] OR "SARS CoV 2 infection "[Title/Abstract] OR "SARS CoV 2 Infection "[Title/Abstract] OR "SARS CoV 2 Infections "[Title/Abstract] OR "SARS CoV 2 Virus "[Title/Abstract] OR "SARS CoV 2 Viruses "[Title/Abstract] OR "SARS related coronavirus 2 "[Title/Abstract] OR "Sever acute respiratory syndrome coronavirus 2 "[Title/Abstract] OR "Severe acute respiratory coronavirus 2 "[Title/Abstract] OR "Severe acute respiratory syndorme coronavirus 2 "[Title/Abstract] OR "severe acute respiratory syndrome 2 "[Title/Abstract] OR "Severe acute respiratory syndrome 2 coronavirus "[Title/Abstract] OR "severe acute respiratory syndrome 2 virus "[Title/Abstract] OR "severe acute respiratory syndrome corona virus 2 "[Title/Abstract] OR "Severe Acute Respiratory Syndrome Coronavirus 2 "[Title/Abstract] OR "severe acute respiratory syndrome coronavirus 2 infection "[Title/Abstract] OR "Severe acute respiratory syndrome coronavirus 2 nCoV SH01 "[Title/Abstract] OR "Severe Acute Respiratory Syndrome Coronavirus 2 Infection "[Title/Abstract] OR "severe acute respiratory syndrome coronavirus 2019 "[Title/Abstract] OR "severe acute respiratory syndrome coronavirus 2019 infection "[Title/Abstract] OR "Severe acute respiratory syndrome coronoavirus 2 "[Title/Abstract] OR "Severe acute respiratory syndrome coronvirus 2 "[Title/Abstract] OR "severe acute respiratory syndrome CoV 2 infection "[Title/Abstract] OR "severe acute respiratory syndrome CoV 2 virus "[Title/Abstract] OR "Severe acute respiratory syndrome related coronavirus 2 "[Title/Abstract] OR "Severe acute respiratory syndrome virus 2 "[Title/Abstract] OR "Severe acute respiratoy syndrome coronavirus 2 "[Title/Abstract] OR "Wuhan Coronavirus "[Title/Abstract] OR "Wuhan coronavirus disease "[Title/Abstract] OR "Wuhan coronavirus infection "[Title/Abstract] OR "Wuhan Seafood Market Pneumonia Virus "[Title/Abstract] | |
| 3 | "randomised controlled study "[Title/Abstract] OR "randomised controlled trial "[Title/Abstract] OR "randomized controlled study "[Title/Abstract] OR "randomized controlled trial "[Title/Abstract] OR "random* "[Title/Abstract] OR "trial, randomized controlled "[Title/Abstract] OR "Clinical Trials, Randomized "[Title/Abstract] OR "Trials, Randomized Clinical "[Title/Abstract] | 1655048 |
| 4 | "adaflex "[Title/Abstract] OR "Anti Oxidant* "[Title/Abstract] OR "Antioxidant "[Title/Abstract] OR "Antioxidant Activity "[Title/Abstract] OR "antioxidant agent "[Title/Abstract] OR "Antioxidant Effect* "[Title/Abstract] OR "antioxidant nutrient "[Title/Abstract] OR "antioxidation agent "[Title/Abstract] OR "antioxidation product "[Title/Abstract] OR "antioxidative "[Title/Abstract] OR "Provinols "[Title/Abstract] OR "antoxidant "[Title/Abstract] OR "apl 510 "[Title/Abstract] OR "arginin "[Title/Abstract] OR "Arginine "[Title/Abstract] OR "arginine chlorhydrate "[Title/Abstract] OR "arginine chloride "[Title/Abstract] OR "arginine content "[Title/Abstract] OR "arginine hydrochloride "[Title/Abstract] OR "arginine monochloride | 1681530 |

"[Title/Abstract] OR "arginine monochlorohydrate "[Title/Abstract] OR "arginine monohydrochloride "[Title/Abstract] OR "arginine residue "[Title/Abstract] OR "Arginine Hydrochloride "[Title/Abstract] OR "arginyl residue "[Title/Abstract] OR "argivene "[Title/Abstract] OR "aritonin "[Title/Abstract] OR "bci 049 "[Title/Abstract] OR "betitol "[Title/Abstract] OR "bioarginina "[Title/Abstract] OR "celton xl "[Title/Abstract] OR "ceyestaeusom "[Title/Abstract] OR "ceyesto "[Title/Abstract] OR "Chiro Inositol "[Title/Abstract] OR "circadin "[Title/Abstract] OR "civasta "[Title/Abstract] OR "clemenos xl "[Title/Abstract] OR "Coenzyme* "[Title/Abstract] OR "cyclohexanehexol "[Title/Abstract] OR "cyclohexitol "[Title/Abstract] OR "dambose "[Title/Abstract] OR "davitamin "[Title/Abstract] OR "diet additive "[Title/Abstract] OR "diet supplement "[Title/Abstract] OR "Dietary Supplement* "[Title/Abstract] OR "Endogenous Antioxidant* "[Title/Abstract] OR "enteral nutrition product "[Title/Abstract] OR "Enzyme Cofactor* "[Title/Abstract] OR "eusom "[Title/Abstract] OR "Food Supplement* "[Title/Abstract] OR "Herbal Medicines "[Title/Abstract] OR "Herbal Supplement* "[Title/Abstract] OR "hexol "[Title/Abstract] OR "i inositol "[Title/Abstract] OR "inosil "[Title/Abstract] OR "inosimesol "[Title/Abstract] OR "inosit "[Title/Abstract] OR "inosite "[Title/Abstract] OR "inositene "[Title/Abstract] OR "inositina "[Title/Abstract] OR "Inositol "[Title/Abstract] OR "inositol medium "[Title/Abstract] OR "jan 13004 "[Title/Abstract] OR "ki 1001 "[Title/Abstract] OR "l arginine "[Title/Abstract] OR "l arginine chloride "[Title/Abstract] OR "l arginine hydrochloride "[Title/Abstract] OR "l arginine monochloride "[Title/Abstract] OR "l arginine monohydrochloride "[Title/Abstract] OR "L Arginine "[Title/Abstract] OR "lestinora "[Title/Abstract] OR "levo arginine* "[Title/Abstract] OR "m inositol "[Title/Abstract] OR "mallozen "[Title/Abstract] OR "meat sugar "[Title/Abstract] OR "mecastrin "[Title/Abstract] OR "melabiorytm "[Title/Abstract] OR "melatal "[Title/Abstract] OR "melatan "[Title/Abstract] OR "melatol "[Title/Abstract] OR "Melatonin "[Title/Abstract] OR "melatonina "[Title/Abstract] OR "melatonine "[Title/Abstract] OR "melatonite "[Title/Abstract] OR "mellaras "[Title/Abstract] OR "mellozzan "[Title/Abstract] OR "melovine "[Title/Abstract] OR "mesitol "[Title/Abstract] OR "meso inositol "[Title/Abstract] OR "mesoinosil "[Title/Abstract] OR "mesoinosite "[Title/Abstract] OR "mesoinositol "[Title/Abstract] OR "mesol "[Title/Abstract] OR "mesovil "[Title/Abstract] OR "mineral "[Title/Abstract] OR "minerals "[Title/Abstract] OR "mouse antialopecia factor "[Title/Abstract] OR "mucomel "[Title/Abstract] OR "muscle sugar "[Title/Abstract] OR "myo inositol "[Title/Abstract] OR "myoinosil "[Title/Abstract] OR "myoinositol "[Title/Abstract] OR "Neutraceutical* "[Title/Abstract] OR "noxarem "[Title/Abstract] OR "nucite "[Title/Abstract] OR "Nutraceutical* "[Title/Abstract] OR "Nutriceutical* "[Title/Abstract]

| | | |
|---|---|---|
| | OR "nutrition mixture "[Title/Abstract] OR "nutrition supplement* "[Title/Abstract] OR "nutrition support product "[Title/Abstract] OR "nutritional mixture "[Title/Abstract] OR "nutritional supplement "[Title/Abstract] OR "orlogin "[Title/Abstract] OR "parenteral nutrition product "[Title/Abstract] OR "phaseomannite "[Title/Abstract] OR "phaseomannitol "[Title/Abstract] OR "phenolic antioxidant "[Title/Abstract] OR "Plant Extract* "[Title/Abstract] OR "rat antispectacled eye factor "[Title/Abstract] OR "scyllite "[Title/Abstract] OR "sental "[Title/Abstract] OR "slenyto "[Title/Abstract] OR "sleplag "[Title/Abstract] OR "sloremina "[Title/Abstract] OR "sp 13004 "[Title/Abstract] OR "Supplement, Herbal "[Title/Abstract] OR "supplementary diet "[Title/Abstract] OR "syncrodin "[Title/Abstract] OR "tonozil "[Title/Abstract] OR "Vitamin* "[Title/Abstract] OR "waferest "[Title/Abstract] OR "Polyphenol* "[Title/Abstract] OR "diet*"[Title/Abstract] | |
| 5 | ((((((((Dietary Supplements[MeSH Terms]) OR (Coenzymes[MeSH Terms])) OR (Vitamins[MeSH Terms])) OR (Inositol[MeSH Terms])) OR (Arginine[MeSH Terms])) OR (minerals[MeSH Terms])) OR (Plant Extracts[MeSH Terms])) OR (Antioxidants[MeSH Terms])) OR (Melatonin[MeSH Terms])) OR (Polyphenols[MeSH Terms]) | 975995 |
| 6 | (#1 OR #2) AND #3 AND (#4 OR #5) | 1195 |

## Search strategy (Embase)

| Search number | Query | Results |
|---|---|---|
| 1 | '2019 ncov':ab,ti,kw OR '2019 ncov disease':ab,ti,kw OR '2019 ncov infection':ab,ti,kw OR '2019 ncov disease':ab,ti,kw OR '2019 ncov diseases':ab,ti,kw OR '2019 ncov infection':ab,ti,kw OR '2019 ncov infections':ab,ti,kw OR '2019 new coronavirus':ab,ti,kw OR '2019 novel coronavirus':ab,ti,kw OR '2019 novel coronavirus disease':ab,ti,kw OR '2019 novel coronavirus epidemic':ab,ti,kw OR '2019 novel coronavirus infection':ab,ti,kw OR '2019 novel coronaviruses':ab,ti,kw OR '2019 severe acute respiratory syndrome coronavirus 2':ab,ti,kw OR 'coronavirus disease 19':ab,ti,kw OR 'coronavirus disease 2':ab,ti,kw OR 'coronavirus disease 2010':ab,ti,kw OR 'coronavirus disease 2019':ab,ti,kw OR 'coronavirus disease 2019 virus':ab,ti,kw OR 'coronavirus infection 2019':ab,ti,kw OR 'coronavirus sars 2':ab,ti,kw OR 'covid':ab,ti,kw OR 'covid 10':ab,ti,kw OR 'covid 19':ab,ti,kw OR 'covid 19 virus':ab,ti,kw OR 'covid 19 pandemic':ab,ti,kw OR 'covid 19 pandemics':ab,ti,kw OR 'covid 19 virus':ab,ti,kw OR 'covid 19 virus disease':ab,ti,kw OR 'covid 19 virus diseases':ab,ti,kw OR 'covid 19 virus infection':ab,ti,kw OR 'covid 19 virus infections':ab,ti,kw OR 'covid 2019':ab,ti,kw OR 'covid19':ab,ti,kw OR 'covid19 virus':ab,ti,kw OR 'covid19 viruses':ab,ti,kw OR 'hcov 19':ab,ti,kw OR 'human coronavirus 2019':ab,ti,kw OR 'ncov 2019':ab,ti,kw OR 'ncov 2019 disease':ab,ti,kw OR 'ncov 2019 infection':ab,ti,kw OR 'novel 2019 coronavirus':ab,ti,kw OR 'novel coronavirus 19':ab,ti,kw OR 'novel coronavirus 2019':ab,ti,kw OR 'novel coronavirus 2019 disease':ab,ti,kw OR 'novel coronavirus 2019 infection':ab,ti,kw OR 'novel coronavirus disease 2019':ab,ti,kw OR 'novel coronavirus infection 2019':ab,ti,kw OR 'paucisymptomatic coronavirus disease 2019':ab,ti,kw OR 'sars 2 cov':ab,ti,kw OR 'sars 2 virus':ab,ti,kw OR 'sars coronavirus 2':ab,ti,kw OR 'sars coronavirus 2 infection':ab,ti,kw OR 'sars coronavirus 2 ncov sh01':ab,ti,kw OR 'sars cov 2':ab,ti,kw OR 'sars cov 2 disease':ab,ti,kw OR 'sars cov 2 ncov sh01':ab,ti,kw OR 'sars cov2 disease':ab,ti,kw OR 'sars2 virus':ab,ti,kw OR 'sarscov2 disease':ab,ti,kw OR 'sarscov2 infection':ab,ti,kw OR 'sars cov2 infection':ab,ti,kw OR 'sars cov 2 infection':ab,ti,kw OR 'sars cov 2 infection':ab,ti,kw OR 'sars cov 2 infections':ab,ti,kw OR 'sars cov 2 virus':ab,ti,kw OR 'sars cov 2 viruses':ab,ti,kw OR 'sars related coronavirus 2':ab,ti,kw OR 'sever acute respiratory syndrome coronavirus 2':ab,ti,kw OR 'severe acute respiratory coronavirus 2':ab,ti,kw OR 'severe acute respiratory syndorme coronavirus 2':ab,ti,kw OR 'severe acute respiratory syndrome 2':ab,ti,kw OR 'severe acute respiratory syndrome 2 coronavirus':ab,ti,kw OR 'severe acute respiratory syndrome 2 virus':ab,ti,kw OR 'severe acute respiratory syndrome corona virus 2':ab,ti,kw OR 'severe acute respiratory syndrome coronavirus 2':ab,ti,kw OR 'severe acute respiratory syndrome coronavirus 2 | 546675 |

| | | |
|---|---|---|
| | infection':ab,ti,kw OR 'severe acute respiratory syndrome coronavirus 2 ncov sh01':ab,ti,kw OR 'severe acute respiratory syndrome coronavirus 2 infection':ab,ti,kw OR 'severe acute respiratory syndrome coronavirus 2019':ab,ti,kw OR 'severe acute respiratory syndrome coronavirus 2019 infection':ab,ti,kw OR 'severe acute respiratory syndrome coronoavirus 2':ab,ti,kw OR 'severe acute respiratory syndrome coronvirus 2':ab,ti,kw OR 'severe acute respiratory syndrome cov 2 infection':ab,ti,kw OR 'severe acute respiratory syndrome cov 2 virus':ab,ti,kw OR 'severe acute respiratory syndrome related coronavirus 2':ab,ti,kw OR 'severe acute respiratory syndrome virus 2':ab,ti,kw OR 'severe acute respiratoy syndrome coronavirus 2':ab,ti,kw OR 'wuhan coronavirus':ab,ti,kw OR 'wuhan coronavirus disease':ab,ti,kw OR 'wuhan coronavirus infection':ab,ti,kw OR 'wuhan seafood market pneumonia virus':ab,ti,kw | |
| 2 | 'randomised controlled study':ab,ti,kw OR 'randomised controlled trial':ab,ti,kw OR 'randomized controlled study':ab,ti,kw OR 'randomized controlled trial':ab,ti,kw OR 'random*':ab,ti,kw OR 'trial, randomized controlled':ab,ti,kw OR 'clinical trials, randomized':ab,ti,kw OR 'trials, randomized clinical':ab,ti,kw | 2474086 |
| 3 | 'adaflex':ab,ti,kw OR 'anti oxidant*':ab,ti,kw OR 'antioxidant':ab,ti,kw OR 'antioxidant activity':ab,ti,kw OR 'antioxidant agent':ab,ti,kw OR 'antioxidant effect*':ab,ti,kw OR 'antioxidant nutrient':ab,ti,kw OR 'antioxidation agent':ab,ti,kw OR 'antioxidation product':ab,ti,kw OR 'antioxidative':ab,ti,kw OR 'provinols':ab,ti,kw OR 'antoxidant':ab,ti,kw OR 'apl 510':ab,ti,kw OR 'arginin':ab,ti,kw OR 'arginine':ab,ti,kw OR 'arginine chlorhydrate':ab,ti,kw OR 'arginine chloride':ab,ti,kw OR 'arginine content':ab,ti,kw OR 'arginine hydrochloride':ab,ti,kw OR 'arginine monochloride':ab,ti,kw OR 'arginine monochlorohydrate':ab,ti,kw OR 'arginine monohydrochloride':ab,ti,kw OR 'arginine residue':ab,ti,kw OR 'arginine hydrochloride':ab,ti,kw OR 'arginyl residue':ab,ti,kw OR 'argivene':ab,ti,kw OR 'aritonin':ab,ti,kw OR 'bci 049':ab,ti,kw OR 'betitol':ab,ti,kw OR 'bioarginina':ab,ti,kw OR 'celton xl':ab,ti,kw OR 'ceyestaeusom':ab,ti,kw OR 'ceyesto':ab,ti,kw OR 'chiro inositol':ab,ti,kw OR 'circadin':ab,ti,kw OR 'civasta':ab,ti,kw OR 'clemenos xl':ab,ti,kw OR 'coenzyme*':ab,ti,kw OR 'cyclohexanehexol':ab,ti,kw OR 'cyclohexitol':ab,ti,kw OR 'dambose':ab,ti,kw OR 'davitamin':ab,ti,kw OR 'diet additive':ab,ti,kw OR 'diet supplement':ab,ti,kw OR 'dietary supplement*':ab,ti,kw OR 'endogenous antioxidant*':ab,ti,kw OR 'enteral nutrition product':ab,ti,kw OR 'enzyme cofactor*':ab,ti,kw OR 'eusom':ab,ti,kw OR 'food supplement*':ab,ti,kw OR 'herbal medicines':ab,ti,kw OR 'herbal supplement*':ab,ti,kw OR 'hexol':ab,ti,kw OR 'i inositol':ab,ti,kw OR 'inosil':ab,ti,kw OR 'inosimesol':ab,ti,kw OR 'inosit':ab,ti,kw OR 'inosite':ab,ti,kw OR 'inositene':ab,ti,kw OR 'inositina':ab,ti,kw OR 'inositol':ab,ti,kw OR 'inositol medium':ab,ti,kw OR 'jan 13004':ab,ti,kw OR 'ki 1001':ab,ti,kw OR 'l arginine':ab,ti,kw OR 'l | 2160159 |

| | | |
|---|---|---|
| | arginine chloride':ab,ti,kw OR 'l arginine hydrochloride':ab,ti,kw OR 'l arginine monochloride':ab,ti,kw OR 'l arginine monohydrochloride':ab,ti,kw OR 'l arginine':ab,ti,kw OR 'lestinora':ab,ti,kw OR 'levo arginine*':ab,ti,kw OR 'm inositol':ab,ti,kw OR 'mallozen':ab,ti,kw OR 'meat sugar':ab,ti,kw OR 'mecastrin':ab,ti,kw OR 'melabiorytm':ab,ti,kw OR 'melatal':ab,ti,kw OR 'melatan':ab,ti,kw OR 'melatol':ab,ti,kw OR 'melatonin':ab,ti,kw OR 'melatonina':ab,ti,kw OR 'melatonine':ab,ti,kw OR 'melatonite':ab,ti,kw OR 'mellaras':ab,ti,kw OR 'mellozzan':ab,ti,kw OR 'melovine':ab,ti,kw OR 'mesitol':ab,ti,kw OR 'meso inositol':ab,ti,kw OR 'mesoinosil':ab,ti,kw OR 'mesoinosite':ab,ti,kw OR 'mesoinositol':ab,ti,kw OR 'mesol':ab,ti,kw OR 'mesovil':ab,ti,kw OR 'mineral':ab,ti,kw OR 'minerals':ab,ti,kw OR 'mouse antialopecia factor':ab,ti,kw OR 'mucomel':ab,ti,kw OR 'muscle sugar':ab,ti,kw OR 'myo inositol':ab,ti,kw OR 'myoinosil':ab,ti,kw OR 'myoinositol':ab,ti,kw OR 'neutraceutical*':ab,ti,kw OR 'noxarem':ab,ti,kw OR 'nucite':ab,ti,kw OR 'nutraceutical*':ab,ti,kw OR 'nutriceutical*':ab,ti,kw OR 'nutrition mixture':ab,ti,kw OR 'nutrition supplement*':ab,ti,kw OR 'nutrition support product':ab,ti,kw OR 'nutritional mixture':ab,ti,kw OR 'nutritional supplement':ab,ti,kw OR 'orlogin':ab,ti,kw OR 'parenteral nutrition product':ab,ti,kw OR 'phaseomannite':ab,ti,kw OR 'phaseomannitol':ab,ti,kw OR 'phenolic antioxidant':ab,ti,kw OR 'plant extract*':ab,ti,kw OR 'rat antispectacled eye factor':ab,ti,kw OR 'scyllite':ab,ti,kw OR 'sental':ab,ti,kw OR 'slenyto':ab,ti,kw OR 'sleplag':ab,ti,kw OR 'sloremina':ab,ti,kw OR 'sp 13004':ab,ti,kw OR 'supplement, herbal':ab,ti,kw OR 'supplementary diet':ab,ti,kw OR 'syncrodin':ab,ti,kw OR 'tonozil':ab,ti,kw OR 'vitamin*':ab,ti,kw OR 'waferest':ab,ti,kw OR 'polyphenol*':ab,ti,kw OR 'diet*':ab,ti,kw | |
| 4 | 'coronavirus disease 2019'/exp OR 'severe acute respiratory syndrome coronavirus 2'/exp | 503855 |
| 5 | 'randomized controlled trial'/exp | 1093677 |
| 6 | 'nutrition supplement'/exp OR 'dietary supplement'/exp OR 'coenzyme'/exp OR 'vitamin'/exp OR 'inositol'/exp OR 'arginine'/exp OR 'melatonin'/exp OR 'antioxidant'/exp OR 'plant extract'/exp OR 'mineral'/exp OR 'polyphenol'/exp | 1704494 |
| 7 | (#1 OR #4) AND (#2 OR #5) AND (#3 OR #6) | 2456 |

**Search strategy (Web of Science)**

| Search number | Query | Results |
|---|---|---|
| 1 | TS=((adaflex) OR (Anti Oxidant*) OR (Antioxidant) OR (Antioxidant Activity) OR (antioxidant agent) OR (Antioxidant Effect*) OR (antioxidant nutrient) OR (antioxidation agent) OR (antioxidation product) OR (antioxidative) OR (Provinols) OR (antoxidant) OR (apl 510) OR (arginin) OR (Arginine) OR (arginine chlorhydrate) OR (arginine chloride) OR (arginine content) OR (arginine hydrochloride) OR (arginine monochloride) OR (arginine monochlorohydrate) OR (arginine monohydrochloride) OR (arginine residue) OR (Arginine Hydrochloride) OR (arginyl residue) OR (argivene) OR (aritonin) OR (bci 049) OR (betitol) OR (bioarginina) OR (celton xl) OR (ceyestaeusom) OR (ceyesto) OR (Chiro Inositol) OR (circadin) OR (civasta) OR (clemenos xl) OR (Coenzyme*) OR (cyclohexanehexol) OR (cyclohexitol) OR (dambose) OR (davitamin) OR (diet additive) OR (diet supplement) OR (Dietary Supplement*) OR (Endogenous Antioxidant*) OR (enteral nutrition product) OR (Enzyme Cofactor*) OR (eusom) OR (Food Supplement*) OR (Herbal Medicines) OR (Herbal Supplement*) OR (hexol) OR (i inositol) OR (inosil) OR (inosimesol) OR (inosit) OR (inosite) OR (inositene) OR (inositina) OR (Inositol) OR (inositol medium) OR (jan 13004) OR (ki 1001) OR (l arginine) OR (l arginine chloride) OR (l arginine hydrochloride) OR (l arginine monochloride) OR (l arginine monohydrochloride) OR (L Arginine) OR (lestinora) OR (levo arginine*) OR (m inositol) OR (mallozen) OR (meat sugar) OR (mecastrin) OR (melabiorytm) OR (melatal) OR (melatan) OR (melatol) OR (Melatonin) OR (melatonina) OR (melatonine) OR (melatonite) OR (mellaras) OR (mellozzan) OR (melovine) OR (mesitol) OR (meso inositol) OR (mesoinosil) OR (mesoinosite) OR (mesoinositol) OR (mesol) OR (mesovil) OR (mineral) OR (minerals) OR (mouse antialopecia factor) OR (mucomel) OR (muscle sugar) OR (myo inositol) OR (myoinosil) OR (myoinositol) OR (Neutraceutical*) OR (noxarem) OR (nucite) OR (Nutraceutical*) OR (Nutriceutical*) OR (nutrition mixture) OR (nutrition supplement*) OR (nutrition support product) OR (nutritional mixture) OR (nutritional supplement) OR (orlogin) OR (parenteral nutrition product) OR (phaseomannite) OR (phaseomannitol) OR (phenolic antioxidant) OR (Plant Extract*) OR (rat antispectacled eye factor) OR (scyllite) OR (sental) OR (slenyto) OR (sleplag) OR (sloremina) OR (sp 13004) OR (Supplement, Herbal) OR (supplementary diet) OR (syncrodin) OR (tonozil) OR (Vitamin*) OR (waferest) OR (Polyphenol*) OR (diet*)) | 2885361 |
| 2 | TS=((2019 nCoV) OR (2019 nCoV disease) OR (2019 nCoV infection) OR (2019 nCoV Disease) OR (2019 nCoV Diseases) OR (2019 nCoV Infection) OR (2019 nCoV Infections) OR (2019 new coronavirus) OR (2019 novel | 650813 |

| | | |
|---|---|---|
| | coronavirus) OR (2019 Novel Coronavirus Disease) OR (2019 novel coronavirus epidemic) OR (2019 Novel Coronavirus Infection) OR (2019 Novel Coronaviruses) OR (2019 severe acute respiratory syndrome coronavirus 2) OR (Coronavirus Disease 19) OR (coronavirus disease 2) OR (coronavirus disease 2010) OR (Coronavirus Disease 2019) OR (Coronavirus Disease 2019 Virus) OR (coronavirus infection 2019) OR (coronavirus SARS 2) OR (COVID) OR (COVID 10) OR (COVID 19) OR (COVID 19 virus) OR (COVID 19 Pandemic) OR (COVID 19 Pandemics) OR (COVID 19 Virus) OR (COVID 19 Virus Disease) OR (COVID 19 Virus Diseases) OR (COVID 19 Virus Infection) OR (COVID 19 Virus Infections) OR (COVID 2019) OR (COVID19) OR (COVID19 Virus) OR (COVID19 Viruses) OR (HCoV 19) OR (Human coronavirus 2019) OR (nCoV 2019) OR (nCoV 2019 disease) OR (nCoV 2019 infection) OR (novel 2019 coronavirus) OR (novel coronavirus 19) OR (novel coronavirus 2019) OR (novel coronavirus 2019 disease) OR (novel coronavirus 2019 infection) OR (novel coronavirus disease 2019) OR (novel coronavirus infection 2019) OR (paucisymptomatic coronavirus disease 2019) OR (SARS 2 CoV) OR (SARS 2 virus) OR (SARS Coronavirus 2) OR (SARS Coronavirus 2 Infection) OR (SARS coronavirus 2 nCoV SH01) OR (SARS CoV 2) OR (SARS CoV 2 disease) OR (SARS CoV 2 nCoV SH01) OR (SARS CoV 2 Infection) OR (SARS CoV 2 Virus) OR (SARS CoV2 disease) OR (SARS2 virus) OR (SARSCoV2 disease) OR (SARSCoV2 infection) OR (SARS CoV2 infection) OR (SARS CoV 2 infection) OR (SARS CoV 2 Infection) OR (SARS CoV 2 Infections) OR (SARS CoV 2 Virus) OR (SARS CoV 2 Viruses) OR (SARS related coronavirus 2) OR (Sever acute respiratory syndrome coronavirus 2) OR (Severe acute respiratory coronavirus 2) OR (Severe acute respiratory syndorme coronavirus 2) OR (severe acute respiratory syndrome 2) OR (Severe acute respiratory syndrome 2 coronavirus) OR (severe acute respiratory syndrome 2 virus) OR (severe acute respiratory syndrome corona virus 2) OR (Severe Acute Respiratory Syndrome Coronavirus 2) OR (severe acute respiratory syndrome coronavirus 2 infection) OR (Severe acute respiratory syndrome coronavirus 2 nCoV SH01) OR (Severe Acute Respiratory Syndrome Coronavirus 2 Infection) OR (severe acute respiratory syndrome coronavirus 2019) OR (severe acute respiratory syndrome coronavirus 2019 infection) OR (Severe acute respiratory syndrome coronoavirus 2) OR (Severe acute respiratory syndrome coronvirus 2) OR (severe acute respiratory syndrome CoV 2 infection) OR (severe acute respiratory syndrome CoV 2 virus) OR (Severe acute respiratory syndrome related coronavirus 2) OR (Severe acute respiratory syndrome virus 2) OR (Severe acute respiratoy syndrome coronavirus 2) OR (Wuhan Coronavirus) OR (Wuhan coronavirus disease) OR (Wuhan coronavirus infection) OR (Wuhan Seafood Market Pneumonia Virus)) | |
| 3 | TS=((randomised controlled study) OR (randomised controlled trial) OR | 2713962 |

| | | |
|---|---|---|
| | (randomized controlled study) OR (randomized controlled trial) OR (random*) OR (trial, randomized controlled) OR (Clinical Trials, Randomized) OR (Trials, Randomized Clinical)) | |
| 4 | #3 AND #2 AND #1 | 1335 |

## Search strategy (Cochrane Library)

| Search number | Query | Results |
|---|---|---|
| 1 | MeSH descriptor: [COVID-19] explode all trees | 8688 |
| 2 | MeSH descriptor: [SARS-CoV-2] explode all trees | 3774 |
| 3 | ('adaflex ' OR 'Anti Oxidant* ' OR 'Antioxidant ' OR 'Antioxidant Activity ' OR 'antioxidant agent ' OR 'Antioxidant Effect* ' OR 'antioxidant nutrient ' OR 'antioxidation agent ' OR 'antioxidation product ' OR 'antioxidative ' OR 'Provinols ' OR 'antoxidant ' OR 'apl 510 ' OR 'arginin ' OR 'Arginine ' ,OR ,arginine chlorhydrate ' OR 'arginine chloride ' OR 'arginine content ' OR arginine hydrochloride ' OR 'arginine monochloride ' OR 'arginine monochlorohydrate ' OR 'arginine monohydrochloride ' OR 'arginine residue ' OR 'Arginine Hydrochloride ' OR 'arginyl residue ' OR 'argivene ' OR 'aritonin ' OR 'bci 049 ' OR 'betitol ' OR 'bioarginina ' OR 'celton xl ' OR 'ceyestaeusom ' OR 'ceyesto ' OR 'Chiro Inositol ' OR 'circadin ' OR 'civasta ' OR 'clemenos xl ' OR 'Coenzyme* ' OR 'cyclohexanehexol ' OR 'cyclohexitol ' OR 'dambose ' OR 'davitamin ' OR 'diet additive ' OR 'diet supplement ' , OR Dietary Supplement* ' OR 'Endogenous Antioxidant* ' OR 'enteral nutrition product ' OR 'Enzyme Cofactor* ' OR 'eusom ' OR 'Food Supplement* ' OR 'Herbal Medicines ' OR 'Herbal Supplement* ' OR 'hexol ' OR 'i inositol ' OR 'inosil ' OR 'inosimesol ' OR 'inosit ' OR 'inosite ' OR 'inositene ' OR 'inositina ' OR 'Inositol ' OR 'inositol medium ' OR 'jan 13004 ' OR 'ki 1001 ' OR 'l arginine ' OR 'l arginine chloride ' OR 'l arginine hydrochloride ' OR 'l arginine monochloride ' OR 'l arginine monohydrochloride ' OR 'L Arginine ' OR 'lestinora ' OR 'levo arginine* ' OR 'm inositol ' OR 'mallozen ' OR 'meat sugar ' OR 'mecastrin ' OR 'melabiorytm ' OR 'melatal ' OR 'melatan ' OR 'melatol ' OR 'Melatonin ' OR 'melatonina ' OR 'melatonine ' OR 'melatonite ' OR 'mellaras ' OR 'mellozzan ' OR 'melovine ' OR 'mesitol ' OR 'meso inositol ' OR 'mesoinosil ' OR 'mesoinosite ' OR 'mesoinositol ' OR 'mesol ' OR 'mesovil ' OR 'mineral ' OR 'minerals ' OR 'mouse antialopecia factor ' OR 'mucomel ' OR 'muscle sugar ' OR 'myo inositol ' OR 'myoinosil ' OR 'myoinositol ' OR 'Neutraceutical* ' OR 'noxarem ' OR 'nucite ' OR 'Nutraceutical* ' OR 'Nutriceutical* ' OR 'nutrition mixture ' OR 'nutrition supplement* ' OR 'nutrition support product ' OR 'nutritional mixture ' OR 'nutritional supplement ' OR 'orlogin ' OR 'parenteral nutrition product ' OR 'phaseomannite ' OR 'phaseomannitol ' OR 'phenolic antioxidant ' OR 'Plant Extract* ' OR 'rat antispectacled eye factor ' OR 'scyllite ' OR 'sental ' OR 'slenyto ' OR 'sleplag ' OR 'sloremina ' OR 'sp 13004 ' OR 'Supplement, Herbal ' OR 'supplementary diet ' OR 'syncrodin ' OR 'tonozil ' OR 'Vitamin* ' OR 'waferest ' OR 'Polyphenol* ' OR 'diet*'):ab,ti,kw | 192532 |
| 4 | ('randomised controlled study' OR 'randomised controlled trial' OR 'randomized controlled study' OR 'randomized controlled trial' OR 'random*' OR 'trial, randomized controlled' OR 'Clinical Trials, Randomized' OR | 1405326 |

| | | |
|---|---|---|
| | 'Trials, Randomized Clinical'):ab,ti,kw | |
| 5 | ('2019 nCoV' OR '2019 nCoV disease' OR '2019 nCoV infection' OR '2019 nCoV Disease' OR '2019 nCoV Diseases' OR '2019 nCoV Infection' OR '2019 nCoV Infections' OR '2019 new coronavirus' OR '2019 novel coronavirus' OR '2019 Novel Coronavirus Disease' OR '2019 novel coronavirus epidemic' OR '2019 Novel Coronavirus Infection' OR '2019 Novel Coronaviruses' OR '2019 severe acute respiratory syndrome coronavirus 2' OR 'Coronavirus Disease 19' OR 'coronavirus disease 2' OR 'coronavirus disease 2010' OR 'Coronavirus Disease 2019' OR 'Coronavirus Disease 2019 Virus' OR 'coronavirus infection 2019' OR 'coronavirus SARS 2' OR 'COVID' OR 'COVID 10' OR 'COVID 19' OR 'COVID 19 virus' OR 'COVID 19 Pandemic' OR 'COVID 19 Pandemics' OR 'COVID 19 Virus' OR 'COVID 19 Virus Disease' OR 'COVID 19 Virus Diseases' OR 'COVID 19 Virus Infection' OR 'COVID 19 Virus Infections' OR 'COVID 2019' OR 'COVID19' OR 'COVID19 Virus' OR 'COVID19 Viruses' OR 'HCoV 19' OR 'Human coronavirus 2019' OR 'nCoV 2019' OR 'nCoV 2019 disease' OR 'nCoV 2019 infection' OR 'novel 2019 coronavirus' OR 'novel coronavirus 19' OR 'novel coronavirus 2019' OR 'novel coronavirus 2019 disease' OR 'novel coronavirus 2019 infection' OR 'novel coronavirus disease 2019' OR 'novel coronavirus infection 2019' OR 'paucisymptomatic coronavirus disease 2019' OR 'SARS 2 CoV' OR 'SARS 2 virus' OR 'SARS Coronavirus 2' OR 'SARS Coronavirus 2 Infection' OR 'SARS coronavirus 2 nCoV SH01' OR 'SARS CoV 2' OR 'SARS CoV 2 disease' OR 'SARS CoV 2 nCoV SH01' OR 'SARS CoV 2 Infection' OR 'SARS CoV 2 Virus' OR 'SARS CoV2 disease' OR 'SARS2 virus' OR 'SARSCoV2 disease' OR 'SARSCoV2 infection' OR 'SARS CoV2 infection' OR 'SARS CoV 2 infection' OR 'SARS CoV 2 Infection' OR 'SARS CoV 2 Infections' OR 'SARS CoV 2 Virus' OR 'SARS CoV 2 Viruses' OR 'SARS related coronavirus 2' OR 'Sever acute respiratory syndrome coronavirus 2' OR 'Severe acute respiratory coronavirus 2' OR 'Severe acute respiratory syndorme coronavirus 2' OR 'severe acute respiratory syndrome 2' OR 'Severe acute respiratory syndrome 2 coronavirus' OR 'severe acute respiratory syndrome 2 virus' OR 'severe acute respiratory syndrome corona virus 2' OR 'Severe Acute Respiratory Syndrome Coronavirus 2' OR 'severe acute respiratory syndrome coronavirus 2 infection' OR 'Severe acute respiratory syndrome coronavirus 2 nCoV SH01' OR 'Severe Acute Respiratory Syndrome Coronavirus 2 Infection' OR 'severe acute respiratory syndrome coronavirus 2019' OR 'severe acute respiratory syndrome coronavirus 2019 infection' OR 'Severe acute respiratory syndrome coronoavirus 2' OR 'Severe acute respiratory syndrome coronvirus 2' OR 'severe acute respiratory syndrome CoV 2 infection' OR 'severe acute respiratory syndrome CoV 2 virus' OR 'Severe acute respiratory syndrome related coronavirus 2' OR 'Severe acute respiratory syndrome virus 2' OR 'Severe acute respiratoy syndrome coronavirus 2' OR 'Wuhan Coronavirus' OR 'Wuhan coronavirus disease' OR 'Wuhan coronavirus | 24693 |

| | | |
|---|---|---|
| | infection' OR 'Wuhan Seafood Market Pneumonia Virus'):ab,ti,kw | |
| 6 | MeSH descriptor: [Dietary Supplements] explode all trees | 19316 |
| 7 | MeSH descriptor: [Coenzymes] explode all tree | 4717 |
| 8 | MeSH descriptor: [Vitamins] explode all trees | 5705 |
| 9 | MeSH descriptor: [Inositol] explode all trees | 601 |
| 10 | MeSH descriptor: [Arginine] explode all trees | 1836 |
| 11 | MeSH descriptor: [Minerals] explode all trees | 4910 |
| 12 | MeSH descriptor: [Plant Extracts] explode all trees | 11038 |
| 13 | MeSH descriptor: [Antioxidants] explode all trees | 6346 |
| 14 | MeSH descriptor: [Melatonin] explode all trees | 1776 |
| 15 | MeSH descriptor: [Polyphenols] explode all trees | 1078 |
| 16 | (#1 OR #2 OR #5) AND #4 AND (#3 OR #6 OR #7 OR #8 OR #9 OR #10 | 1366 |
| | OR #11 OR #12 OR #13 OR #14 OR #15) | |
