# Supplementary material for: Efficacy of supplementation with nutrients and dietary supplements on recovery and inflammatory response among patients with COVID-19: a network meta-analysis
Source: Front Nutr. 2026 Jul 28;13:1825539. doi: 10.3389/fnut.2026.1825539 (PMC13457122; doi:10.3389/fnut.2026.1825539)
Supplement: Supplementary file 2 [file Supplementary_file_2.pdf]

Supplementary Material 2

Table S2: Characteristics of included studies

**First Author**	**Publication Year**	**Study Design**	**Country**	**Intervention**	**Sample Size**	**Age**	**Treatment Duration**		**Outcome Indicator**
Shabnam Tehrani	2022	RCT	Iran	vitamin C + SoC	18	58 (19)	5D	4	serum C-reactive protein (CRP) levels, lymphopenia, WBC, Platelet, Neutrophils, Oxygen saturation, Respiratory rate
				SoC	26	61 (17)			
Ikram Din Ujjan	2022	RCT	Pakistan	curcumin + quercetin + SoC	25	41.5 (13.7)	14D	2	Pyrexia, Cough, Myalgia, Pharyngitis, Flu, Asthenia, Dyspnea, Anosmia, Dysgeusia,
					25	37.1 (9.4)			
Mojtaba Yousef	2023	RCT	Iran	pomegranate juice + SoC	24	54.5 (14.0)	14D	4	IL-6, ESR, CRP, RBC, HB, HCT, MCV, MCH, MCHC, WBC,
				placebo + SoC	24	57.4 (15.7)			

									Lym,<br>Mono,<br>Neutrophils,<br>PLR,<br>NLR,<br>Plts
Hafiza Amna Benazir	2025	RCT	Pakistan	Vitamin C + SoC	60	58.2(14.3)	7D	5	Mortality (%),<br>ICU Length of Stay,<br>Mechanical Ventilation,<br>Improvement in PaO_2/FiO_2 Ratio,<br>CRP,<br>IL-6,<br>Serum Vitamin C
				SoC	60	59.5 (13.8)	7D		
Mikhail V. Bychinin	2022	RCT	Russia	Vitamin D + SoC	52	64.1463(10.6742)	7D	5	Mortality,<br>LOS ICU,<br>Hospital stay,<br>Mechanical ventilation requirement,<br>Mechanical ventilation,<br>Norepinephrine use,<br>Incidence of nosocomial infection,<br>Positive blood culture,<br>Lymphocyte,<br>NK cells CD3-CD56+CD16+,<br>NKT cells CD3+CD56+CD16+,<br>NLR, Interleukin-6,<br>CRP,<br>Procalcitonin,<br>25(OH)vitamin D,<br>White blood cell count,<br>Lymphocytes,<br>NK cells (CD3−CD56+CD16+),<br>NKT cells (CD3+CD56+CD16+),<br>NLR,<br>Neutrophils,
				Placebo+SoC	54	66.3289(20.5645)	7D		

									Platelets,<br>Creatinine,<br>Interleukin-6,<br>D-dimer Fibrinogen,<br>Procalcitonin,<br>CRP
Riccardo Calvani	2023	RCT	Italy	L-Arginine+VitaminC+SoC	23	47.3 (10.7)	28D	1	L-arginine plus vitamin C,<br>serum L-arginine levels,<br>L-arginine/ADMA ratio,<br>Total proteins,<br>Uric acid, Creatinine,<br>CRP,<br>Cortisol,<br>25-0H vitamin D,<br>LDH
				Placebo+SoC	23	48.4 (8.0)	28D		
Francesco Di Pierro	2021	RCT	Pakistan	Quercetin+SoC	21	42.5(3.3）	14D	1	Hospital discharge time (day),<br>mortality,<br>virus clearance,<br>symptoms frequency,<br>LDH,<br>ferritin, RT-PCR (positive subjects),<br>Symptoms variation,<br>CRP,<br>D-dimer,<br>E-Hospitalized patients,<br>F-Patients in the ICU,<br>G-Deaths,<br>H-oxygen saturation
				SoC	21	56.2 (3.3)	14D		
Francesco Di Pierro	2023	RCT	Pakistan	Quercetin+SoC	50	41.1(2.03)	14D	2	LDH,<br>CRP,<br>Ferritin,<br>D-Dimer,<br>E-oxygen saturation
				SoC	50	54.1(2.03)	14D		
Pitchay	2024	RCT	Thailand	Vitamin D +	147	47.90(16.7	14D	2	pneumonia treatment duration,

a Dilokpattanamongkol				SoC		7)			length of hospital stay, Serum calcium, Serum phosphate, Serum 25OHD, Vitamin D deficiency and change in pneumonia severity index between enrollment and discharge, Subgroup analysis was conducted for supplemental oxygen use, high-dose corticosteroid administration, evidence of lymphopenia,
				SoC	147	53.71(18.80)	14D		
Josipa Domazet Bugarin	2023	RCT	Croatia	Vitamin D + SoC	75	65(9.0706)	14D	5	Number of days on respirator, Number of days in the ICU, Number of days in hospital, WHO clinical progression scale on admission, WHO clinical progression scale on day 28, Survival at day 14 (%), Survival at day 28 (%), Survival at day 60 (%), Number of bacterial superinfections (n %), PaO_2/FiO_2 ratio, D-dimer levels, fibrinogen, ferritin, PCT,
				SoC	77	61.9747(32.4865)	14D		
Yasmine M. Elamir	2022	RCT	America	Vitamin D + SoC	25	64 ±16	14D	4	fraction of oxygen (SaO_2/FIO_2 ratio), Length of hospital stay (days), ICU admission, Endotracheal intubation, Mortality, Readmission within 30 days
				SoC	25	69 ±18	14D		
Fabienne Jaun	2023	RCT	Switzerland	SoC + vitamin D3	39	60.49（13.84）	16D	4	LOS, 25(OH)D3, ICU admission, mechanical ventilation, death,
				SoC+placebo	39	61.38	15D		

						（15.29）			and other complications linked to COVID-19. Additionally, we looked at the serum 25(OH)D3 levels, the health-related quality of life O2 -supplementation on days 28 and 90, self-reported symptoms, and vital signs. For safety outcomes, the occurrence of adverse events (AEs) and serious adverse events (SAEs) were assessed
Tatiana L. Karonova	2024	RCT	Russia	SoC+ water-soluble cholecalciferol	22	60.402(8.9551)	9D	4	25(OH)D, cytokine
				SoC	22	63.722(10.8571)	9D		
Vijay Kuma	2022	RCT	India	vitamin C + SoC	30	57(12.8)	4D	4	n-hospital mortality
				SoC	30	63.3(16.3)	4D		
Poona Kumari	2020	RCT	Karachi	vitamin C + SoC	75	52(11)	7D	5	The number of days required for the disappearance of symptoms, number of days spent in the hospital, Overall death,
				SoC	75	53(12)	8D		
Zohre Labbani-Motlagh	2022	RCT	Iran	Vitamin C+ SoC	37	57.84(14.722)	4D	4	SOFA score
				SoC	37	58.89(14.46)	4D		
Nazanin Majidi	2024	RCT	Iran	SoC+ vitamin B complex	31	59.42(15.07)	14D	5	higher mean survival, level of serum K
				SoC	69	63.82(14.58)	14D		
G. Farnoo	2022	RCT	Iran	Melatonin+SoC	24	50.75（14.43）	14D	2	Signs and symptoms: fever, cough, dyspnea, myalgia, fatigue, chill, headache, GI symptoms, sputum

sh, M				SoC	20	52.95 （14.07）	14D		production, sore throat, chest pain, rhinorrhea<br>Laboratory findings (NLR, median (IQR), ESR, median (IQR), mm/h, Positive CRP ≥10 mg/L, Pulmonary involvement)<br>Clinical outcome (Hospital discharge, Time to discharge, M±SD, day, Return to baseline health, M±SD, day, Hospital stays, ICU admission, Death)
M. Ganjifard	2025	RCT	Iran	melatonin+SOC	23	62.5(18.6)	14D	5	AST<br>ALT<br>LDH<br>ESR<br>CRP<br>PaO_2/FiO_2 ratio<br>Lymphocyte count
				SoC	23	52.8(16.1)	14D		
A. Hakamifard	2022	RCT	Iran	Vitamin C + Vitamin E + SoC	38	35.68	The above-mentioned drugs were administered to both groups during their hospitalization until discharge or admission to the ICU	3	Vital signs (T, PR, RR, WBC, Neut Lymph, Plt, O_2, LDH)
				SoC	34	37.41	The above-mentioned drugs were administered to both		

							groups during their hospitalization until discharge or admission to the ICU.		
Z. T. Hasan	2022	RCT	Iraq	Melatonin+SoC	82	56.8 (7.5)	Throughout the entire hospitalization period	5	CRP, Serum Ferritin and D-Dimer
				SoC	76	55.7 (8.0)	Throughout the entire hospitalization period		
Q. Hu	2022	RCT	China	Nicotinamide + SoC	12	69 (12)	2D	4	lymphocyte counts, full blood counts
				SoC	12	70 (12)	2D		
N. Jaiswal	2023	RCT	India	N-Acetyl Cysteine + SoC	50	35.0 (13.0)	10D	2	Negative conversion rate of R-PCR within 5 to 7 days Adverse effects (Breathing difficulty, Anxiety, Nausea/vomiting, G.I upsets, Headache, Skin rashes)
				SoC	50	38.6 (14.2)	10D		
				SoC	259	39.27 (15.28)	14 D		
S. Ahmadi	2024	RCT	Iran	Nanocurcumin+ SoC	29	54.69 (13.44)	14 D	2	The acute phase proteins: IL-6, CRP, ALB, Erythrocyte, ESR. Biochemical markers: LDH, AST, ALT, CBC
				Placebo+SoC	39	53.94 (11.62)	14 D		
H. R. Bahrami-Taghanaki	2024	RCT	Iran	Phytopaj+SoC	44	61.2 (15.7)	14 D	4	peripheral blood lymphocyte count (LC) and blood oxygen saturation (PO_2)
				SoC	49	52.7 (14.0)	14 D		
A.	2024	RCT	Iran	ginger+SoC	36	36.38	7 D	1	SARS-CoV-2 clearance, SpO_2, Temperature, Respiratory

Ameri						(13.19)			rate, Hospital admission
				Placebo+SoC	42	34.57 (10.62)	7 D		
A. Ameri	2023	RCT	Iran	Melatonin+SoC	109	54.60 (11.51)	7 D	5	In-hospital mortality until day 28, Invasive mechanical ventilation, Clinical status at day 7a, Clinical status at day 14a, Time to clinical improvement, LOS in hospital until discharge, LOS in ICU, Ventilator-free days
				SoC	117	54.69(13.40)	7 D		
G. Askari	2022	RCT	Iran	curcumin+piperine+SoC	23	43.74 (12.9)	14 D	2	The improvement of clinical symptoms, CBC, FBS, serum cholesterol, TG, LDL, HDL, VLDL, ALT, AST, LDH, creatinine, BUN, CRP
				Placebo+SoC	23	51.52 (13.8)	14 D		
S. Ahmadi	2023	RCT	Iran	Nano-curcumin +SoC	29	54.6 (13.4)	14 D	2	Clinical symptoms: cough, muscle pain, sore throat, diarrhea, nausea,<br>vomiting, change in the sense of taste and smell, fatigue, chills,<br>confusion, feeling pain or pressure in the chest, number of<br>breaths per minute, need for oxygen mask, and SPO_2
				Placebo+SoC	39	53.9 (11.9)	14 D		
N. Alizadeh	2022	RCT	Iran	Melatonin+SoC	34	61.27 (18.09)	5 D	5	mortality rate, duration of mechanical ventilation, changes in oxygenation indices, and CRP levels. GCS status, hemodynamic and laboratory parameters, oxygenation indices, and prescribed medicines. the length of ICU stay, duration of mechanical ventilation, extubation rate, mortality, changes in oxygenation indices, and laboratory tests (i.e., WBC, CRP, ALT, AST, and Cr)
				Placebo+SoC	33	65.35 (19.30)	5 D		
R. Ahmadi	2021	RCT	Iran	Nano-curcumin +SoC	30	41.33 (12.04)	14 D	2	Signs and symptoms, laboratory data (CRP serum level and lymphocytes count), the time of each symptom resolution
				Placebo+SoC	30	44.97 (11.00)	14 D		
Z. Alizadeh	2021	RCT	Iran	Melatonin+SoC	14	37.57(8.2)	14 D	2	CRP 1 (first time) and oxygen saturation. CRP 2 (after 14 days) measurement.
				SoC	17	34.53(8.2)	14 D		

S. B. Abdallah	2023	RCT	Tunisia	Zinc+SoC	231	54.6 (17.4)	15 D	3	Death, ICU admission
				Placebo+SoC	239	53.7 (17.2)	15 D		
I. Angeles-Agdeppa	2024	RCT	Philippines	Virgin coconut oil + SoC	38	43.5 (15.9)	28 D	2	Signs and symptoms alleviation, fasting blood sugar (FBS), lipid profile, liver function test (AST/ALT), WBC count, WBC differential count, and CRP
				SoC	38	42.9 (15.1)	28 D		
Rodrigo Fernandes Weyll Pimentel	2022	RCT	Brazil	L-arginine+Nucleotide+Fatty acid+SoC	21	41.1 (2.8)	7 D	3	1. total lymphocyte count, serum CRP level<br>2. Nutritional risk (15): The Nutritional Risk Screening 2002 (NRS 2002) tool (NRS 2002)
				Placebo+SoC	22	41.9 (2.6)	7 D		
Ashu Rastogi	2022	RCT	India	Vitamin D + SoC	16	45.2916(12.1923)	7 D+14 D(仍 25(OH)D <50 ng/ml patients)	1	1. Biochemical parameters: 25 (OH) D3, C-reactive protein, Calcium, Phosphorus<br>2. The levels of serum inflammatory markers: CRP(ng/ml): Fibrinogen , D-dimer, Vitamin D, Procalcitonin<br>3. Proportion of patients with SARSCoV-2 RNA negative before day-21:SARS-CoV-2 RNA detection at day-5, 7, 10, 14, 18, and 21
				Placebo+SoC	24	45.1722(7.8018)	7 D+14 D(仍 25(OH)D <50 ng/ml patients)		
Diana Rodríguez-Vera	2024	RCT	Mexico	Fatty Acid +SoC	17	48.8 (19.1)	14 D	3	neutrophils,<br>lymphocytes,<br>monocytes,<br>hematocrit levels,<br>D-dimer,<br>LDH,<br>Procalcitonin,<br>HDL,<br>LDL/HDL ratios,
				Placebo+SoC	23	52.1(22.9)	14 D		

									VLDL,<br>LDL,<br>glucose,<br>creatinine, GPT,<br>GOT,<br>direct and indirect bilirubin
Arash Rahimi	2023	RCT	Iran	Acetylcysteine+ SoC	20	58.75(15.91)	14 D	5	D-dimer,<br>LDH,<br>Lymphocyte count, Ferritin, CRP,<br>Duration of hospitalization in the ward or in the ICU,<br>Mortality, respiratory rate,<br>changes in diastolic blood pressure, SpO_2(%),<br>Mechanical ventilation, Intubation,<br>Oxygen with a mask,<br>Nasal oxygen
				Placebo+SoC	20	58.30(15.56)	14 D		
Seyed Abbas Mousavi	2022	RCT	Iran	Melatonin+SoC	48	51.06 (15.86)	7 D or until death	3	white blood cell count,<br>lymphocyte count,<br>CRP status, intensive care unit admission, mortality,<br>peripheral capillary oxygen saturation,<br>symptoms alleviation,<br>Symptoms duration (day),<br>Leeds Sleep Evaluation Questionnaire (LSEQ).
				SoC	48	54.77 (15.34)	7 D or until death		
George Pavlidis	2025	RCT	Greece	Glycocalyx dietary supplement (GDS) + SoC	29	55(9)	120 D	1	hs- CRP,<br>eGFR,<br>Platelets,<br>White blood cells,<br>Neutrophils,<br>Lymphocytes, Monocytes,<br>Neutrophil- to- lymphocyte ratio,<br>Monocyte- to- lymphocyte ratio,<br>Platelet- to- lymphocyte ratio,<br>changes in arterial stiffness,<br>coronary microcirculation,
				Placebo+SoC	28	56(8)	120 D		
R.L.	2024	RCT	Argentin	Vitamin D +	11	59.8 (10.7)	10 D	1	IL-6,

Sanz			a	SoC					IL-8,<br>IL-10,<br>TNF-α,<br>25-OH Vitamin D,<br>Ang II, Ang-(1---7),<br>trimethylamine (TMA),<br>trimethylamine-N-oxide (TMAO),<br>ICU length of stay,<br>ICU admission, In-hospital death,<br>Systolic and diastolic blood pressure,<br>Mechanical ventilation
				Placebo+SoC	11	58.3 (10.6)	10 D		
Margot P. J. Visser	2024	RCT	The Netherlands	Vitamin K + SoC	20	59.8 (10.4)	discharge or for a maximum of 14 d	4	Lymphocytes,<br>CRP,<br>LDH,<br>Creatinine,<br>Dp-ucMGP,<br>PIVKA-II,<br>Desmosine,<br>IL-6,<br>D-dimer
				Placebo+SoC	20	56.2 (8.3)	Discharge or for a maximum of 14 d		
Mohammad Hosein Zamanian	2024	RCT	Iran	Vitamin D + SoC	22	56.33 (14.6)		5	25-Hydroxyvitamin D,<br>Alb,<br>Total calcium,<br>Creatinine,<br>BUN,<br>In-hospital mortality,<br>Mechanical ventilation requirement
				Placebo+SoC	27	50.65 (11.4)			
Jing Zhang	2021	RCT	China	Vitamin C + SoC	27	66.3 (11.2)	7d	5	Leukocyte count,<br>Neutrophil count,<br>Neutrophil ratio,<br>IL-6,<br>Lymphocyte count,<br>Lymphocyte ratio,<br>PCT,
				Placebo+SoC	29	67.0 (14.3)	7d		

									CRP,<br>Total bilirubin,<br>Creatinine,<br>BUN,<br>PT,<br>28-day mortality,<br>organ failure,<br>invasive mechanical ventilation-free days in 28 days
Mohsen Sedighiyan	2021	RCT	Iran	Fatty Acid+SoC	15	66.46(2.89)	14 D	3	C-- reactive protein (CRP),<br>erythrocyte sedimentation rate (ESR)，<br>liver enzymes,<br>body pain,<br>fatigue,<br>appetite and olfactory,<br>self - reported questionnaires,<br>Clinical sign scores of participants
				SoC	15	67.06(2.28)	14 D		

Table S2: Deviance information criterion and heterogeneity of different models for each outcome indicator

**Outcome Indicator**	Dbar	pD	DIC		ΔDIC	I^2	
			Consistency	Ume		Consistency	Ume
IL-6	12.35	8.99	21.34	21.43	0.09	11%	11%
headache	6.00	5.50	11.51	11.54	0.03	17%	17%
cough	14.55	13.70	28.26	28.20	0.06	11%	10%
fever	10.09	8.65	18.74	18.72	0.02	11%	11%
lymphocyte count	15.04	8.00	23.04	23.02	0.02	34%	33%
NLR	5.46	5.00	10.46	10.45	0.01	8%	8%
dyspnea	6.12	5.93	12.05	11.94	0.11	18%	18%
CRP	47.69	40.43	88.11	88.20	0.09	10%	10%
WBC	11.68	11.01	22.70	22.56	0.14	14%	14%
number of deaths	12.84	12.24	25.08	25.00	0.08	0%	0%
neutrophil count	6.88	5.41	12.29	12.25	0.04	27%	27%

Table S3: League table for the comparison of the effects of different interventions versus SoC on IL-6 level

**SoC**			
0.47 (-0.73, 1.68)	**Vitamin D + SoC**		
**-88.77 (-139.02, -38.66)**	**-89.22 (-139.48, -39.11)**	**Vitamin C + SoC**	
**-6.11 (-10.69, -1.51)**	**-6.57 (-11.32, -1.82)**	**82.67 (32.33, 133.16)**	**Pomegranate juice + SoC**

The data in the table are the MD (95% credible interval) of the intervention in the row compared with the intervention in the column. A larger MD represents a higher IL-6; 0 represents no difference between the two interventions. Bolding represents a statistically significant decrease in IL-6.

Table S4: League table for the comparison of the effects of different interventions versus SoC on lymphocyte count

**SoC**								
0.08 (-0.44, 0.6)	**Glycocalyx+SoC**							
-0.41 (-1.03, 0.21)	-0.49 (-1.3, 0.32)	**Acetylcysteine+SoC**						
**4.76 (3.74, 5.78)**	**4.68 (3.54, 5.83)**	**5.18 (3.98, 6.36)**	**Nanocurcumin+SoC**					
0.01 (-0.27, 0.28)	-0.07 (-0.66, 0.51)	0.42 (-0.26, 1.1)	-4.76 (-5.81, -3.7)	**Melatonin+SoC**				
-0.43 (-1, 0.14)	-0.51 (-1.28, 0.26)	-0.02 (-0.86, 0.83)	-5.19 (-6.36, -4.03)	-0.44 (-1.07, 0.2)	**Vitamin D + SoC**			
-0.17 (-0.57, 0.23)	-0.25 (-0.91, 0.4)	0.24 (-0.5, 0.97)	-4.94 (-6.03, -3.84)	-0.18 (-0.67, 0.3)	0.25 (-0.44, 0.95)	**Vitamin C + SoC**		
0.08 (-0.35, 0.5)	0 (-0.68, 0.67)	0.49 (-0.27, 1.24)	-4.69 (-5.79, -3.58)	0.07 (-0.44, 0.58)	0.5 (-0.2, 1.22)	0.25 (-0.33, 0.84)	**Vitamin B + SoC**	
**1.75 (1.03, 2.45)**	**1.66 (0.78, 2.55)**	**2.16 (1.21, 3.1)**	-3.02 (-4.26, -1.77)	**1.74 (0.98, 2.5)**	**2.17 (1.26, 3.08)**	**1.92 (1.11, 2.73)**	**1.67 (0.84, 2.5)**	**L+N+F+SoC**

The data in the table are the SMD (95% credible interval) of the intervention in the row compared with the intervention in the column. A larger SMD represents a higher lymphocyte Count; 0 represents no difference between the two interventions. Bolding represents a statistically significant increase in lymphocyte count.

Table S5: League table for the comparison of the effects of different interventions versus SoC on NLR

**SoC**					
-0.14 (-0.66, 0.38)	**Glycocalyx+ SoC**				
0.09 (-0.28,	0.23 (-0.41,	**Melatonin+**			

0.47)	0.87)	**SoC**			
0.43 (-0.13, 1)	0.57 (-0.2, 1.34)	0.34 (-0.34, 1.02)	**Vitamin D + SoC**		
-0.41 (-0.87, 0.06)	-0.27 (-0.96, 0.43)	-0.5 (-1.09, 0.1)	**-0.84 (-1.57, -0.11)**	**Vitamin C + SoC**	
-0.61 (-1.22, 0)	-0.47 (-1.27, 0.34)	-0.7 (-1.41, 0.02)	**-1.04 (-1.88, -0.21)**	-0.2 (-0.97, 0.57)	**Pomegranate juice + SoC**

The data in the table are the MD (95% credible interval) of the intervention in the row compared with the intervention in the column. A larger MD represents a higher NLR; 0 represents no difference between the two interventions. Bolding represents a statistically significant decrease in NLR.

Table S6: League table for the comparison of the effects of different interventions versus SoC on WBC count

**SoC**										
0.23 (-1.01, 1.46)	**Glycocalyx+SoC**									
-0.33 (-1.61, 0.95)	-0.56 (-2.34, 1.22)	**Acetylcysteine+SoC**								
-0.03 (-1.24, 1.2)	-0.25 (-1.99, 1.49)	0.31 (-1.46, 2.08)	**Nanocurcumin+SoC**							
0.04 (-1.18, 1.25)	-0.19 (-1.92, 1.55)	0.37 (-1.39, 2.13)	0.07 (-1.67, 1.79)	**Ginger+SoC**						
0.55 (-0.69, 1.76)	0.32 (-1.42, 2.06)	0.88 (-0.9, 2.65)	0.57 (-1.17, 2.29)	0.51 (-1.22, 2.23)	**Vitamin C +Vitamin E + SoC**					
0 (-1.22, 1.23)	-0.23 (-1.97, 1.51)	0.33 (-1.44, 2.09)	0.02 (-1.72, 1.76)	-0.05 (-1.76, 1.69)	-0.55 (-2.27, 1.19)	**Melatonin+SoC**				
-0.08 (-0.99, 0.79)	-0.31 (-1.86, 1.2)	0.25 (-1.33, 1.78)	-0.05 (-1.58, 1.43)	-0.12 (-1.63, 1.37)	-0.63 (-2.15, 0.88)	-0.07 (-1.62, 1.42)	**Vitamin C + SoC**			
0.35 (-0.85, 1.56)	0.12 (-1.6, 1.85)	0.68 (-1.06, 2.44)	0.37 (-1.34, 2.09)	0.31 (-1.38, 2.02)	-0.2 (-1.91, 1.54)	0.35 (-1.37, 2.07)	0.43 (-1.06, 1.94)	**Vitamin B + SoC**		

-0.82 (-2.09, 0.45)	-1.05 (-2.82, 0.73)	-0.49 (-2.29, 1.32)	-0.8 (-2.56, 0.97)	-0.86 (-2.62, 0.91)	-1.36 (-3.12, 0.4)	-0.82 (-2.58, 0.95)	-0.74 (-2.28, 0.83)	-1.17 (-2.92, 0.58)	**Pomegranate juice + SoC**	
-0.53 (-1.79, 0.72)	-0.76 (-2.52, 1)	-0.2 (-1.98, 1.59)	-0.51 (-2.26, 1.24)	-0.57 (-2.32, 1.17)	-1.08 (-2.83, 0.68)	-0.53 (-2.29, 1.22)	-0.45 (-1.97, 1.1)	-0.88 (-2.63, 0.85)	0.29 (-1.51, 2.07)	**Curcumin+Piperine +SoC**

The data in the table are the SMD (95% credible interval) of the intervention in the row compared with the intervention in the column. A larger SMD represents a higher WBC Count; 0 represents no difference between the two interventions. Bolding represents a statistically significant decrease in WBC.

Table S7: League table for the comparison of the effects of different interventions versus SoC on neutrophil count

**SoC**				
0.1(-0.83,1.02)	**Glycocalyx+SoC**			
-0.3(-1.27,0.68)	-0.39(-1.74,0.96)	**Acetylcysteine+SoC**		
0.32(-0.59,1.23)	0.22(-1.07,1.52)	0.62 (-0.72,1.94)	**Melatonin+SoC**	
0.01(-0.55,0.52)	-0.09(-1.18,0.96)	0.3 (-0.83, .4)	-0.31(-1.38,0.72)	**Vitamin C + SoC**

The data in the table are the SMD (95% credible interval) of the intervention in the row compared with the intervention in the column. A larger SMD represents a higher Neutrophils Count; 0 represents no difference between the two interventions. Bolding represents a statistically significant decrease in Neutrophils Count.

Table S8: League table for the comparison of the effects of different interventions versus SoC on CRP

**SoC**														
-20.89 (-44.07, 2.11)	**Fatty acid + SoC**													
-12.42 (-42.81, 18.03)	8.5 (-29.4, 46.51)	**Acetyl cystein e+SoC**												
-11.98 (-44.55, 20.56)	8.96 (-30.82 , 48.37)	0.52 (-44.07 , 44.47)	**Nanoc urcumi n+SoC**											
1.92 (-22.46, 25.91)	22.85 (-10.74 , 56.22)	14.35 (-24.6, 52.72)	13.87 (-26.67 , 54.21)	**Virgin coconut oil + SoC**										
0.38 (-22.68, 23.39)	21.4 (-11.59 , 53.78)	12.87 (-25.26 , 50.7)	12.4 (-27, 51.88)	-1.53 (-34.81, 31.93)	**Ginge r+SoC**									
-7.92 (-19.2, 3.95)	12.93 (-12.56 , 39.19)	4.62 (-27.89 , 37.21)	4.15 (-30.21 , 38.89)	-9.82 (-36.25, 17.43)	-8.44 (-33.8, 17.74)	**Melato nin+So C**								
-3.23	17.65	8.36	7.81	-5.44	-3.65	4.5	**Vitami**							

(-34.62, 12.4)	(-23.48, 42.6)	(-35.84, 40.98)	(-37.3, 42.61)	(-46.68, 20.93)	(-44.45, 21.27)	(-29.57, 22.9)	**n D + SoC**							
-1.94 (-16.88, 15.36)	18.9 (-7.96, 48.34)	10.56 (-23.25, 45.72)	10.1 (-25.4, 46.91)	-3.9 (-31.55, 26.63)	-2.39 (-29.14, 27.09)	5.98 (-12.97, 26.89)	1.48 (-19.02, 39.31)	**Vitamin C + SoC**						
0.35 (-17.71, 17.9)	21.28 (-8.04, 50.16)	12.74 (-22.64, 47.73)	12.24 (-24.78, 49.14)	-1.57 (-31.82, 28.26)	-0.03 (-29.44, 28.72)	8.23 (-13.61, 28.97)	3.91 (-18.66, 40.49)	2.27 (-23.16, 24.9)	**Quercetin+SoC**					
-17.08 (-46.66, 12.61)	3.89 (-33.61, 41.33)	-4.68 (-47.37, 37.74)	-5.07 (-49.14, 38.96)	-19 (-57.13, 19.4)	-17.46 (-54.8, 19.97)	-9.26 (-41.14, 22.47)	-13.03 (-45.03, 30.79)	-15.21 (-49.88, 17.8)	-17.43 (-51.56, 17.52)	**Pomegranate juice + SoC**				
0.03 (-23.09, 23.43)	20.94 (-11.71, 54.12)	12.51 (-25.36, 50.35)	12.03 (-27.56, 51.9)	-1.91 (-35.21, 32.14)	-0.38 (-32.67, 32.67)	8.01 (-18.11, 34.12)	3.34 (-22.03, 44.56)	2.03 (-27.35, 29.19)	-0.3 (-29.26, 29.25)	17.08 (-20.04, 54.69)	**Curcumin+Piperine+SoC**			
-8.76 (-32.34, 14.6)	12.14 (-20.82, 45.12)	3.64 (-34.46, 41.8)	3.21 (-36.4, 42.88)	-10.68 (-44.12, 23.01)	-9.17 (-42.03, 23.75)	-0.79 (-27.2, 24.91)	-5.43 (-30.73, 35.57)	-6.77 (-36.47, 20.37)	-9.14 (-38.23, 20.48)	8.31 (-29.38, 45.78)	-8.76 (-41.89, 23.91)	**L+N+F+SoC**		
10.35 (-18, 38.38)	31.27 (-5, 67.26)	22.78 (-18.73,	22.3 (-20.67, 65.22)	8.41 (-28.67, 45.32)	9.91 (-26.45,	18.19 (-12.37,	14.24 (-16.24, 57.16)	12.23 (-21.15,	10.03 (-23.05, 43.31)	27.41 (-13.35, 67.98)	10.31 (-26.25, 46.51)	19.11 (-17.28,	**Nicotinamide +SOC**	

		63.97)			46.11)	48.19)		43.45)				55.54)		
-2.04 (-24.65, 20.84)	18.89 (-13.47, 51.68)	10.4 (-27.44, 48.31)	9.96 (-29.45, 49.07)	-3.95 (-36.83, 29.65)	-2.4 (-34.7, 30.19)	6.01 (-19.54, 31.28)	1.14 (-23.43, 42.41)	-0.04 (-29.2, 26.71)	-2.36 (-30.91, 26.99)	15.06 (-22.08, 52.19)	-2.05 (-34.82, 30.51)	6.75 (-25.85, 39.37)	-12.33 (-48.17, 23.68)	**Curcumin+Quercetin+SoC**

The data in the table are the MD (95% credible interval) of the intervention in the row compared with the intervention in the column. A larger MD represents a higher CRP; 0 represents no difference between the two interventions. Bolding represents a statistically significant decrease in CRP.

Table S9: League table for the comparison of the effects of different interventions versus SoC on the number of patients with headache

**SoC**		
2.32 (0.15, 85.88)	**Melatonin+SoC**	
5.49 (0.57, 160.38)	2.44 (0.04, 170.95)	**Quercetin+SoC**

The data in the table are the RR (95% credible interval) of the intervention in the row compared with the intervention in the column. A larger RR represents a higher clinical headache number; 1 represents no difference between the two interventions. Bolding represents a statistically significant decrease in clinical headache number.

Table S10: League table for the comparison of the effects of different interventions versus SoC on the number of deaths

**SoC**				
0.67 (0.26, 1.45)	**Vitamin D+SoC**			
1.05 (0.03, 30.78)	1.6 (0.04, 53.76)	**Vitamin K+SoC**		
0.54 (0.25, 1.1)	0.81 (0.27, 2.62)	0.51 (0.02, 19.85)	**Vitamin C+SoC**	
0.97 (0.02, 38.18)	1.47 (0.03, 61.79)	0.93 (0.01, 138.36)	1.8 (0.04, 77.41)	**Quercetin+ SoC**

The data in the table are the RR (95% credible interval) of the intervention in the row compared with the intervention in the column. A larger RR represents a higher clinical mortality number; 1 represents no difference between the two interventions. Bolding represents a statistically significant decrease in Clinical mortality number.

Table S11: League table for the comparison of the effects of different interventions versus SoC on the number of patients with fever

**SoC**				
1.51(0.5,5.71)	**Melatonin+ SoC**			

0.77(0.42,1.32)	0.5 (0.12, 1.78)	**Vitamin D + SoC**		
1.01(0.76,1.39)	0.67 (0.17, 2.13)	1.33 (0.72, 2.6)	**Quercetin+ SoC**	
0.95(0.61,1.48)	0.63 (0.16, 2.09)	1.25(0.62, 2.61)	0.9(0.54, 1.59)	**Curcumin+Piperine +SoC**

The data in the table are the RR (95% credible interval) of the intervention in the row compared with the intervention in the column. A larger RR represents a higher Fever; 1 represents no difference between the two interventions. Bolding represents a statistically significant decrease in Fever.

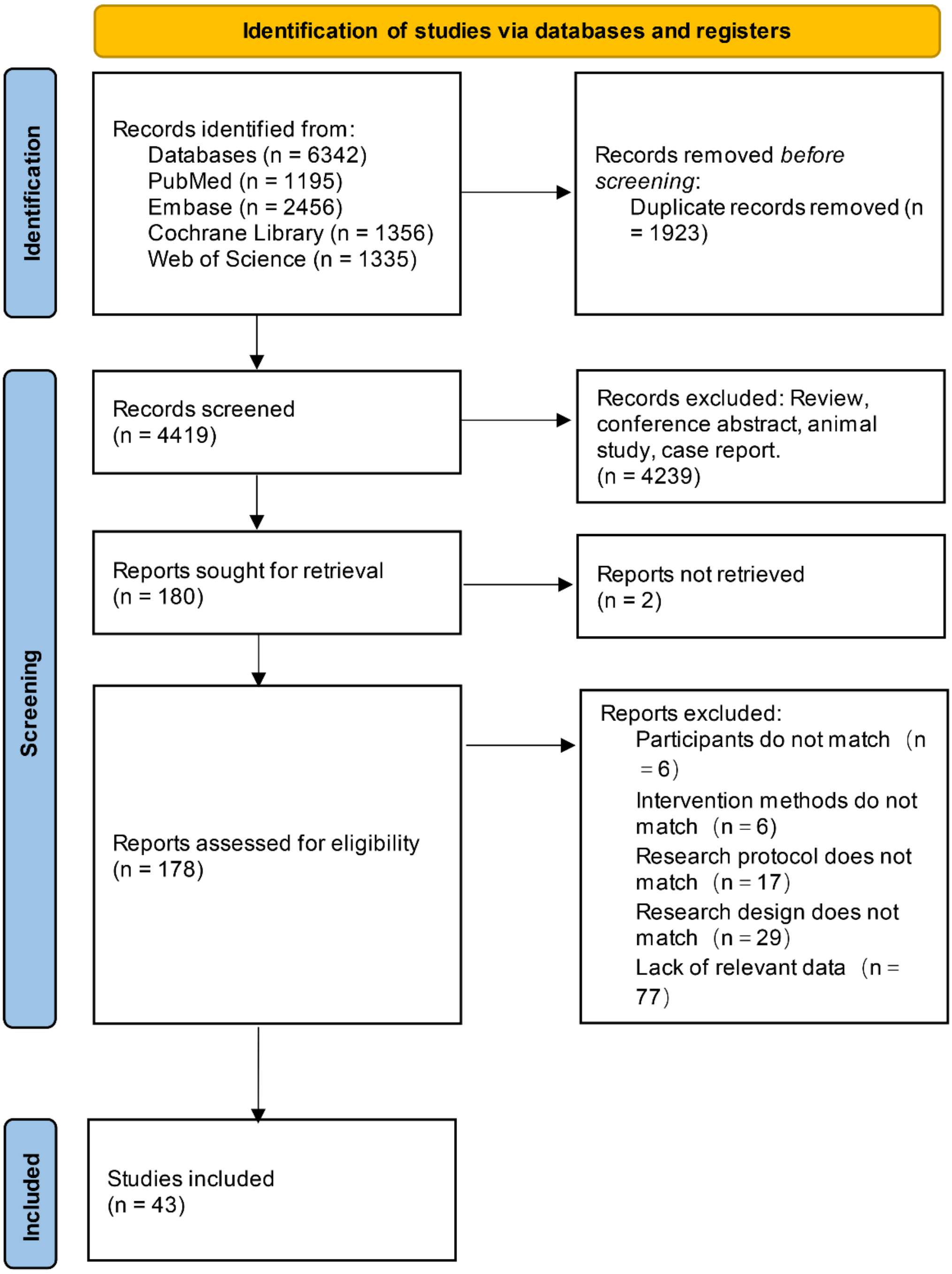

Figure S1: Flow diagram for literature search and screening

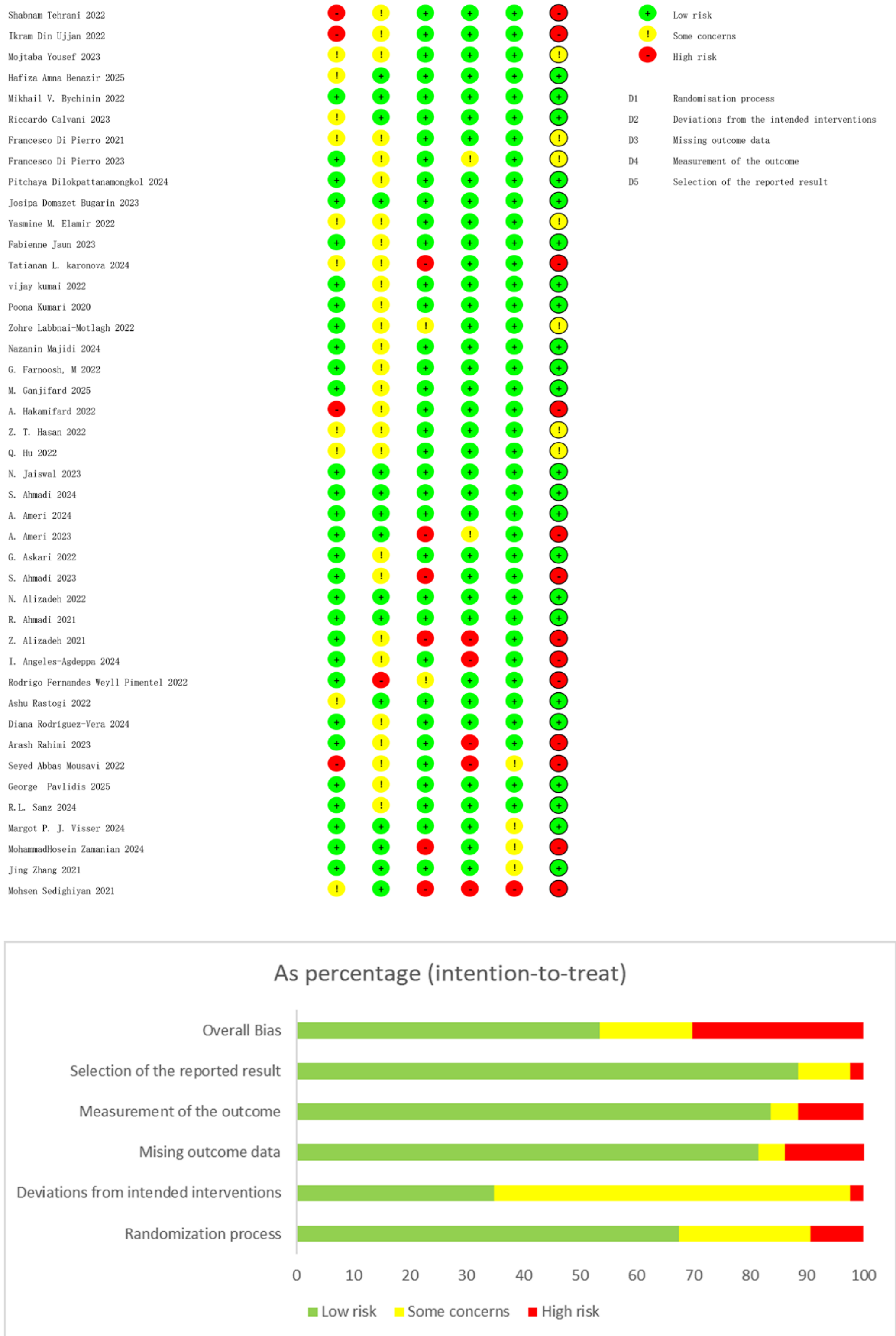

Figure S2: Quality assessment

Note: Each row represents an included study, and each column represents a domain of risk of bias. Color coding indicates the risk level: green = low risk; yellow = some

concerns; red = high risk. The last column on the right presents the overall risk of bias judgment for each study

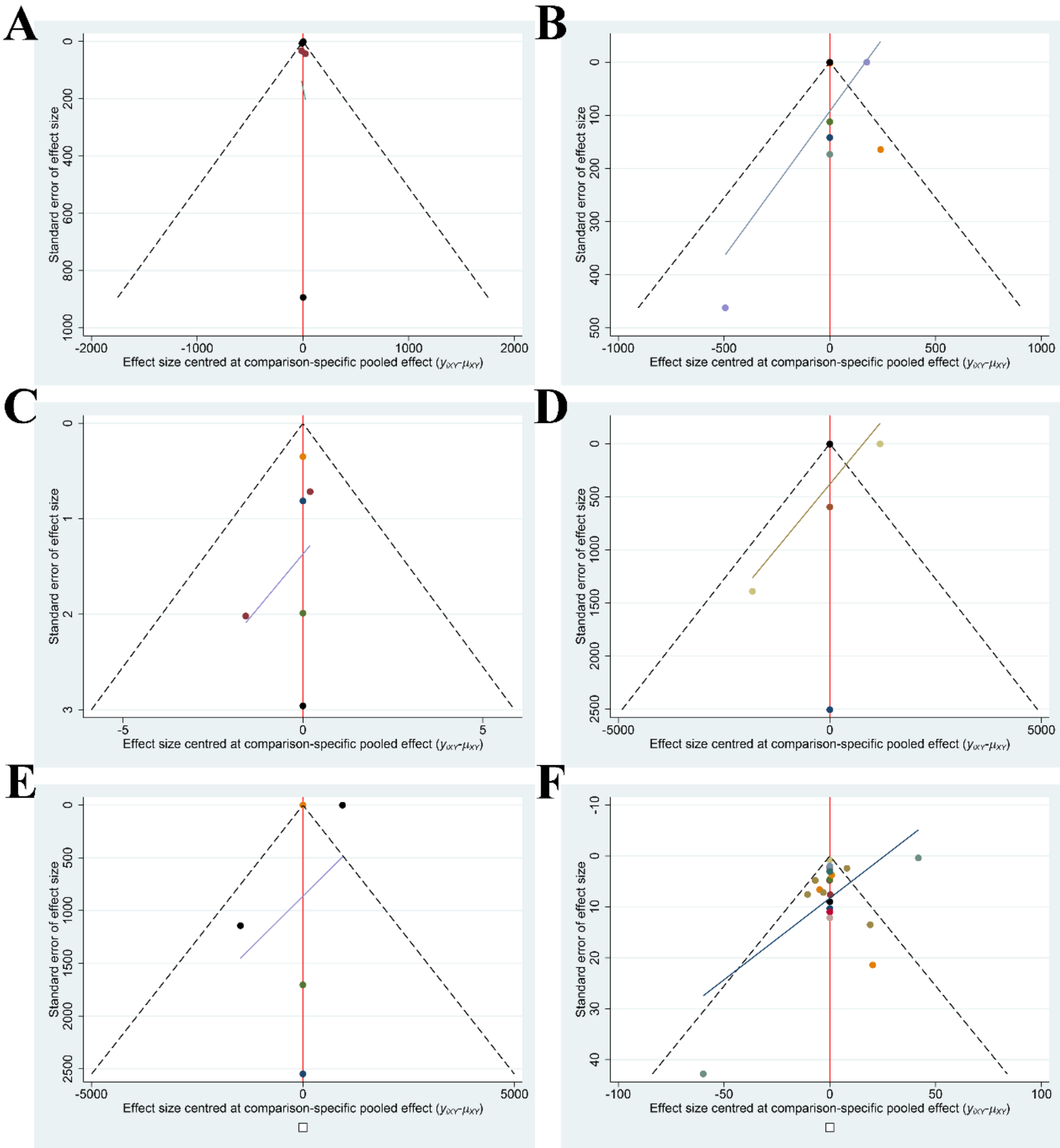

Figure S3: Funnel plots for (A) IL-6, (B) lymphocyte count, (C) NLR; (D) WBC count, (E) neutrophil count, and (F) CRP

Note: Each circle represents an included original study. The horizontal axis shows the effect size of each study relative to the common reference group, and the vertical axis shows the standard error (SE) of the effect size, with the vertical axis arranged in reverse order (namely, smaller SE values at the top stand for high-precision, large-sample studies, whereas larger SE values at the bottom represent low-precision, small-sample studies).

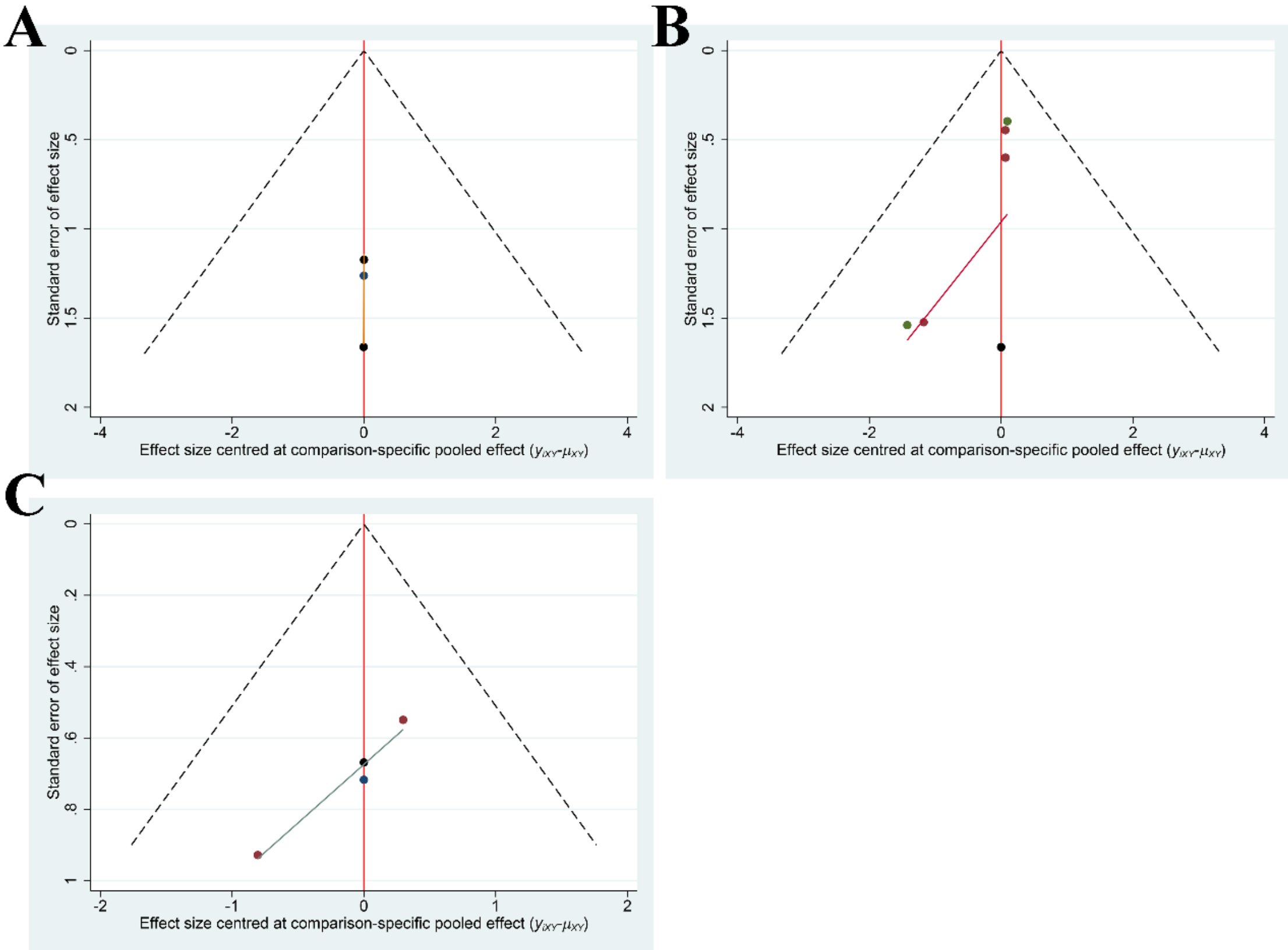

Figure S4: Funnel plots for (A) number of patients with headache, (B) number of deaths, and (C) number of patients with fever

Note: Each circle represents an included original study. The horizontal axis shows the effect size of each study relative to the common reference group, and the vertical axis shows the SE of the effect size, with the vertical axis arranged in reverse order (namely, smaller SE values at the top stand for high-precision, large-sample studies, whereas larger SE values at the bottom represent low-precision, small-sample studies).
