# Supplementary material for: Efficacy of supplementation with nutrients and dietary supplements on recovery and inflammatory response among patients with COVID-19: a network meta-analysis
Source: Front Nutr. 2026 Jul 28;13:1825539. doi: 10.3389/fnut.2026.1825539 (PMC13457122; doi:10.3389/fnut.2026.1825539)
Supplement: Supplementary file 3 [file Supplementary_file_3.pdf]

Supplementary Material 3: Convergence diagnosis graph for the Bayesian NMA model (PSRF fitting): Convergence of the results was assessed by operating potential scale reduction factors (PSRFs), with 1≤PSRF<1.05 representing successful convergence.

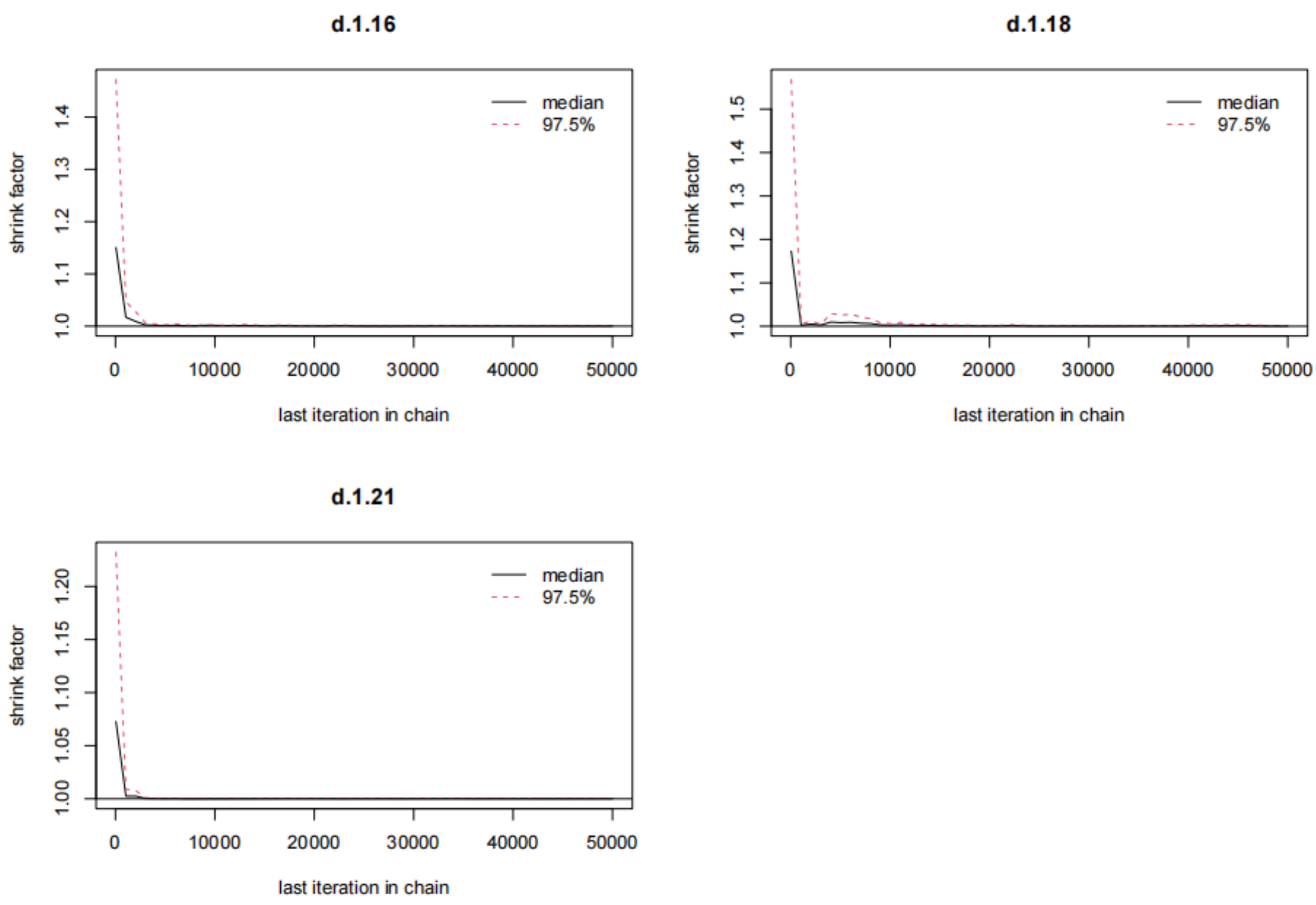

Figure SA: IL-6;

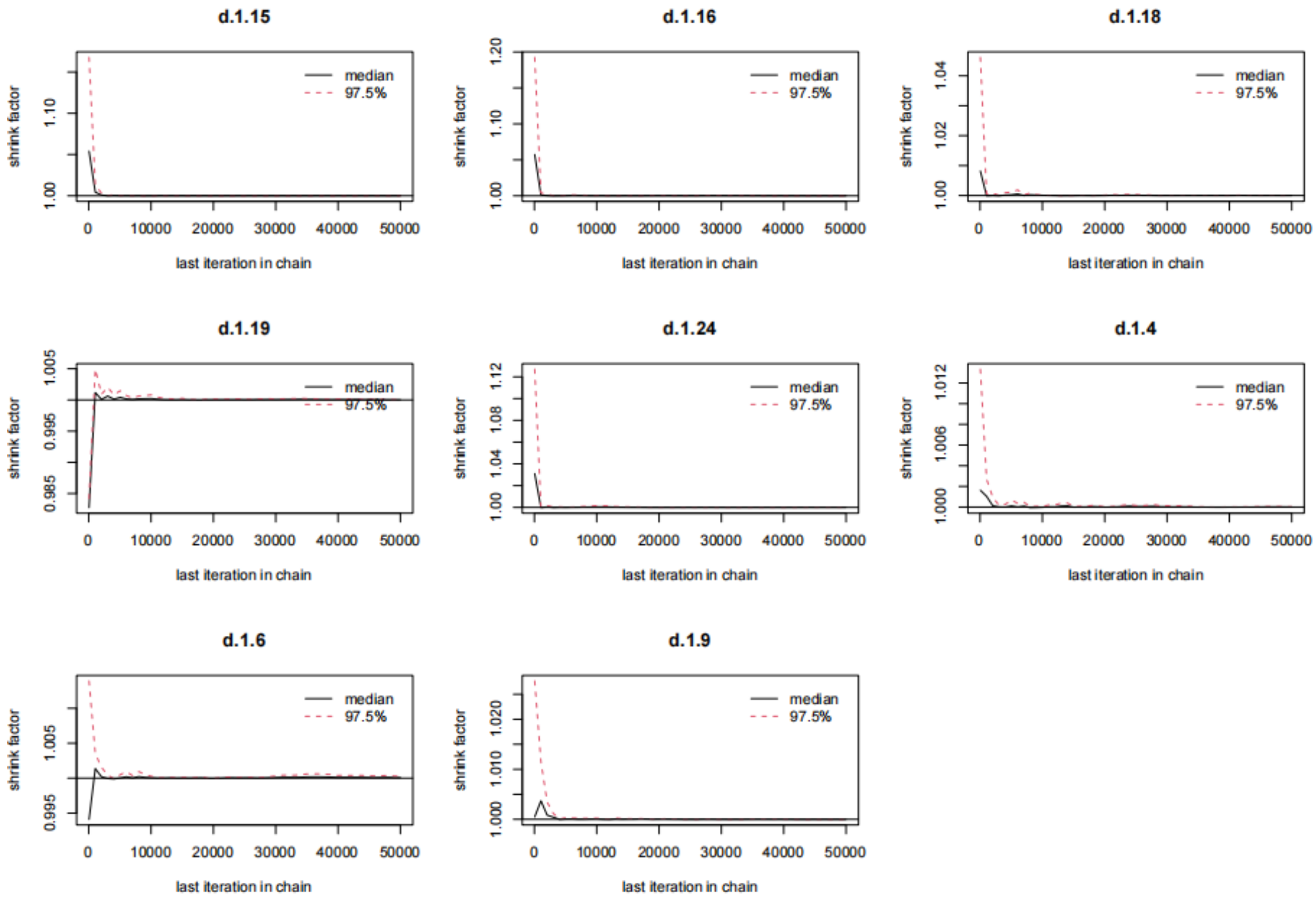

Figure SB: lymphocyte count;

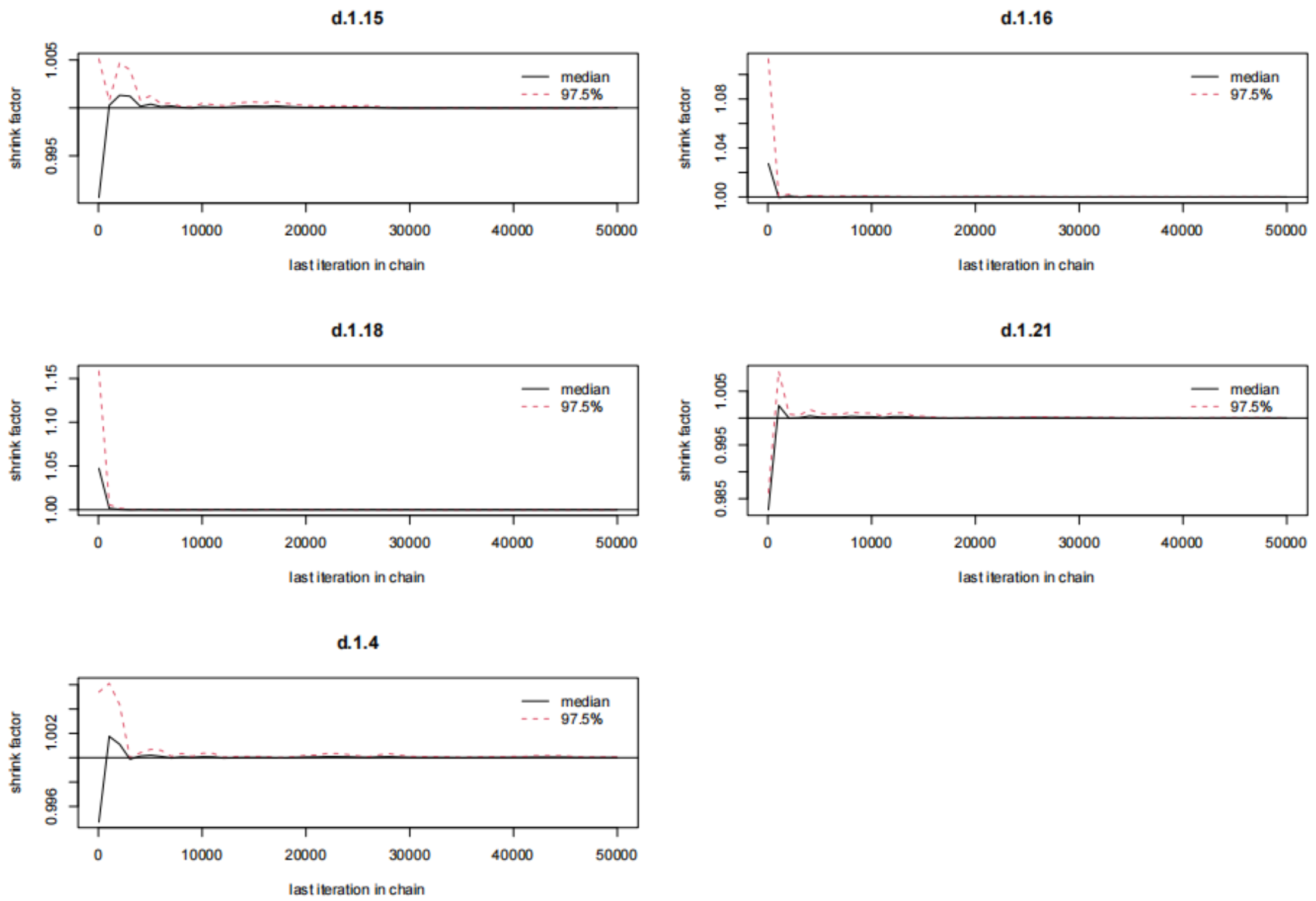

Figure SC: NLR;

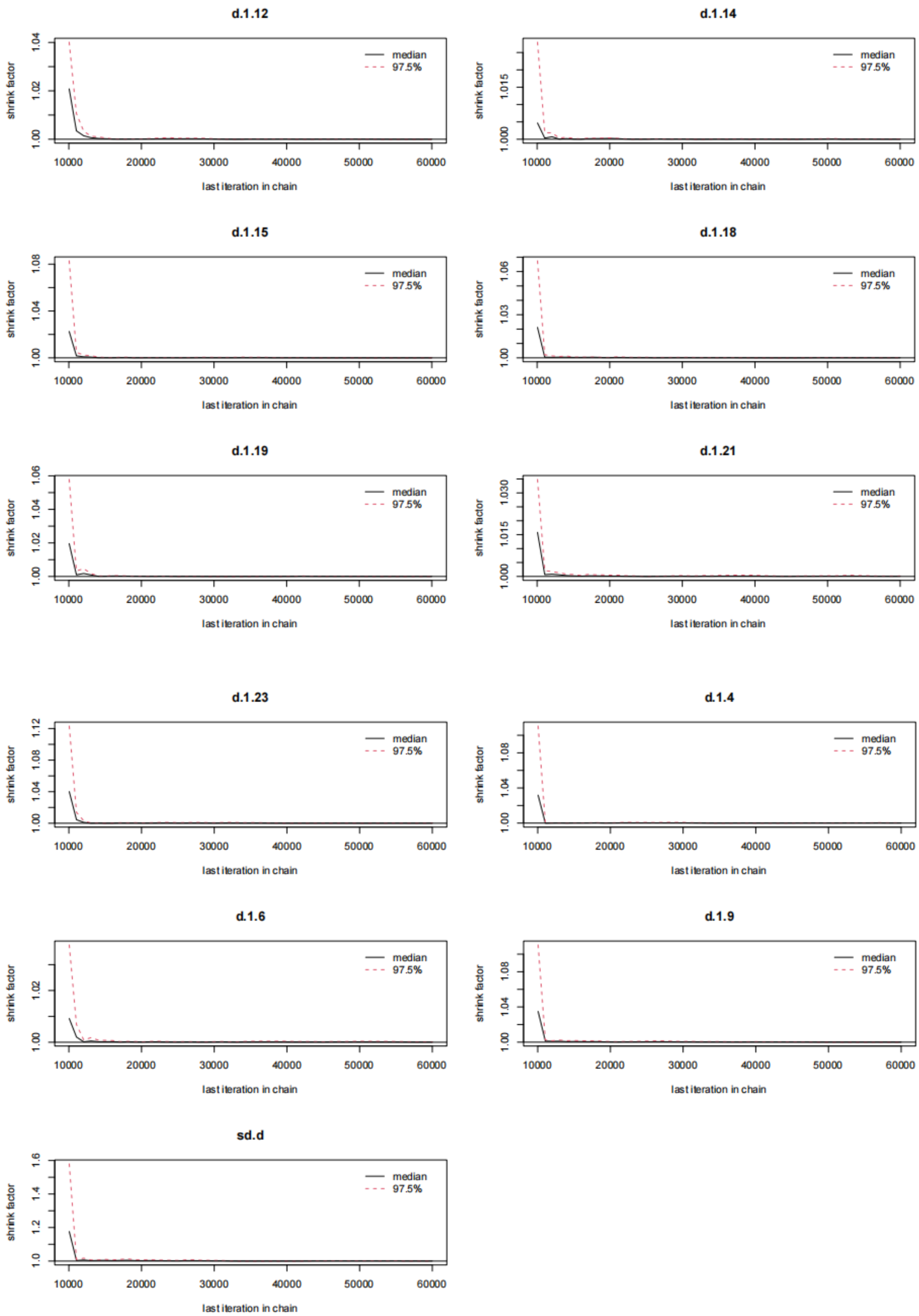

Figure SD: WBC;

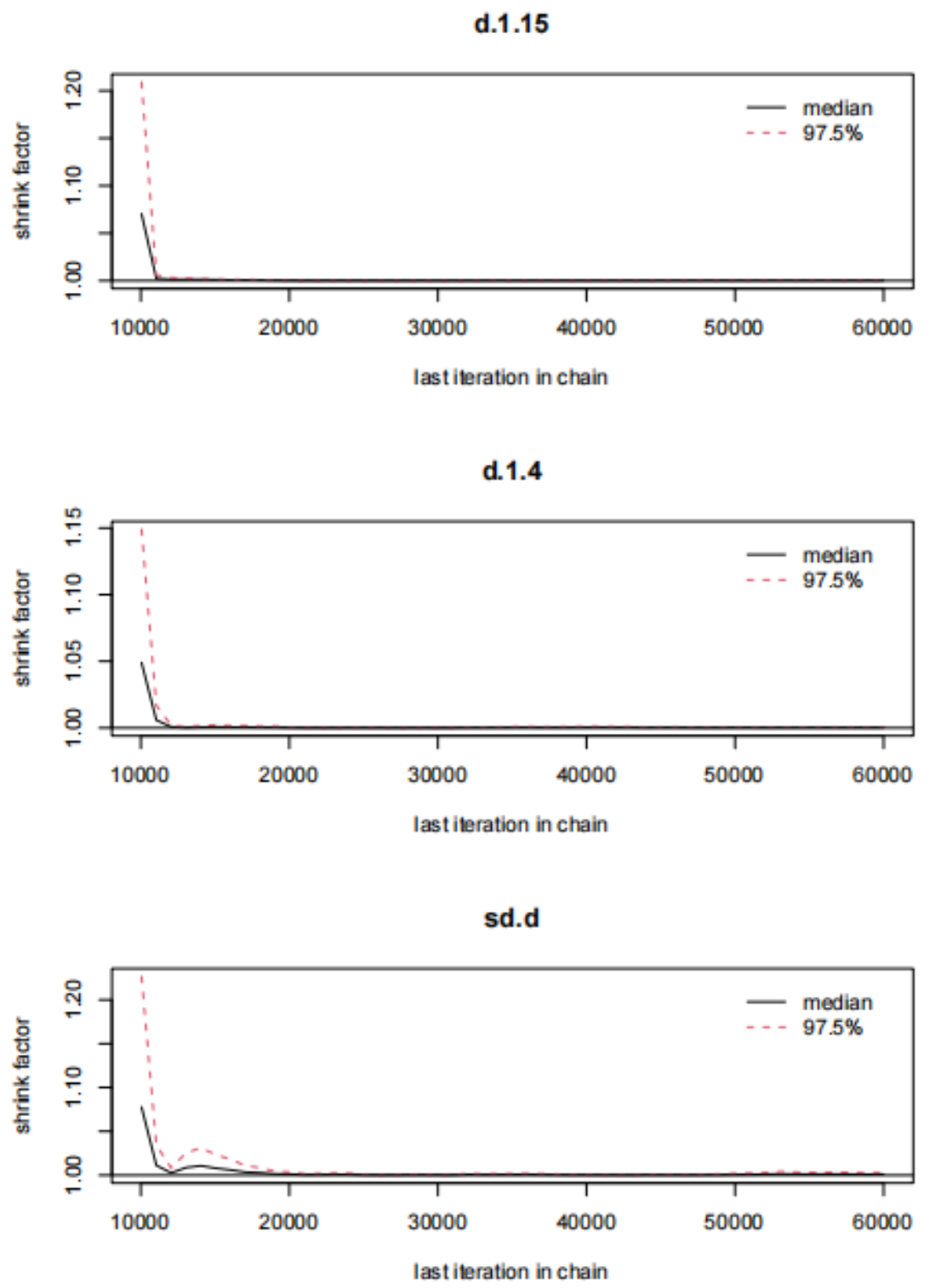

Figure SE: neutrophil count;

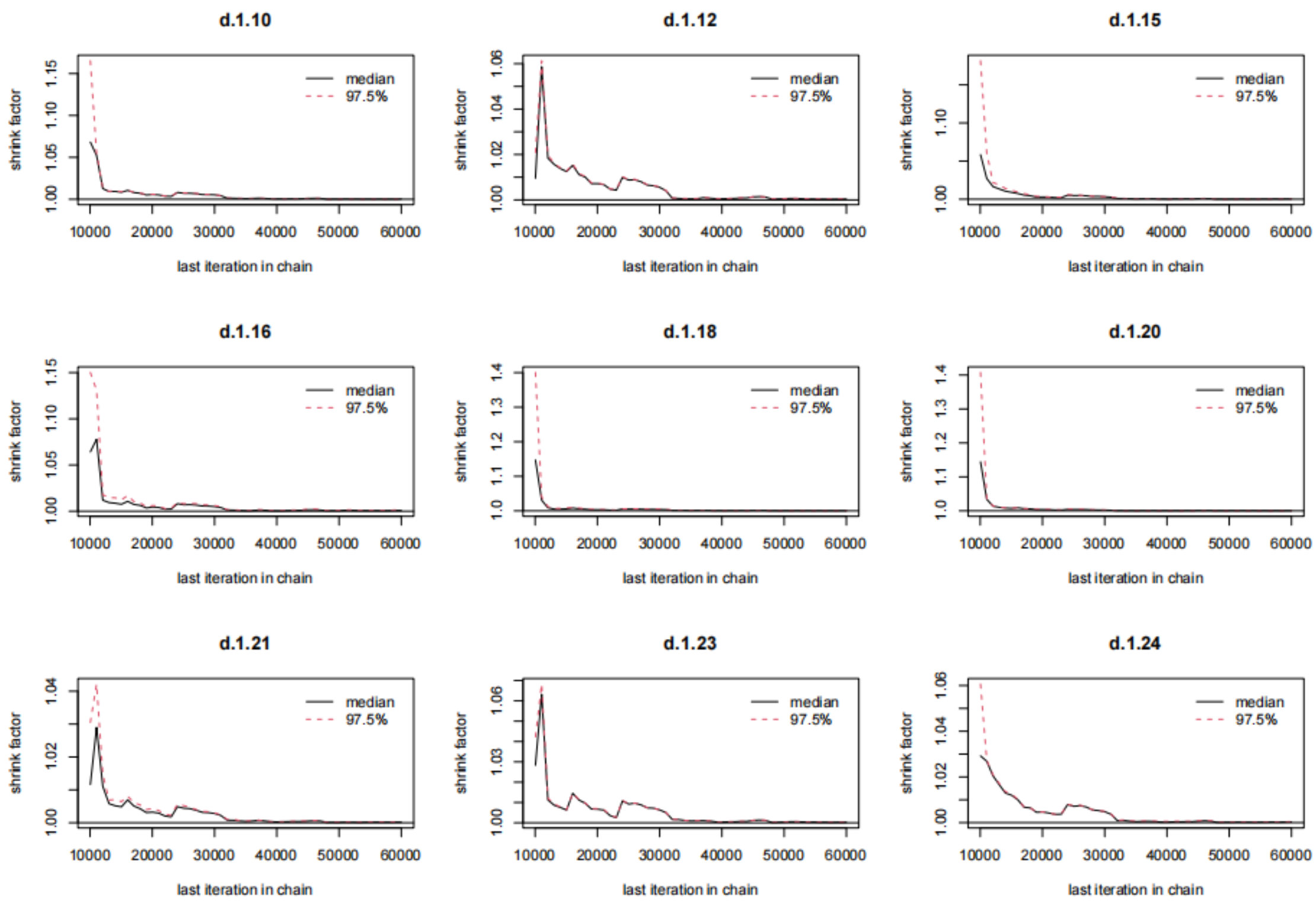

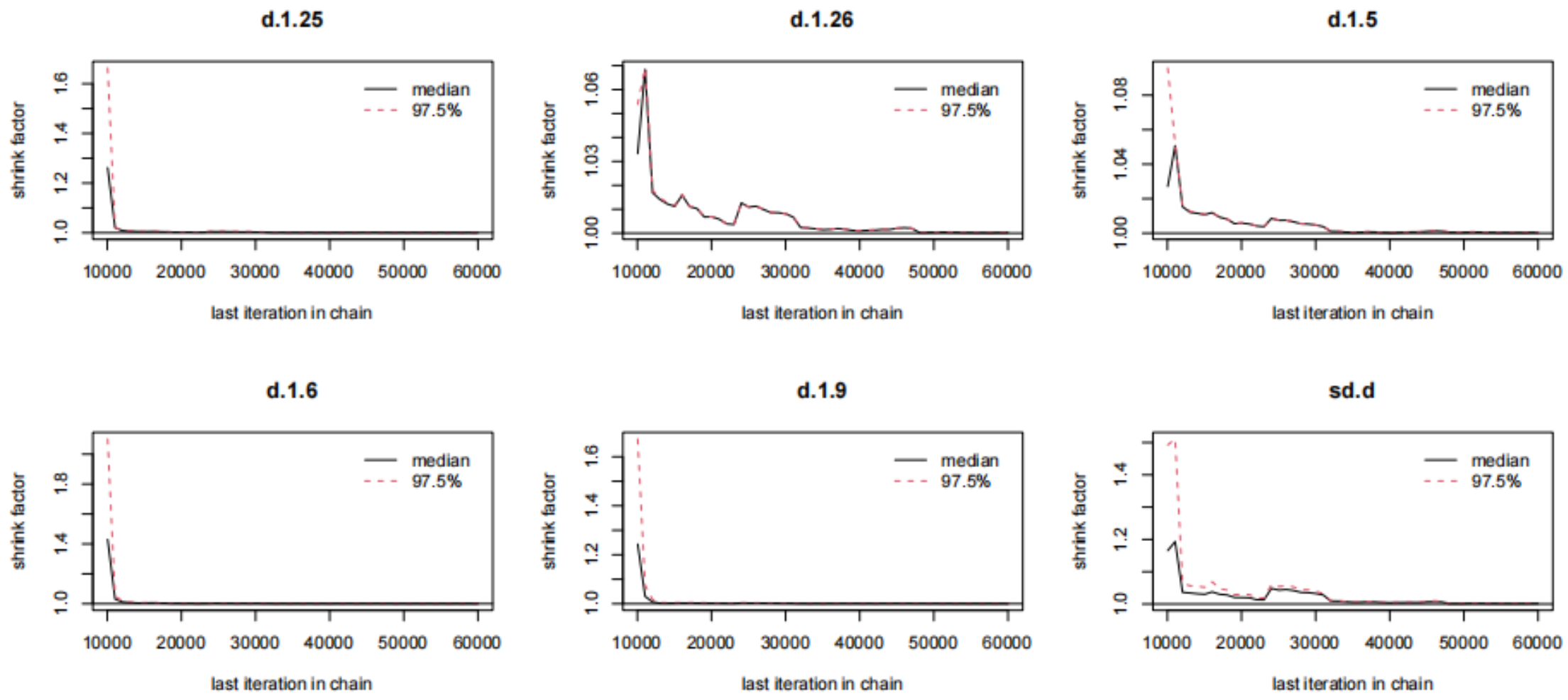

Figure SF: CRP;

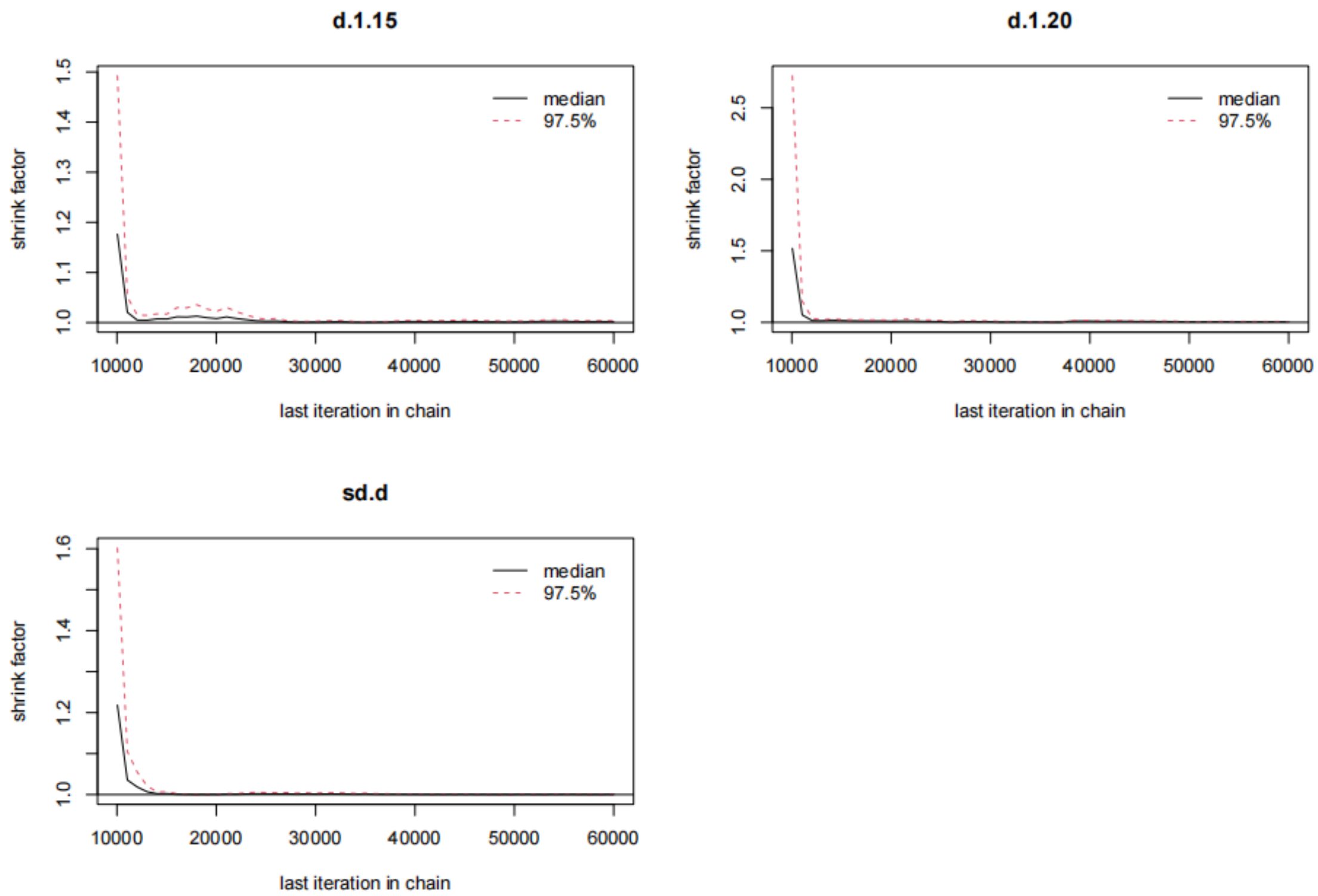

Figure SG: Number of patients with headache;

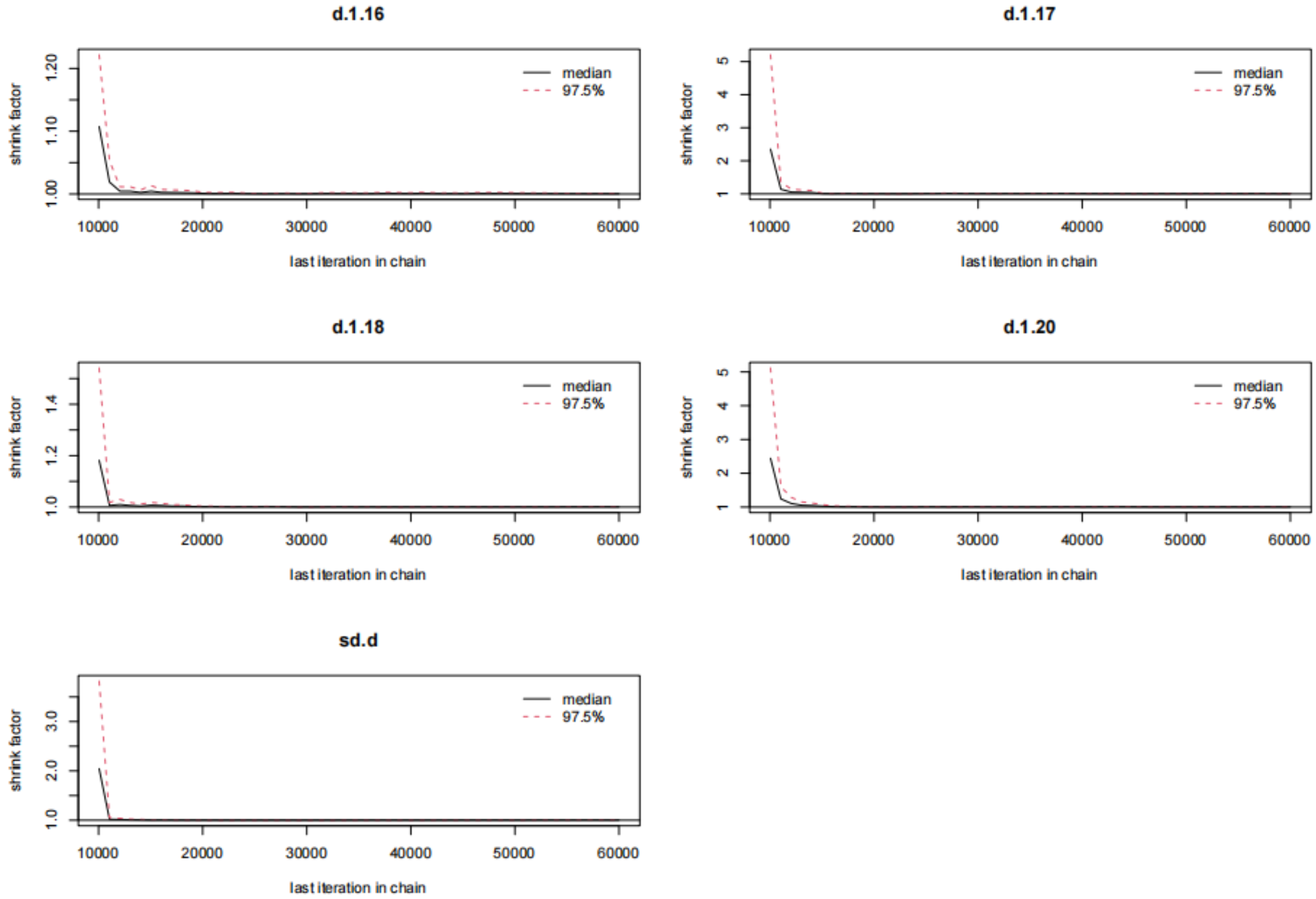

Figure SH: Number of deaths;

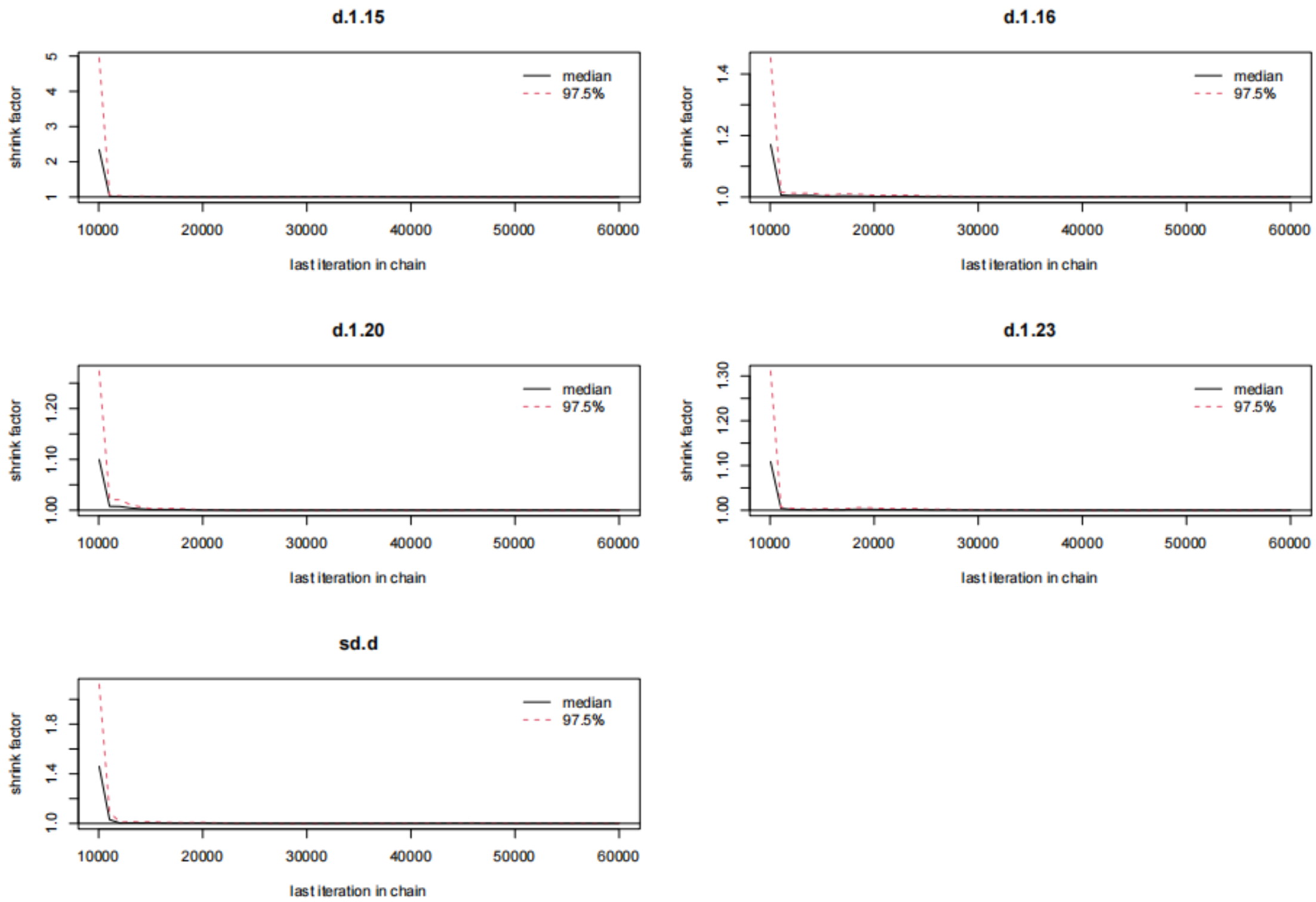

Figure SI: Number of patients with fever;

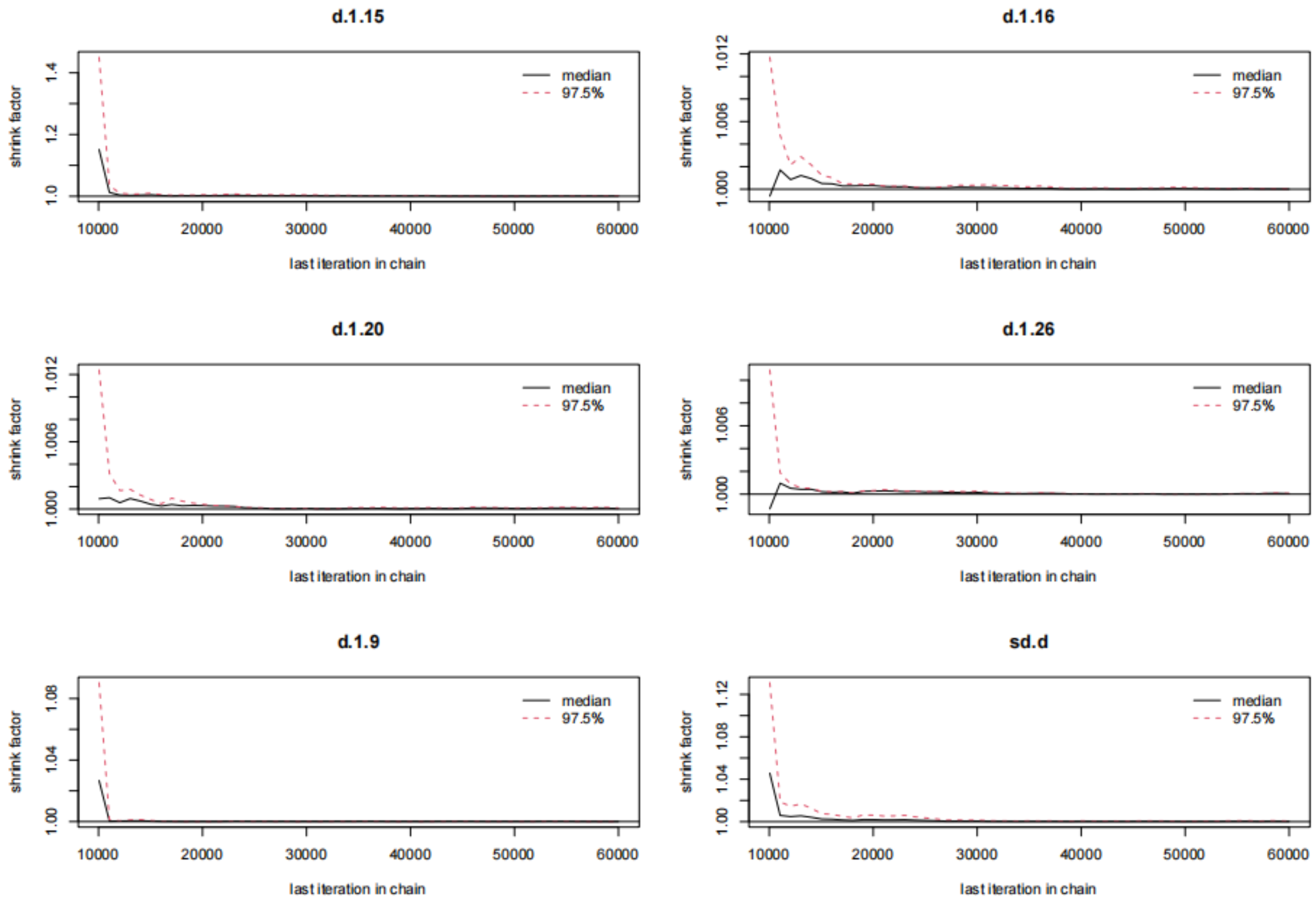

Figure SJ: Number of patients with cough.

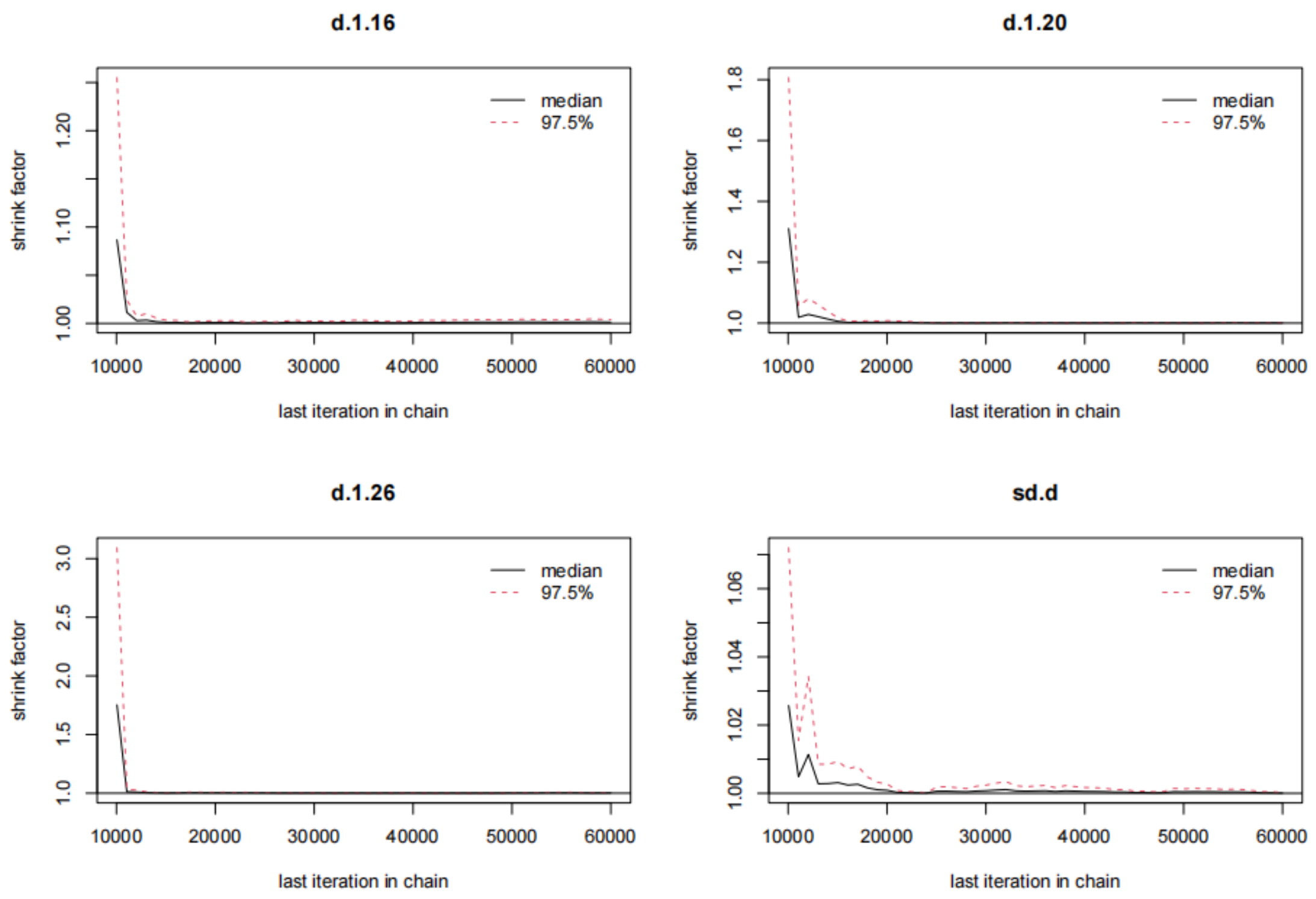

Figure SK: Number of patients with dyspnea.
