# Supplementary material for: Efficacy of supplementation with nutrients and dietary supplements on recovery and inflammatory response among patients with COVID-19: a network meta-analysis
Source: Front Nutr. 2026 Jul 28;13:1825539. doi: 10.3389/fnut.2026.1825539 (PMC13457122; doi:10.3389/fnut.2026.1825539)
Supplement: Supplementary file 4 [file Supplementary_file_4.pdf]

Supplementary Material 4: Certainty of evidence assessment (by CINeMA)

CRP

Comparison	Number of studies	Within-study bias	Reporting bias	Indirectness	Imprecision	Heterogeneity	Incoherence	Confidence rating
**Mixed evidence**								
Acetylcysteine+SoC:SoC	1	Major concerns	Low risk	No concerns	Major concerns	No concerns	Major concerns	Very low
Curcumin+Piperine+SoC:SoC	1	No concerns	Low risk	No concerns	Major concerns	No concerns	Major concerns	Very low
Curcumin+Quercetin+SoC:SoC	1	Major concerns	Low risk	No concerns	Major concerns	No concerns	Major concerns	Very low
Fatty acids+SoC:SoC	1	Major concerns	Low risk	No concerns	No concerns	No concerns	Major concerns	Very low
Ginger+SoC:SoC	1	No concerns	Low risk	No concerns	Major concerns	No concerns	Major concerns	Very low
L+N+F+SoC:SoC	1	Major concerns	Low risk	No concerns	Major concerns	No concerns	Major concerns	Very low
Melatonin+SoC:SoC	5	No concerns	Low risk	No concerns	Major concerns	No concerns	Major concerns	Very low
Nanocurcumin+SoC:SoC	1	No concerns	Low risk	No concerns	Major concerns	No concerns	Major concerns	Very low
Nicotinamide+SoC:SoC	1	Some concerns	Low risk	No concerns	Major concerns	No concerns	Major concerns	Very low
Pomegranate juice+SoC:SoC	1	Some concerns	Low risk	No concerns	Major concerns	No concerns	Major concerns	Very low
Quercetin+SoC:SoC	2	Some concerns	Low risk	No concerns	Major concerns	No concerns	Major concerns	Very low

SoC:Virgin coconut oil+SoC	1	Major concerns	Low risk	No concerns	Major concerns	No concerns	Major concerns	Very low
SoC:Vitamin C+SoC	3	No concerns	Low risk	No concerns	Major concerns	No concerns	Major concerns	Very low
SoC:Vitamin D+SoC	2	No concerns	Low risk	No concerns	Major concerns	No concerns	Major concerns	Very low
**Indirect evidence**								
Acetylcysteine+SoC:Curcumin+Piperine+SoC	0	No concerns	Low risk	No concerns	Major concerns	No concerns	Major concerns	Very low
Acetylcysteine+SoC:Curcumin+Quercetin+SoC	0	Major concerns	Low risk	No concerns	Major concerns	No concerns	Major concerns	Very low
Acetylcysteine+SoC:Fatty acids+SoC	0	Major concerns	Low risk	No concerns	No concerns	No concerns	Major concerns	Very low
Acetylcysteine+SoC:Ginger+SoC	0	No concerns	Low risk	No concerns	Major concerns	No concerns	Major concerns	Very low
Acetylcysteine+SoC:L+N+F+SoC	0	Major concerns	Low risk	No concerns	Major concerns	No concerns	Major concerns	Very low
Acetylcysteine+SoC:Melatonin+SoC	0	Major concerns	Low risk	No concerns	Major concerns	No concerns	Major concerns	Very low
Acetylcysteine+SoC:Nanocurcumin+SoC	0	No concerns	Low risk	No concerns	Major concerns	No concerns	Major concerns	Very low
Acetylcysteine+SoC:Nicotinamide+SoC	0	Some concerns	Low risk	No concerns	Major concerns	No concerns	Major concerns	Very low
Acetylcysteine+SoC:Pomegranate juice+SoC	0	Some concerns	Low risk	No concerns	Major concerns	No concerns	Major concerns	Very low

Acetylcysteine+SoC:Quercetin+SoC	0	Some concerns	Low risk	No concerns	Major concerns	No concerns	Major concerns	Very low
Acetylcysteine+SoC:Virgin coconut oil+SoC	0	Major concerns	Low risk	No concerns	Major concerns	No concerns	Major concerns	Very low
Acetylcysteine+SoC:Vitamin C+SoC	0	Major concerns	Low risk	No concerns	Major concerns	No concerns	Major concerns	Very low
Acetylcysteine+SoC:Vitamin D+SoC	0	No concerns	Low risk	No concerns	Major concerns	No concerns	Major concerns	Very low
Curcumin+Piperine+SoC:Curcumin +Quercetin+SoC	0	No concerns	Low risk	No concerns	Major concerns	No concerns	Major concerns	Very low
Curcumin+Piperine+SoC:Fatty acids+SoC	0	No concerns	Low risk	No concerns	No concerns	No concerns	Major concerns	Low
Curcumin+Piperine+SoC:Ginger+SoC	0	No concerns	Low risk	No concerns	Major concerns	No concerns	Major concerns	Very low
Curcumin+Piperine+SoC:L+N+F+SoC	0	No concerns	Low risk	No concerns	Major concerns	No concerns	Major concerns	Very low
Curcumin+Piperine+SoC:Melatonin +SoC	0	No concerns	Low risk	No concerns	Major concerns	No concerns	Major concerns	Very low
Curcumin+Piperine+SoC:Nanocurcumin+SoC	0	No concerns	Low risk	No concerns	Major concerns	No concerns	Major concerns	Very low
Curcumin+Piperine+SoC:Nicotinamide+SoC	0	No concerns	Low risk	No concerns	Major concerns	No concerns	Major concerns	Very low
Curcumin+Piperine+SoC:Pomegranate juice+SoC	0	No concerns	Low risk	No concerns	Major concerns	No concerns	Major concerns	Very low
Curcumin+Piperine+SoC:Quercetin +SoC	0	No concerns	Low risk	No concerns	Major concerns	No concerns	Major concerns	Very low

Curcumin+Piperine+SoC:Virgin coconut oil+SoC	0	No concerns	Low risk	No concerns	Major concerns	No concerns	Major concerns	Very low
Curcumin+Piperine+SoC:Vitamin C+SoC	0	No concerns	Low risk	No concerns	Major concerns	No concerns	Major concerns	Very low
Curcumin+Piperine+SoC:Vitamin D+SoC	0	No concerns	Low risk	No concerns	Major concerns	No concerns	Major concerns	Very low
Curcumin+Quercetin+SoC:Fatty acids+SoC	0	Major concerns	Low risk	No concerns	No concerns	No concerns	Major concerns	Very low
Curcumin+Quercetin+SoC:Ginger+SoC	0	No concerns	Low risk	No concerns	Major concerns	No concerns	Major concerns	Very low
Curcumin+Quercetin+SoC:L+N+F+SoC	0	Major concerns	Low risk	No concerns	Major concerns	No concerns	Major concerns	Very low
Curcumin+Quercetin+SoC:Melatonin+SoC	0	Major concerns	Low risk	No concerns	Major concerns	No concerns	Major concerns	Very low
Curcumin+Quercetin+SoC:Nanocurcumin+SoC	0	No concerns	Low risk	No concerns	Major concerns	No concerns	Major concerns	Very low
Curcumin+Quercetin+SoC:Nicotinamide+SoC	0	Some concerns	Low risk	No concerns	Major concerns	No concerns	Major concerns	Very low
Curcumin+Quercetin+SoC:Pomegranate juice+SoC	0	Some concerns	Low risk	No concerns	Major concerns	No concerns	Major concerns	Very low
Curcumin+Quercetin+SoC:Quercetin+SoC	0	Some concerns	Low risk	No concerns	Major concerns	No concerns	Major concerns	Very low
Curcumin+Quercetin+SoC:Virgin coconut oil+SoC	0	Major concerns	Low risk	No concerns	Major concerns	No concerns	Major concerns	Very low
Curcumin+Quercetin+SoC:Vitamin C+SoC	0	Major concerns	Low risk	No concerns	Major concerns	No concerns	Major concerns	Very low

Curcumin+Quercetin+SoC:Vitamin D+SoC	0	No concerns	Low risk	No concerns	Major concerns	No concerns	Major concerns	Very low
Fatty acids+SoC:Ginger+SoC	0	No concerns	Low risk	No concerns	No concerns	No concerns	Major concerns	Low
Fatty acids+SoC:L+N+F+SoC	0	Major concerns	Low risk	No concerns	No concerns	No concerns	Major concerns	Very low
Fatty acids+SoC:Melatonin+SoC	0	Major concerns	Low risk	No concerns	No concerns	No concerns	Major concerns	Very low
Fatty acids+SoC:Nanocurcumin+SoC	0	No concerns	Low risk	No concerns	No concerns	No concerns	Major concerns	Low
Fatty acids+SoC:Nicotinamide+SoC	0	Some concerns	Low risk	No concerns	No concerns	No concerns	Major concerns	Low
Fatty acids+SoC:Pomegranate juice+SoC	0	Some concerns	Low risk	No concerns	No concerns	No concerns	Major concerns	Low
Fatty acids+SoC:Quercetin+SoC	0	Some concerns	Low risk	No concerns	No concerns	No concerns	Major concerns	Low
Fatty acids+SoC:Virgin coconut oil+SoC	0	Major concerns	Low risk	No concerns	No concerns	No concerns	Major concerns	Very low
Fatty acids+SoC:Vitamin C+SoC	0	Major concerns	Low risk	No concerns	No concerns	No concerns	Major concerns	Very low
Fatty acids+SoC:Vitamin D+SoC	0	No concerns	Low risk	No concerns	No concerns	No concerns	Major concerns	Low
Ginger+SoC:L+N+F+SoC	0	No concerns	Low risk	No concerns	Major concerns	No concerns	Major concerns	Very low
Ginger+SoC:Melatonin+SoC	0	No concerns	Low risk	No concerns	Major concerns	No concerns	Major concerns	Very low

Ginger+SoC:Nanocurcumin+SoC	0	No concerns	Low risk	No concerns	Major concerns	No concerns	Major concerns	Very low
Ginger+SoC:Nicotinamide+SoC	0	No concerns	Low risk	No concerns	Major concerns	No concerns	Major concerns	Very low
Ginger+SoC:Pomegranate juice+SoC	0	No concerns	Low risk	No concerns	Major concerns	No concerns	Major concerns	Very low
Ginger+SoC:Quercetin+SoC	0	No concerns	Low risk	No concerns	Major concerns	No concerns	Major concerns	Very low
Ginger+SoC:Virgin coconut oil+SoC	0	No concerns	Low risk	No concerns	Major concerns	No concerns	Major concerns	Very low
Ginger+SoC:Vitamin C+SoC	0	No concerns	Low risk	No concerns	Major concerns	No concerns	Major concerns	Very low
Ginger+SoC:Vitamin D+SoC	0	No concerns	Low risk	No concerns	Major concerns	No concerns	Major concerns	Very low
L+N+F+SoC:Melatonin+SoC	0	Major concerns	Low risk	No concerns	Major concerns	No concerns	Major concerns	Very low
L+N+F+SoC:Nanocurcumin+SoC	0	No concerns	Low risk	No concerns	Major concerns	No concerns	Major concerns	Very low
L+N+F+SoC:Nicotinamide+SoC	0	Some concerns	Low risk	No concerns	Major concerns	No concerns	Major concerns	Very low
L+N+F+SoC:Pomegranate juice+SoC	0	Some concerns	Low risk	No concerns	Major concerns	No concerns	Major concerns	Very low
L+N+F+SoC:Quercetin+SoC	0	Some concerns	Low risk	No concerns	Major concerns	No concerns	Major concerns	Very low
L+N+F+SoC:Virgin coconut oil+SoC	0	Major concerns	Low risk	No concerns	Major concerns	No concerns	Major concerns	Very low

L+N+F+SoC:Vitamin C+SoC	0	Major concerns	Low risk	No concerns	Major concerns	No concerns	Major concerns	Very low
L+N+F+SoC:Vitamin D+SoC	0	No concerns	Low risk	No concerns	Major concerns	No concerns	Major concerns	Very low
Melatonin+SoC:Nanocurcumin+SoC	0	No concerns	Low risk	No concerns	Major concerns	No concerns	Major concerns	Very low
Melatonin+SoC:Nicotinamide+SoC	0	Some concerns	Low risk	No concerns	Major concerns	No concerns	Major concerns	Very low
Melatonin+SoC:Pomegranate juice+SoC	0	Some concerns	Low risk	No concerns	Major concerns	No concerns	Major concerns	Very low
Melatonin+SoC:Quercetin+SoC	0	Some concerns	Low risk	No concerns	Major concerns	No concerns	Major concerns	Very low
Melatonin+SoC:Virgin coconut oil+SoC	0	Major concerns	Low risk	No concerns	Major concerns	No concerns	Major concerns	Very low
Melatonin+SoC:Vitamin C+SoC	0	No concerns	Low risk	No concerns	Major concerns	No concerns	Major concerns	Very low
Melatonin+SoC:Vitamin D+SoC	0	No concerns	Low risk	No concerns	Major concerns	No concerns	Major concerns	Very low
Nanocurcumin+SoC:Nicotinamide+SoC	0	No concerns	Low risk	No concerns	Major concerns	No concerns	Major concerns	Very low
Nanocurcumin+SoC:Pomegranate juice+SoC	0	No concerns	Low risk	No concerns	Major concerns	No concerns	Major concerns	Very low
Nanocurcumin+SoC:Quercetin+SoC	0	No concerns	Low risk	No concerns	Major concerns	No concerns	Major concerns	Very low
Nanocurcumin+SoC:Virgin coconut oil+SoC	0	No concerns	Low risk	No concerns	Major concerns	No concerns	Major concerns	Very low

Nanocurcumin+SoC:Vitamin C+SoC	0	No concerns	Low risk	No concerns	Major concerns	No concerns	Major concerns	Very low
Nanocurcumin+SoC:Vitamin D+SoC	0	No concerns	Low risk	No concerns	Major concerns	No concerns	Major concerns	Very low
Nicotinamide+SoC:Pomegranate juice+SoC	0	Some concerns	Low risk	No concerns	Major concerns	No concerns	Major concerns	Very low
Nicotinamide+SoC:Quercetin+SoC	0	Some concerns	Low risk	No concerns	Major concerns	No concerns	Major concerns	Very low
Nicotinamide+SoC:Virgin coconut oil+SoC	0	Some concerns	Low risk	No concerns	Major concerns	No concerns	Major concerns	Very low
Nicotinamide+SoC:Vitamin C+SoC	0	Some concerns	Low risk	No concerns	Major concerns	No concerns	Major concerns	Very low
Nicotinamide+SoC:Vitamin D+SoC	0	No concerns	Low risk	No concerns	Major concerns	No concerns	Major concerns	Very low
Pomegranate juice+SoC:Quercetin+SoC	0	Some concerns	Low risk	No concerns	Major concerns	No concerns	Major concerns	Very low
Pomegranate juice+SoC:Virgin coconut oil+SoC	0	Some concerns	Low risk	No concerns	Major concerns	No concerns	Major concerns	Very low
Pomegranate juice+SoC:Vitamin C+SoC	0	Some concerns	Low risk	No concerns	Major concerns	No concerns	Major concerns	Very low
Pomegranate juice+SoC:Vitamin D+SoC	0	No concerns	Low risk	No concerns	Major concerns	No concerns	Major concerns	Very low
Quercetin+SoC:Virgin coconut oil+SoC	0	Some concerns	Low risk	No concerns	Major concerns	No concerns	Major concerns	Very low
Quercetin+SoC:Vitamin C+SoC	0	Some concerns	Low risk	No concerns	Major concerns	No concerns	Major concerns	Very low

Quercetin+SoC:Vitamin D+SoC	0	No concerns	Low risk	No concerns	Major concerns	No concerns	Major concerns	Very low
Virgin coconut oil+SoC:Vitamin C+SoC	0	Major concerns	Low risk	No concerns	Major concerns	No concerns	Major concerns	Very low
Virgin coconut oil+SoC:Vitamin D+SoC	0	No concerns	Low risk	No concerns	Major concerns	No concerns	Major concerns	Very low
Vitamin C+SoC:Vitamin D+SoC	0	No concerns	Low risk	No concerns	Major concerns	No concerns	Major concerns	Very low

IL-6

Comparison	Number of studies	Within-study bias	Reporting bias	Indirectness	Imprecision	Heterogeneity	Incoherence	Confidence rating
Mixed evidence								
Pomegranate juice+SoC:SoC	1	Some concerns	Low risk	No concerns	No concerns	Major concerns	No concerns	Low
SoC:Vitamin C+SoC	2	No concerns	Low risk	No concerns	No concerns	Major concerns	No concerns	Low
SoC:Vitamin D+SoC	3	No concerns	Low risk	No concerns	Major concerns	No concerns	No concerns	Low
Indirect evidence								
Pomegranate juice+SoC:Vitamin C+SoC	0	No concerns	Low risk	No concerns	Major concerns	No concerns	No concerns	Low
Pomegranate juice+SoC:Vitamin D+SoC	0	Some concerns	Low risk	No concerns	Major concerns	No concerns	No concerns	Low
Vitamin C+SoC:Vitamin D+SoC	0	No concerns	Low risk	No concerns	Major concerns	No concerns	No concerns	Low

Nlr

Comparison	Number of studies	Within-study bias	Reporting bias	Indirectness	Imprecision	Heterogeneity	Incoherence	Confidence rating
Mixed evidence								
Glycocalyx+SoC:SoC	1	No concerns	Low risk	No concerns	NA	NA	NA	Very low
Melatonin+SoC:SoC	2	No concerns	Low risk	No concerns	NA	NA	NA	Very low
Pomegranate juice+SoC:SoC	1	Some concerns	Low risk	No concerns	NA	NA	NA	Very low
SoC:Vitamin C+SoC	1	Some concerns	Low risk	No concerns	NA	NA	NA	Very low
SoC:Vitamin D+SoC	1	No concerns	Low risk	No concerns	NA	NA	NA	Very low
Indirect evidence								
Glycocalyx+SoC:Melatonin+SoC	0	No concerns	Low risk	No concerns	NA	NA	NA	Very low
Glycocalyx+SoC:Pomegranate juice+SoC	0	No concerns	Low risk	No concerns	NA	NA	NA	Very low
Glycocalyx+SoC:Vitamin C+SoC	0	No concerns	Low risk	No concerns	NA	NA	NA	Very low
Glycocalyx+SoC:Vitamin D+SoC	0	No concerns	Low risk	No concerns	NA	NA	NA	Very low
Melatonin+SoC:Pomegranate juice+SoC	0	No concerns	Low risk	No concerns	NA	NA	NA	Very low
Melatonin+SoC:Vitamin C+SoC	0	No concerns	Low risk	No concerns	NA	NA	NA	Very low
Melatonin+SoC:Vitamin D+SoC	0	No concerns	Low risk	No concerns	NA	NA	NA	Very low
Pomegranate juice+SoC:Vitamin C+SoC	0	Some concerns	Low risk	No concerns	NA	NA	NA	Very low

Comparison	Number of studies	Within-study bias	Reporting bias	Indirectness	Imprecision	Heterogeneity	Incoherence	Confidence rating
Mixed evidence								
Melatonin+SoC:SoC	1	No concerns	Low risk	No concerns	No concerns	No concerns	No concerns	High
Quercetin+SoC:SoC	2	Some concerns	Low risk	No concerns	No concerns	No concerns	No concerns	Moderate
Indirect evidence								
Melatonin+SoC:Quercetin+SoC	0	No concerns	Low risk	No concerns	No concerns	No concerns	No concerns	High
Pomegranate juice+SoC:Vitamin D+SoC	0	No concerns	Low risk	No concerns	NA	NA	NA	Very low
Vitamin C+SoC:Vitamin D+SoC	0	No concerns	Low risk	No concerns	NA	NA	NA	Very low

Headache

Fever

Comparison	Number of studies	Within-study bias	Reporting bias	Indirectness	Imprecision	Heterogeneity	Incoherence	Confidence rating
Mixed evidence								
Curcumin+Quercetin+SoC:SoC	1	Major concerns	Low risk	No concerns	No concerns	No concerns	No concerns	Low
Melatonin+SoC:SoC	1	No concerns	Low risk	No concerns	No concerns	No concerns	No concerns	High
Quercetin+SoC:SoC	2	Some concerns	Low risk	No concerns	No concerns	No concerns	No concerns	Moderate
SoC:Vitamin D+SoC	1	Major concerns	Low risk	No concerns	No concerns	No concerns	No concerns	Low
Indirect evidence								
Curcumin+Quercetin+SoC:Melatonin+SoC	0	No concerns	Low risk	No concerns	No concerns	No concerns	No concerns	High
Curcumin+Quercetin+SoC:Quercetin+SoC	0	Major concerns	Low risk	No concerns	No concerns	No concerns	No concerns	Low
Curcumin+Quercetin+SoC:Vitamin D+SoC	0	Major concerns	Low risk	No concerns	No concerns	No concerns	No concerns	Low
Melatonin+SoC:Quercetin+SoC	0	No concerns	Low risk	No concerns	No concerns	No concerns	No concerns	High
Melatonin+SoC:Vitamin D+SoC	0	No concerns	Low risk	No concerns	No concerns	No concerns	No concerns	High
Quercetin+SoC:Vitamin D+SoC	0	Major concerns	Low risk	No concerns	No concerns	No concerns	No concerns	Low

death

Comparison	Number of studies	Within-study bias	Reporting bias	Indirectness	Imprecision	Heterogeneity	Incoherence	Confidence rating
Mixed evidence								
Quercetin+SoC:SoC	1	Some concerns	Low risk	No concerns	No concerns	No concerns	No concerns	Moderate
SoC:Vitamin C+SoC	3	No concerns	Low risk	No concerns	No concerns	No concerns	No concerns	High
SoC:Vitamin D+SoC	2	No concerns	Low risk	No concerns	No concerns	No concerns	No concerns	High
SoC:Vitamin K+SoC	1	No concerns	Low risk	No concerns	No concerns	No concerns	No concerns	High
Indirect evidence								
Quercetin+SoC:Vitamin C+SoC	0	Some concerns	Low risk	No concerns	No concerns	No concerns	No concerns	Moderate
Quercetin+SoC:Vitamin D+SoC	0	Some concerns	Low risk	No concerns	No concerns	No concerns	No concerns	Moderate
Quercetin+SoC:Vitamin K+SoC	0	No concerns	Low risk	No concerns	No concerns	No concerns	No concerns	High
Vitamin C+SoC:Vitamin D+SoC	0	No concerns	Low risk	No concerns	No concerns	No concerns	No concerns	High
Vitamin C+SoC:Vitamin K+SoC	0	No concerns	Low risk	No concerns	No concerns	No concerns	No concerns	High
Vitamin D+SoC:Vitamin K+SoC	0	No concerns	Low risk	No concerns	No concerns	No concerns	No concerns	High

Cough

Comparison	Number of studies	Within-study bias	Reporting bias	Indirectness	Imprecision	Heterogeneity	Incoherence	Confidence rating
Mixed evidence								
Curcumin+Quercetin+SoC:SoC	1	Major concerns	Low risk	No concerns	No concerns	No concerns	No concerns	Low
Melatonin+SoC:SoC	1	No concerns	Low risk	No concerns	No concerns	No concerns	No concerns	High
Nanocurcumin+SoC:SoC	1	No concerns	Low risk	No concerns	No concerns	No concerns	No concerns	High
Quercetin+SoC:SoC	2	Some concerns	Low risk	No concerns	No concerns	No concerns	No concerns	Moderate
SoC:Vitamin D+SoC	1	Major concerns	Low risk	No concerns	No concerns	No concerns	No concerns	Low
Indirect evidence								
Curcumin+Quercetin+SoC:Melatonin+SoC	0	No concerns	Low risk	No concerns	No concerns	No concerns	No concerns	High
Curcumin+Quercetin+SoC:Nanocurcumin+SoC	0	No concerns	Low risk	No concerns	No concerns	No concerns	No concerns	High
Curcumin+Quercetin+SoC:Quercetin+SoC	0	Some concerns	Low risk	No concerns	No concerns	No concerns	No concerns	Moderate
Curcumin+Quercetin+SoC:Vitamin D+SoC	0	Major concerns	Low risk	No concerns	No concerns	No concerns	No concerns	Low
Melatonin+SoC:Nanocurcumin+SoC	0	No concerns	Low risk	No concerns	No concerns	No concerns	No concerns	High

Melatonin+SoC:Quercetin+SoC	0	No concerns	Low risk	No concerns	No concerns	No concerns	No concerns	High
Melatonin+SoC:Vitamin D+SoC	0	No concerns	Low risk	No concerns	No concerns	No concerns	No concerns	High
Nanocurcumin+SoC:Quercetin+SoC	0	No concerns	Low risk	No concerns	No concerns	No concerns	No concerns	High
Nanocurcumin+SoC:Vitamin D+SoC	0	No concerns	Low risk	No concerns	No concerns	No concerns	No concerns	High
Quercetin+SoC:Vitamin D+SoC	0	Some concerns	Low risk	No concerns	No concerns	No concerns	No concerns	Moderate

Dyspnea

Comparison	Number of studies	Within-study bias	Reporting bias	Indirectness	Imprecision	Heterogeneity	Incoherence	Confidence rating
Mixed evidence								
Curcumin+Quercetin+SoC:SoC	1	Major concerns	Low risk	No concerns	No concerns	No concerns	No concerns	Low
Quercetin+SoC:SoC	1	Some concerns	Low risk	No concerns	No concerns	No concerns	No concerns	Moderate
Indirect evidence								
SoC:Vitamin D+SoC	1	Major concerns	Low risk	No concerns	No concerns	No concerns	No concerns	Low
Curcumin+Quercetin+SoC:Quercetin+SoC	0	Some concerns	Low risk	No concerns	No concerns	No concerns	No concerns	Moderate
Curcumin+Quercetin+SoC:Vitamin D+SoC	0	Major concerns	Low risk	No concerns	No concerns	No concerns	No concerns	Low
Quercetin+SoC:Vitamin D+SoC	0	Some concerns	Low risk	No concerns	No concerns	No concerns	No concerns	Moderate

Neutrophil Count

Comparison	Number of studies	Within-study bias	Reporting bias	Indirectness	Imprecision	Heterogeneity	Incoherence	Confidence rating
Mixed evidence								
Acetylcysteine+SoC:SoC	1	Major concerns	Low risk	No concerns	NA	NA	NA	Low
Glycocalyx+SoC:SoC	1	No concerns	Low risk	No concerns	NA	NA	NA	High
Melatonin+SoC:SoC	1	No concerns	Low risk	No concerns	NA	NA	NA	High
SoC:Vitamin B+SoC	1	No concerns	Low risk	No concerns	NA	NA	NA	High
SoC:Vitamin C+SoC	2	No concerns	Low risk	No concerns	NA	NA	NA	High
Indirect evidence								
Acetylcysteine+SoC:Glycocalyx+SoC	0	No concerns	Low risk	No concerns	NA	NA	NA	High
Acetylcysteine+SoC:Melatonin+SoC	0	No concerns	Low risk	No concerns	NA	NA	NA	High
Acetylcysteine+SoC:Vitamin B+SoC	0	No concerns	Low risk	No concerns	NA	NA	NA	High
Acetylcysteine+SoC:Vitamin C+SoC	0	Major concerns	Low risk	No concerns	NA	NA	NA	Low
Glycocalyx+SoC:Melatonin+SoC	0	No concerns	Low risk	No concerns	NA	NA	NA	High

Glycocalyx+SoC:Vitamin B+SoC	0	No concerns	Low risk	No concerns	NA	NA	NA	High
Glycocalyx+SoC:Vitamin C+SoC	0	No concerns	Low risk	No concerns	NA	NA	NA	High
Melatonin+SoC:Vitamin B+SoC	0	No concerns	Low risk	No concerns	NA	NA	NA	High
Melatonin+SoC:Vitamin C+SoC	0	No concerns	Low risk	No concerns	NA	NA	NA	High
Vitamin B+SoC:Vitamin C+SoC	0	No concerns	Low risk	No concerns	NA	NA	NA	High

WBC

Comparison	Number of studies	Within-study bias	Reporting bias	Indirectness	Imprecision	Heterogeneity	Incoherence	Confidence rating
Mixed evidence								
Acetylcysteine+SoC:SoC	1	Major concerns	Low risk	No concerns	NA	NA	NA	Very low
Curcumin+Piperine+SoC:SoC	1	No concerns	Low risk	No concerns	NA	NA	NA	Very low
Ginger+SoC:SoC	1	No concerns	Low risk	No concerns	NA	NA	NA	Very low
Glycocalyx+SoC:SoC	1	No concerns	Low risk	No concerns	NA	NA	NA	Very low
Melatonin+SoC:SoC	1	Major concerns	Low risk	No concerns	NA	NA	NA	Very low
Nanocurcumin+SoC:SoC	1	No concerns	Low risk	No concerns	NA	NA	NA	Very low
Pomegranate juice+SoC:SoC	1	Some concerns	Low risk	No concerns	NA	NA	NA	Very low

SoC:Vitamin B+SoC	1	No concerns	Low risk	No concerns	NA	NA	NA	Very low
SoC:Vitamin C+SoC	2	No concerns	Low risk	No concerns	NA	NA	NA	Very low
SoC:Vitamin C+Vitamin E+SoC	1	Major concerns	Low risk	No concerns	NA	NA	NA	Very low
Indirect evidence								
Acetylcysteine+SoC:Curcumin+Piperine+SoC	0	No concerns	Low risk	No concerns	NA	NA	NA	Very low
Acetylcysteine+SoC:Ginger+SoC	0	No concerns	Low risk	No concerns	NA	NA	NA	Very low
Acetylcysteine+SoC:Glycocalyx+SoC	0	No concerns	Low risk	No concerns	NA	NA	NA	Very low
Acetylcysteine+SoC:Melatonin+SoC	0	Major concerns	Low risk	No concerns	NA	NA	NA	Very low
Acetylcysteine+SoC:Nanocurcumin+SoC	0	No concerns	Low risk	No concerns	NA	NA	NA	Very low
Acetylcysteine+SoC:Pomegranate juice+SoC	0	Some concerns	Low risk	No concerns	NA	NA	NA	Very low
Acetylcysteine+SoC:Vitamin B+SoC	0	No concerns	Low risk	No concerns	NA	NA	NA	Very low
Acetylcysteine+SoC:Vitamin C+SoC	0	Major concerns	Low risk	No concerns	NA	NA	NA	Very low
Acetylcysteine+SoC:Vitamin C+Vitamin E+SoC	0	Major concerns	Low risk	No concerns	NA	NA	NA	Very low

Curcumin+Piperine+SoC:Ginger+SoC	0	No concerns	Low risk	No concerns	NA	NA	NA	Very low
Curcumin+Piperine+SoC:Glycocalyx+SoC	0	No concerns	Low risk	No concerns	NA	NA	NA	Very low
Curcumin+Piperine+SoC:Melatonin+SoC	0	No concerns	Low risk	No concerns	NA	NA	NA	Very low
Curcumin+Piperine+SoC:Nanocurcumin+SoC	0	No concerns	Low risk	No concerns	NA	NA	NA	Very low
Curcumin+Piperine+SoC:Pomegranate juice+SoC	0	No concerns	Low risk	No concerns	NA	NA	NA	Very low
Curcumin+Piperine+SoC:Vitamin B+SoC	0	No concerns	Low risk	No concerns	NA	NA	NA	Very low
Curcumin+Piperine+SoC:Vitamin C+SoC	0	No concerns	Low risk	No concerns	NA	NA	NA	Very low
Curcumin+Piperine+SoC:Vitamin C+Vitamin E+SoC	0	No concerns	Low risk	No concerns	NA	NA	NA	Very low
Ginger+SoC:Glycocalyx+SoC	0	No concerns	Low risk	No concerns	NA	NA	NA	Very low
Ginger+SoC:Melatonin+SoC	0	No concerns	Low risk	No concerns	NA	NA	NA	Very low
Ginger+SoC:Nanocurcumin+SoC	0	No concerns	Low risk	No concerns	NA	NA	NA	Very low
Ginger+SoC:Pomegranate juice+SoC	0	No concerns	Low risk	No concerns	NA	NA	NA	Very low
Ginger+SoC:Vitamin B+SoC	0	No concerns	Low risk	No concerns	NA	NA	NA	Very low

Ginger+SoC:Vitamin C+SoC	0	No concerns	Low risk	No concerns	NA	NA	NA	Very low
Ginger+SoC:Vitamin C+Vitamin E+SoC	0	No concerns	Low risk	No concerns	NA	NA	NA	Very low
Glycocalyx+SoC:Melatonin+SoC	0	No concerns	Low risk	No concerns	NA	NA	NA	Very low
Glycocalyx+SoC:Nanocurcumin+SoC	0	No concerns	Low risk	No concerns	NA	NA	NA	Very low
Glycocalyx+SoC:Pomegranate juice+SoC	0	No concerns	Low risk	No concerns	NA	NA	NA	Very low
Glycocalyx+SoC:Vitamin B+SoC	0	No concerns	Low risk	No concerns	NA	NA	NA	Very low
Glycocalyx+SoC:Vitamin C+SoC	0	No concerns	Low risk	No concerns	NA	NA	NA	Very low
Glycocalyx+SoC:Vitamin C+Vitamin E+SoC	0	No concerns	Low risk	No concerns	NA	NA	NA	Very low
Melatonin+SoC:Nanocurcumin+SoC	0	No concerns	Low risk	No concerns	NA	NA	NA	Very low
Melatonin+SoC:Pomegranate juice+SoC	0	Some concerns	Low risk	No concerns	NA	NA	NA	Very low
Melatonin+SoC:Vitamin B+SoC	0	No concerns	Low risk	No concerns	NA	NA	NA	Very low
Melatonin+SoC:Vitamin C+SoC	0	Major concerns	Low risk	No concerns	NA	NA	NA	Very low
Melatonin+SoC:Vitamin C+Vitamin E+SoC	0	Major concerns	Low risk	No concerns	NA	NA	NA	Very low

Nanocurcumin+SoC:Pomegranate juice+SoC	0	No concerns	Low risk	No concerns	NA	NA	NA	Very low
Nanocurcumin+SoC:Vitamin B+SoC	0	No concerns	Low risk	No concerns	NA	NA	NA	Very low
Nanocurcumin+SoC:Vitamin C+SoC	0	No concerns	Low risk	No concerns	NA	NA	NA	Very low
Nanocurcumin+SoC:Vitamin C+Vitamin E+SoC	0	No concerns	Low risk	No concerns	NA	NA	NA	Very low
Pomegranate juice+SoC:Vitamin B+SoC	0	No concerns	Low risk	No concerns	NA	NA	NA	Very low
Pomegranate juice+SoC:Vitamin C+SoC	0	Some concerns	Low risk	No concerns	NA	NA	NA	Very low
Pomegranate juice+SoC:Vitamin C+Vitamin E+SoC	0	Some concerns	Low risk	No concerns	NA	NA	NA	Very low
Vitamin B+SoC:Vitamin C+SoC	0	No concerns	Low risk	No concerns	NA	NA	NA	Very low
Vitamin B+SoC:Vitamin C+Vitamin E+SoC	0	No concerns	Low risk	No concerns	NA	NA	NA	Very low
Vitamin C+SoC:Vitamin C+Vitamin E+SoC	0	Major concerns	Low risk	No concerns	NA	NA	NA	Very low

lymphocyte count

Comparison	Number of studies	Within-study bias	Reporting bias	Indirectness	Imprecision	Heterogeneity	Incoherence	Confidence rating
Mixed evidence								
Acetylcysteine+SoC:SoC	1	Major concerns	Low risk	No concerns	Major concerns	No concerns	Some concerns	Very low

Glycocalyx+SoC:SoC	1	No concerns	Low risk	No concerns	Major concerns	No concerns	Some concerns	Low
L+N+F+SoC:SoC	1	Major concerns	Low risk	No concerns	No concerns	Major concerns	Some concerns	Very low
Melatonin+SoC:SoC	3	No concerns	Low risk	No concerns	Major concerns	No concerns	Some concerns	Low
Nanocurcumin+SoC:SoC	1	No concerns	Low risk	No concerns	No concerns	No concerns	Some concerns	Moderate
SoC:Vitamin B+SoC	1	No concerns	Low risk	No concerns	Major concerns	No concerns	Some concerns	Low
SoC:Vitamin C+SoC	2	No concerns	Low risk	No concerns	Major concerns	No concerns	Some concerns	Low
SoC:Vitamin D+SoC	1	No concerns	Low risk	No concerns	Major concerns	No concerns	Some concerns	Low
Indirect evidence								
Acetylcysteine+SoC:Glycocalyx+SoC	0	No concerns	Low risk	No concerns	Major concerns	No concerns	Some concerns	Low
Acetylcysteine+SoC:L+N+F+SoC	0	Major concerns	Low risk	No concerns	No concerns	Major concerns	Some concerns	Very low
Acetylcysteine+SoC:Melatonin+SoC	0	Major concerns	Low risk	No concerns	Major concerns	No concerns	Some concerns	Very low
Acetylcysteine+SoC:Nanocurcumin+SoC	0	No concerns	Low risk	No concerns	No concerns	No concerns	Some concerns	Moderate
Acetylcysteine+SoC:Vitamin B+SoC	0	No concerns	Low risk	No concerns	Major concerns	No concerns	Some concerns	Low
Acetylcysteine+SoC:Vitamin C+SoC	0	Major concerns	Low risk	No concerns	Major concerns	No concerns	Some concerns	Very low
Acetylcysteine+SoC:Vitamin D+SoC	0	No concerns	Low risk	No concerns	Major concerns	No concerns	Some concerns	Low
Glycocalyx+SoC:L+N+F+SoC	0	No concerns	Low risk	No concerns	No concerns	Major concerns	Some concerns	Low
Glycocalyx+SoC:Melatonin+SoC	0	No concerns	Low risk	No concerns	Major concerns	No concerns	Some concerns	Low
Glycocalyx+SoC:Nanocurcumin+SoC	0	No concerns	Low risk	No concerns	No concerns	No concerns	Some concerns	Moderate
Glycocalyx+SoC:Vitamin B+SoC	0	No concerns	Low risk	No concerns	Major concerns	No concerns	Some concerns	Low
Glycocalyx+SoC:Vitamin C+SoC	0	No concerns	Low risk	No concerns	Major concerns	No concerns	Some concerns	Low
Glycocalyx+SoC:Vitamin D+SoC	0	No concerns	Low risk	No concerns	Major concerns	No concerns	Some concerns	Low
L+N+F+SoC:Melatonin+SoC	0	Major concerns	Low risk	No concerns	No concerns	Major concerns	Some concerns	Very low
L+N+F+SoC:Nanocurcumin+SoC	0	No concerns	Low risk	No concerns	No concerns	No concerns	Some concerns	Moderate
L+N+F+SoC:Vitamin B+SoC	0	No concerns	Low risk	No concerns	No concerns	Major concerns	Some concerns	Low
L+N+F+SoC:Vitamin C+SoC	0	Major concerns	Low risk	No concerns	No concerns	Major concerns	Some concerns	Very low

L+N+F+SoC:Vitamin D+SoC	0	No concerns	Low risk	No concerns	No concerns	No concerns	Some concerns	Moderate
Melatonin+SoC:Nanocurcumin+SoC	0	No concerns	Low risk	No concerns	No concerns	No concerns	Some concerns	Moderate
Melatonin+SoC:Vitamin B+SoC	0	No concerns	Low risk	No concerns	Major concerns	No concerns	Some concerns	Low
Melatonin+SoC:Vitamin C+SoC	0	No concerns	Low risk	No concerns	Major concerns	No concerns	Some concerns	Low
Melatonin+SoC:Vitamin D+SoC	0	No concerns	Low risk	No concerns	Major concerns	No concerns	Some concerns	Low
Nanocurcumin+SoC:Vitamin B+SoC	0	No concerns	Low risk	No concerns	No concerns	No concerns	Some concerns	Moderate
Nanocurcumin+SoC:Vitamin C+SoC	0	No concerns	Low risk	No concerns	No concerns	No concerns	Some concerns	Moderate
Nanocurcumin+SoC:Vitamin D+SoC	0	No concerns	Low risk	No concerns	No concerns	No concerns	Some concerns	Moderate
Vitamin B+SoC:Vitamin C+SoC	0	No concerns	Low risk	No concerns	Major concerns	No concerns	Some concerns	Low
Vitamin B+SoC:Vitamin D+SoC	0	No concerns	Low risk	No concerns	Major concerns	No concerns	Some concerns	Low
Vitamin C+SoC:Vitamin D+SoC	0	No concerns	Low risk	No concerns	Major concerns	No concerns	Some concerns	Low
