# Supplementary material for: Efficacy of supplementation with nutrients and dietary supplements on recovery and inflammatory response among patients with COVID-19: a network meta-analysis
Source: Front Nutr. 2026 Jul 28;13:1825539. doi: 10.3389/fnut.2026.1825539 (PMC13457122; doi:10.3389/fnut.2026.1825539)
Supplement: Supplementary file 5 [file Supplementary_file_5.pdf]

Supplementary Material 5: Supplementary analysis and model assessment for secondary outcomes (league tables) and list of abbreviations

Table S1A: League table for the comparison of the effects of different interventions versus SoC on the number of patients with cough

SoC					
0.39 (0.05, 2.7)	Nanocurcumin+SoC				
0.12 (0, 1.54)	0.3 (0.01, 8.09)	Melatonin +SoC			
0.71 (0.11, 4.54)	1.8 (0.13, 27.6)	5.94 (0.25, 319.71)	Vitamin D + SoC		
0.72 (0.26, 2.06)	1.84 (0.21, 17.73)	6 (0.38, 248.65)	1.02 (0.12, 8.6)	Quercetin+SoC	
1.05 (0.18, 6.23)	2.68 (0.19, 38.51)	8.89 (0.4, 448.23)	1.49 (0.11, 19.15)	1.46 (0.18, 11.42)	Curcumin+Quercetin+SoC

The data in the table are the RR (95% credible interval) of the intervention in the row compared with the intervention in the column. A larger RR represents a higher Cough league table; 1 represents no difference between the two interventions. Bolding represents a statistically significant decrease in the Cough league table.

Table S1B: League table for number of patients with dyspnea. The data in the table are the RR (95% credible interval) of the intervention in the row compared with the intervention in the column. A larger RR represents a higher Dyspnea; 1 represents no difference between the two interventions. Bolding represents a statistically significant decrease in Dyspnea.

**SoC**			
0.93(0.53,1.63)	**Vitamin D +**		

	**SoC**		
0.74(0.31,1.72)	0.8 (0.29,2.2)	**Quercetin+SoC**	
1.11(0.47,2.66)	1.2 (0.43,3.37)	1.5 (0.45,5.03)	**Curcumin+Quercetin+SoC**

Network diagrams (Node size is proportional to the sample size, and edge thickness reflects the number of direct comparisons between interventions):

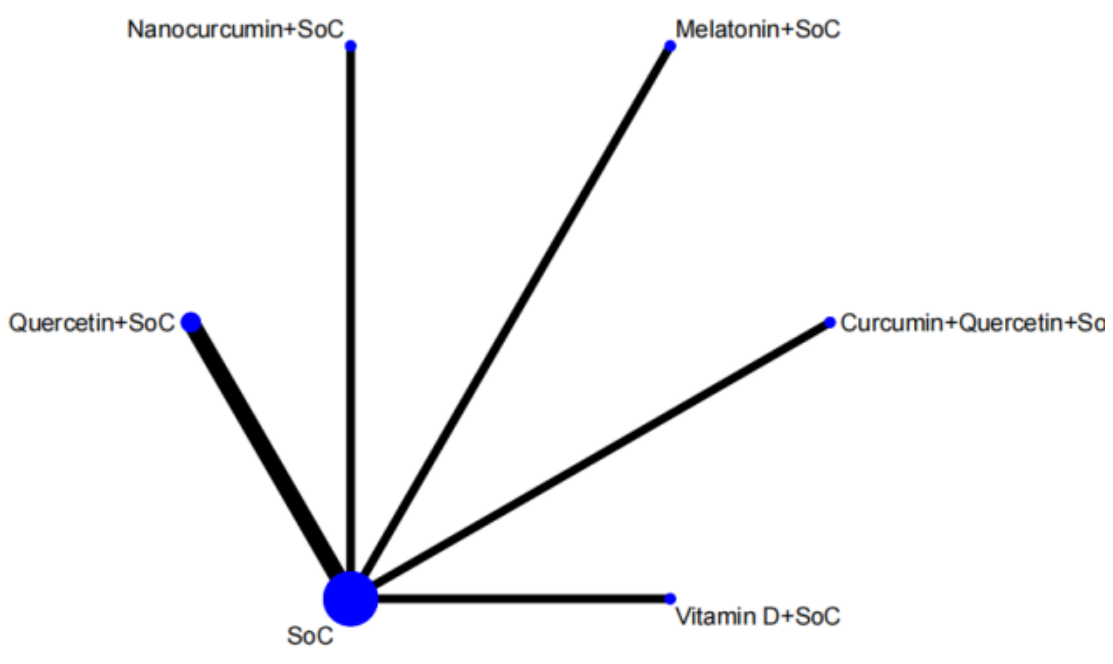

Figure S1A: Number of patients with cough

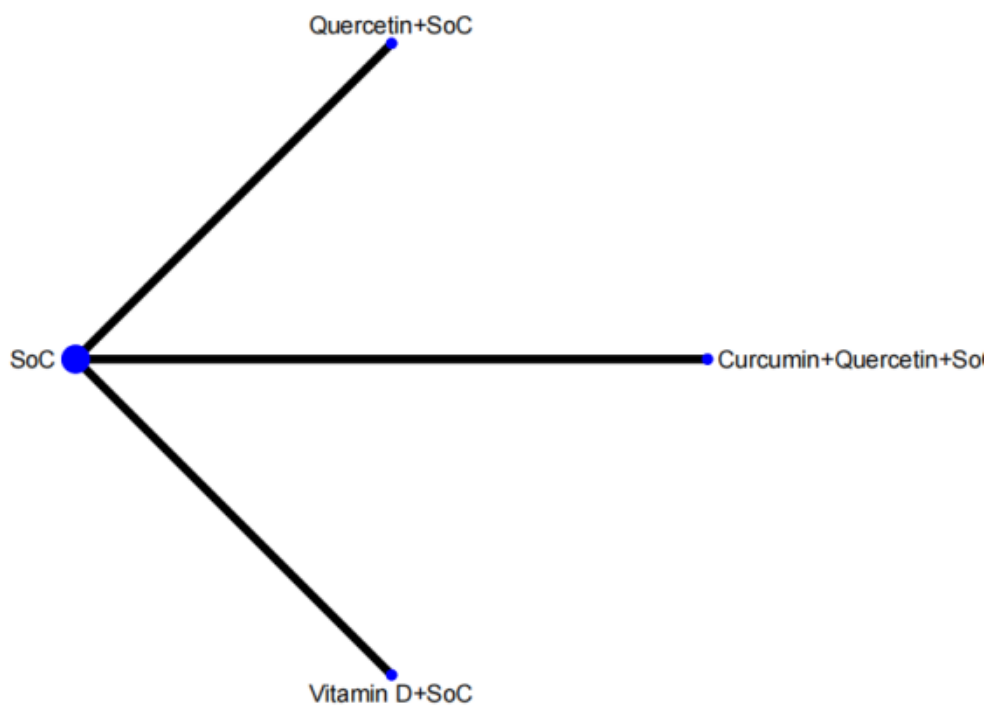

Figure S1B: Number of patients with dyspnea

Forest plots (The diamond represents the pooled effect size with its 95% confidence interval; squares denote individual study effects. Heterogeneity was assessed using the I^2 statistic and the chi-square test, with a reference line at the null effect):

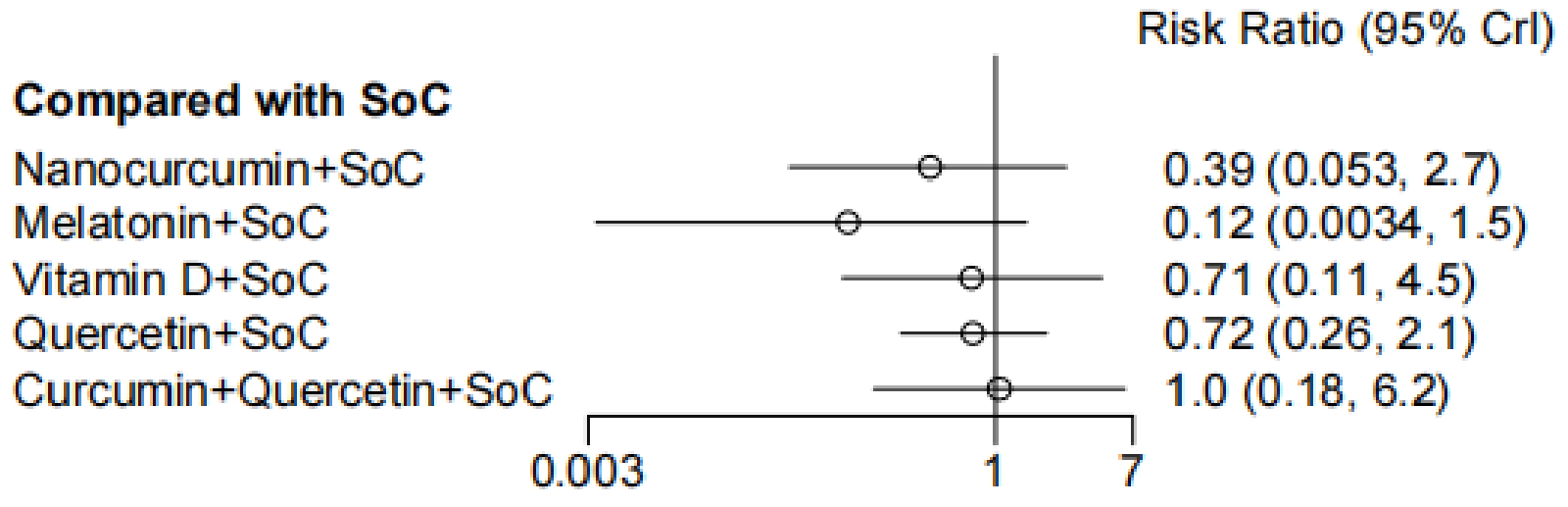

Figure S2A: Number of patients with cough

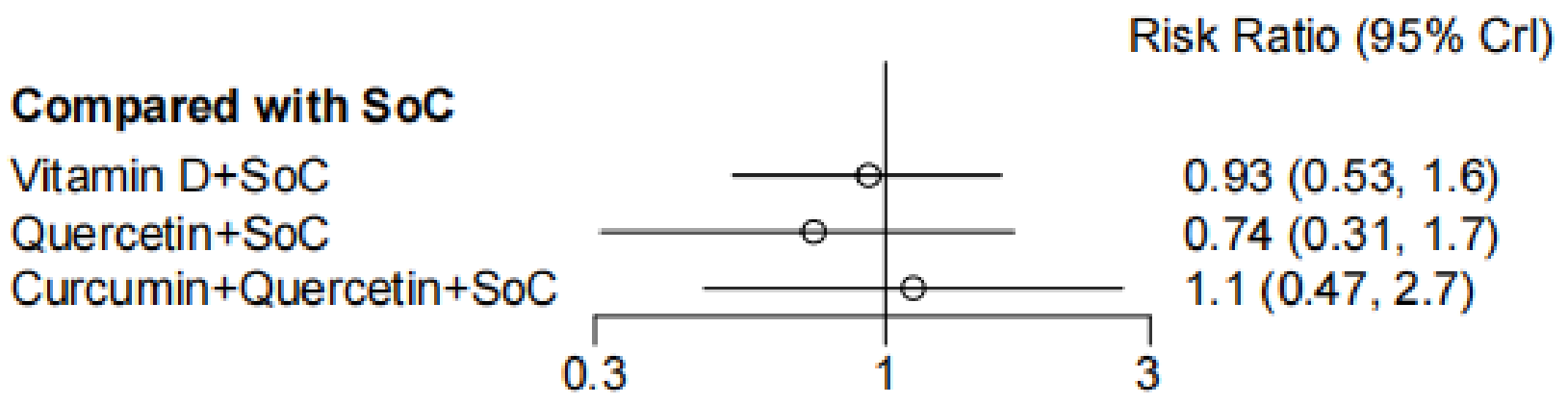

Figure S2B: Number of patients with dyspnea

SUCRA ranking plots (Bar height represents the SUCRA value, with higher bars indicating a superior treatment rank. The superimposed cumulative probability curves illustrate the ranking distributions, estimated from the specified model):

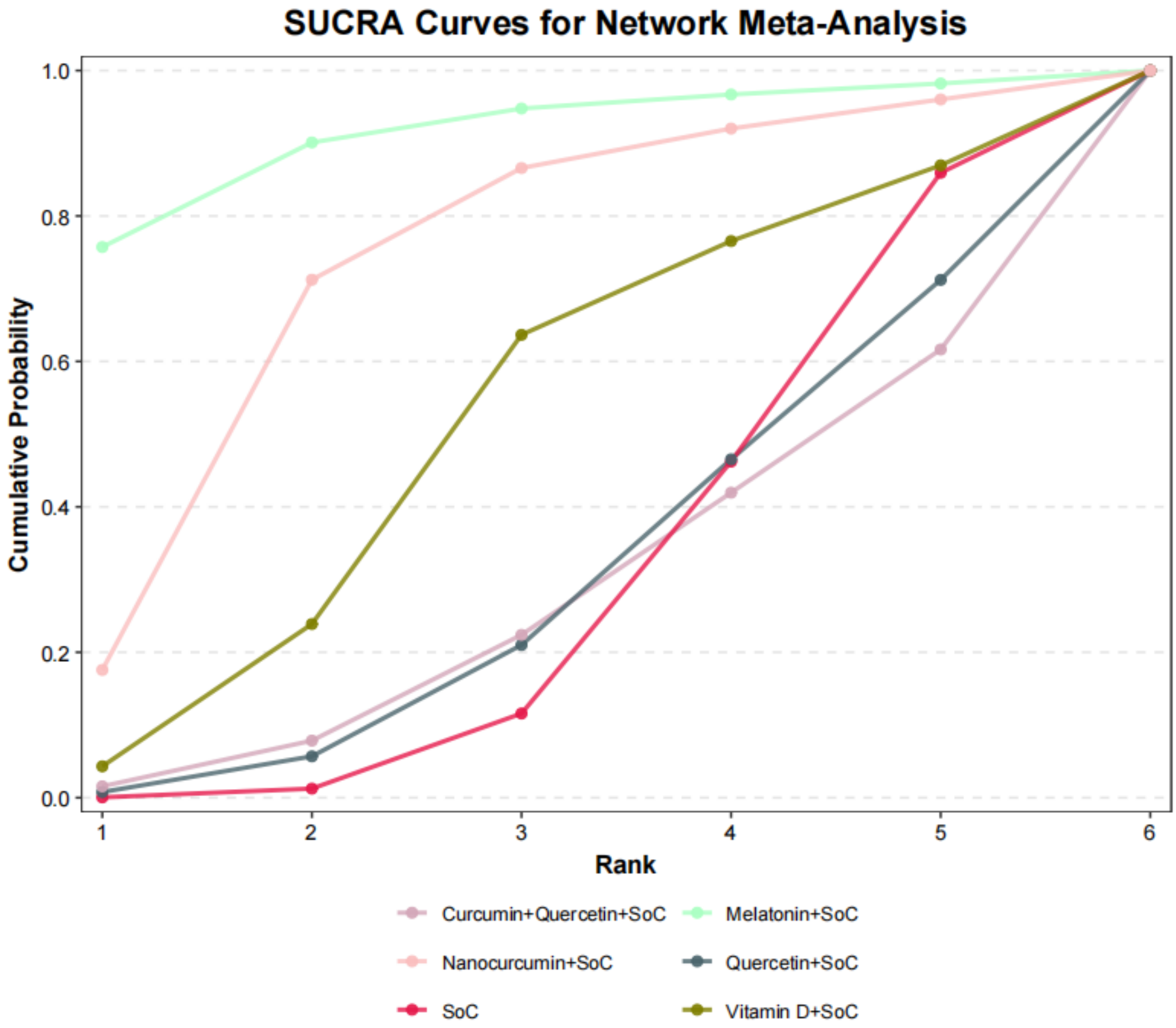

Figure S3A: Number of patients with cough

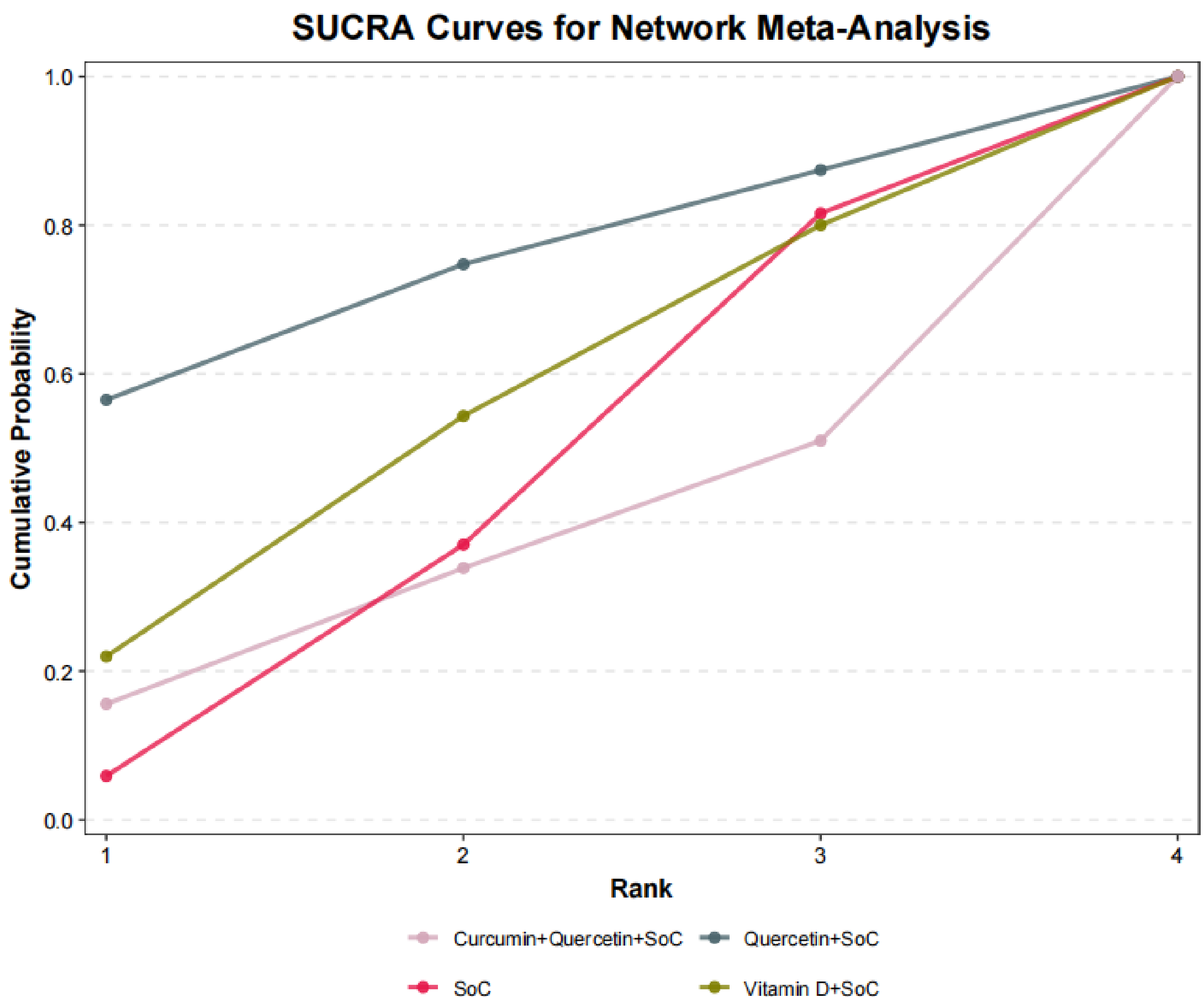

Figure S3B: Number of patients with dyspnea

Funnel plots (Each circle represents an included original study. The horizontal axis shows the effect size of each study relative to the common reference group, and the vertical axis shows the standard error (SE) of the effect size, with the vertical axis arranged in reverse order (namely, smaller SE values at the top stand for high-precision, large-sample studies, whereas larger SE values at the bottom represent low-precision, small-sample studies):

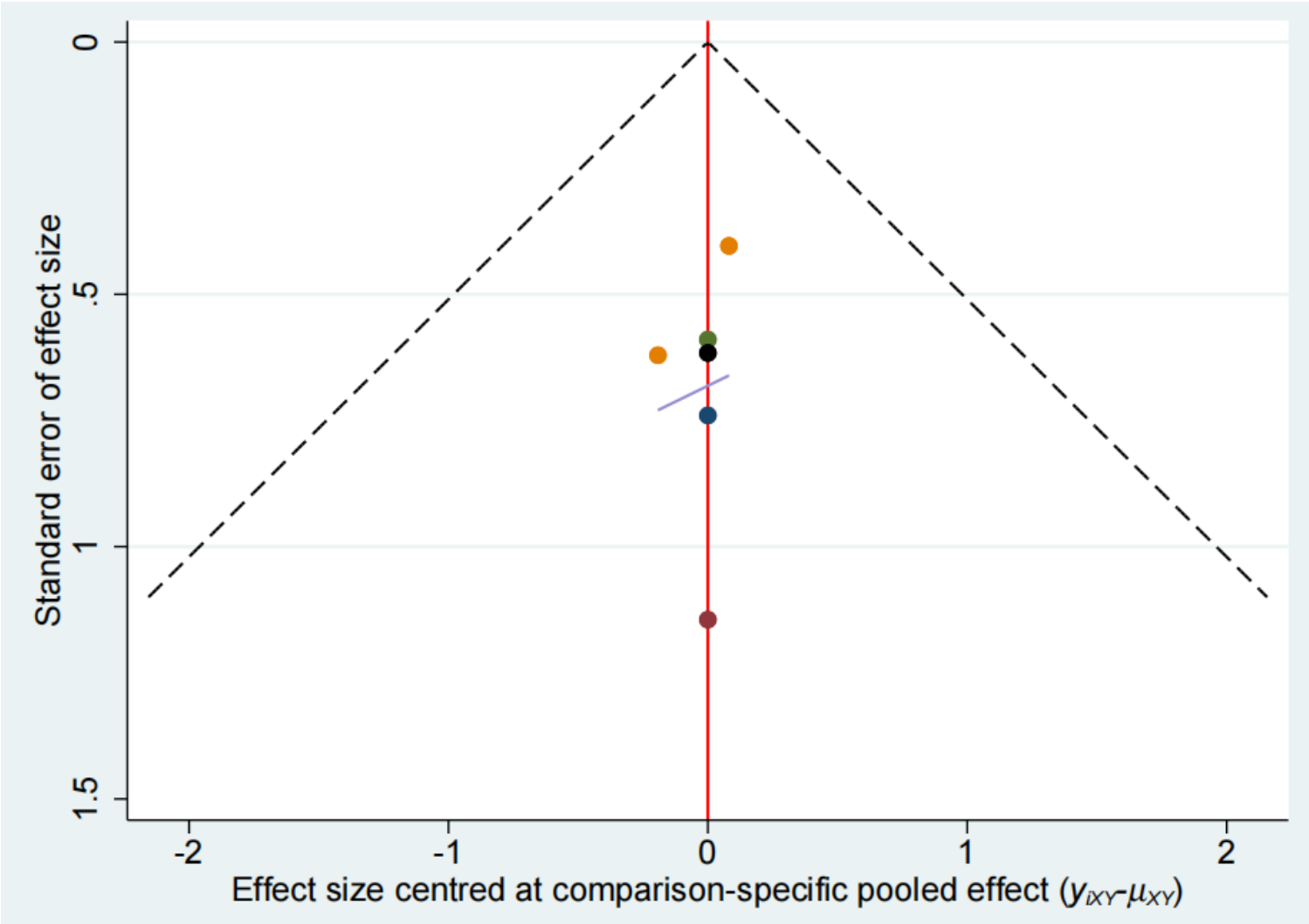

Figure S4A: Number of patients with cough

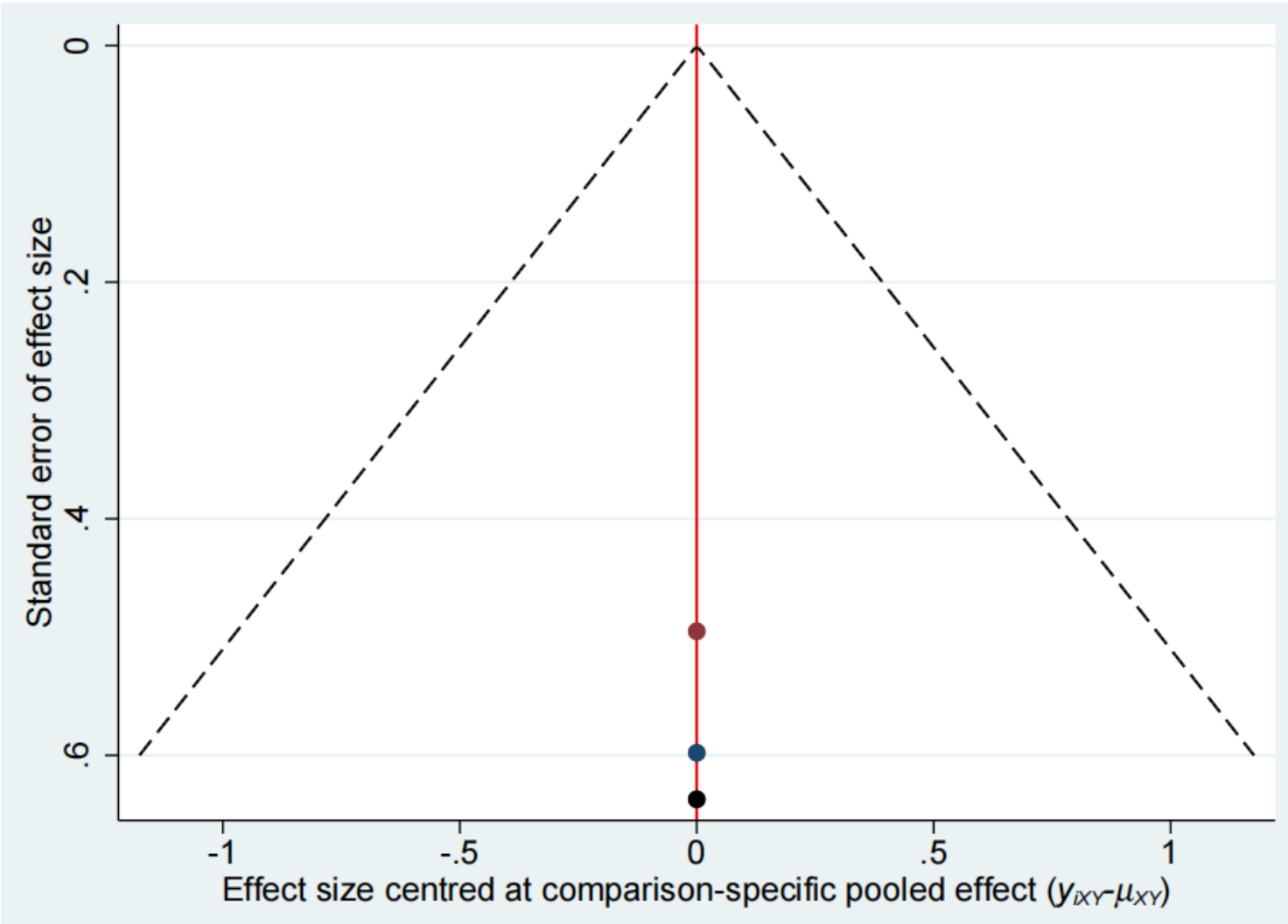

Figure S4B: Number of patients with dyspnea

Table S2: List of Abbreviations

Abbreviation	Full Title
NMA	network meta-analysis
RCT	randomized controlled trial
SoC	standard of care
MD	mean difference
SMD	standardized mean difference
RR	risk ratio
CrI	credible interval
PSRF	potential scale reduction factor
SUCRA	surface under the cumulative ranking curve

DIC	deviance information criterion
CINeMA	Confidence in Network Meta-Analysis
SE	standard error
NLR	neutrophil-to-lymphocyte ratio
WBC	white blood cell
